# Prioritization of Candidate miRNA Regulators Targeting Fibrotic–Immune Remodeling in Ligamentum Flavum Hypertrophy: An Integrated mRNA–miRNA Transcriptomic Study

**DOI:** 10.3390/biomedicines14071614

**Published:** 2026-07-17

**Authors:** Sevim Ondul, Kadir Oznam, Tamer Tamdogan, Muharrem Furkan Yuzbasi, Ibrahim Yilmaz

**Affiliations:** 1 Department of Neurosurgery, Giresun University Faculty of Medicine, Giresun 28200, Türkiye; 2 Department of Orthopedics and Traumatology, Istanbul Medipol University Faculty of Medicine, Istanbul 34295, Türkiye; 3 Department of Neurosurgery, Kahramanmaras Sutcu Imam University Faculty of Medicine, Kahramanmaras 46040, Türkiye; 4 Unit of Pharmacovigilance, Dr. Ismail Fehmi Cumalioglu City Hospital, Ministry of Health of the Republic of Türkiye, Tekirdag 59020, Türkiye

**Keywords:** ligamentum flavum hypertrophy, lumbar spinal stenosis, microRNA, single-cell RNA sequencing, extracellular matrix remodeling, fibrotic–immune remodeling, mRNA–miRNA integration, regulatory network, candidate miRNA regulator prioritization, transcriptomics

## Abstract

**Background:** Ligamentum flavum hypertrophy (LFH) is a major structural contributor to lumbar spinal stenosis and is characterized by extracellular matrix (ECM) remodeling with an increasingly recognized immune-associated component. However, the regulatory architecture linking disease-associated microRNA (miRNA) dysregulation to LFH transcriptomic remodeling remains incompletely defined. **Methods:** Public Gene Expression Omnibus datasets were analyzed using an integrated mRNA–miRNA transcriptomic framework. Single-cell RNA sequencing (GSE294458) was used to characterize the cellular landscape of hypertrophic and non-hypertrophic ligamentum flavum, whereas bulk transcriptomic analysis (GSE113212) identified LFH-associated differentially expressed genes. Differentially expressed miRNAs from an ossified ligamentum flavum dataset (GSE106256) were integrated with LFH-associated mRNA profiles through inverse miRNA–mRNA regulatory filtering. Functional enrichment, STRING protein–protein interaction (PPI) analysis, cytoHubba hub gene prioritization, LASSO regression, ROC analysis, remodeling-signature scoring, DGIdb, ChEA, and database-supported miRNA–target annotation were subsequently performed. **Results:** Single-cell analysis supported fibroblast-, myofibroblast-, and ECM-associated remodeling in hypertrophic ligamentum flavum. Bulk analysis identified nine significant differentially expressed genes, and integration with 33 dysregulated miRNAs generated 651 inverse-regulated core genes. Enrichment analyses highlighted ECM organization, proteoglycan/glycosaminoglycan (GAG) biology, immune cell differentiation, antigen presentation, and NF-κB/Wnt-related pathways. The STRING network included 650 nodes and 547 edges, with significant PPI enrichment (*p* = 2.04 × 10^−14^). LASSO prioritized PABPC1 and RPL4 as exploratory candidate hub features showing apparent discovery-cohort discrimination (AUC = 1.00); supplementary uncertainty, internal-stability, and separation-aware sensitivity analyses supported interpretation of this finding as a discovery-cohort signal rather than as independent diagnostic validation. Remodeling-signature analyses showed increased ECM fibrosis and proteoglycan/GAG scores, with inverse associations involving *PABPC1* and *RPL4*. Multi-layer prioritization identified hsa-miR-708-5p as the leading candidate, followed by hsa-miR-23b-3p, hsa-miR-191-5p, hsa-miR-181a-5p, and hsa-miR-653-5p. **Conclusions:** This integrated mRNA–miRNA transcriptomic analysis delineated a coordinated fibrotic–immune remodeling landscape in LFH and prioritized experimentally testable miRNA candidates linked to network-central regulatory pathways.

## 1. Introduction

Lumbar spinal stenosis (LSS) is a leading cause of pain, neurological dysfunction, and functional disability among the elderly population [1], and its prevalence is expected to rise substantially with global demographic aging [2]. Among the structural contributors to LSS, hypertrophy of the ligamentum flavum (LFH) occupies a central pathological role. The ligamentum flavum is a bilateral elastic ligament that lines the posterior spinal canal; under pathological conditions, it undergoes progressive thickening driven by aberrant extracellular matrix (ECM) accumulation, elastic fiber degradation, and collagen deposition, ultimately compressing neural elements and producing the characteristic symptomatology of LSS [3,4].

The molecular basis of LFH has been studied with increasing precision over the past decade. Transforming growth factor beta-1 (TGF-β1) has emerged as a central pro-fibrotic mediator, driving ligamentum flavum fibroblast-to-myofibroblast transition through the canonical SMAD2/3 signaling axis and promoting downstream overexpression of collagen I, collagen III, and fibronectin [4,5]. Mechanical stress amplifies this cascade by activating calcineurin/NFAT pathways and inducing local macrophage infiltration, which in turn further stimulates TGF-β1 secretion and sustains a self-reinforcing fibrotic loop [6]. GSK-3β/β-catenin signaling has been identified as an additional molecular event linking myofibroblast differentiation to ligamentum flavum thickening, with phosphorylated GSK-3β levels correlating positively with tissue thickness in LSS patients [7]. More recently, the SERPINE2/β-catenin axis has been shown to propagate fibrosis downstream of TGF-β1/SMAD3 signaling, and cartilage intermediate layer protein (CILP) has been reported to act as a negative regulator of this pathway in LFH tissue, broadening the molecular landscape of potential therapeutic targets [5,8].

Beyond the fibrotic dimension, an immune-regulatory component of LFH has become increasingly apparent. Single-cell RNA sequencing (scRNA-seq) studies have demonstrated that hypertrophic ligamentum flavum harbors distinct stromal and immune cell populations, including ACTA2-expressing myofibroblast-like cells, COL1A1/COL1A2-enriched fibroblast clusters, and infiltrating myeloid cells [9,10]. SPP1-positive macrophages have been identified as key cellular mediators that interact with stromal cell populations to drive fibrotic progression and ligament calcification, implicating immune–stromal crosstalk as a mechanistically important dimension of LFH pathogenesis [6,10]. Despite these advances, the majority of prior studies have characterized either the fibrotic or the immune component of LFH in isolation, and an integrated view of how these two programs are co-regulated at the transcriptomic level remains largely unavailable.

MicroRNAs (miRNAs) have emerged as promising candidate regulators of fibrotic and immune remodeling programs across multiple tissue contexts, including spinal degenerative disease. In the ligamentum flavum, miR-335-3p has been reported to suppress TGF-β1-induced fibrosis through epigenetic regulation of the SERPINE2/β-catenin pathway, and several additional miRNAs have been implicated in controlling collagen synthesis, ECM turnover, and myofibroblast activity [7]. However, the regulatory landscape linking disease-associated miRNA dysregulation to the specific transcriptomic alterations that define LFH—including fibrosis-associated, proteoglycan/glycosaminoglycan (GAG)-related, antigen-presentation, and immune–inflammatory programs—has not been systematically characterized through an integrated multi-dataset framework.

Several important gaps in the existing literature motivate the present study. First, prior transcriptomic investigations of LFH have predominantly relied on single-layer analyses, either bulk gene expression or single-cell RNA sequencing alone, without integrating these complementary perspectives into a unified regulatory framework. Second, the regulatory architecture connecting LFH-associated mRNA expression profiles with disease-associated miRNAs through inverse-expression filtering remains poorly defined. Third, although individual miRNAs have been linked to specific fibrotic mediators in LFH, network-level prioritization integrating protein–protein interaction (PPI) analysis, hub gene identification, LASSO-based candidate-feature prioritization, and remodeling-signature assessment has not been systematically applied to identify topologically central targets of disease-associated miRNAs. Fourth, the extent to which fibrosis-, proteoglycan/GAG-, antigen-presentation-, and immune-associated transcriptional programs are coordinately represented in LFH, and whether these programs are associated with candidate regulatory miRNAs, remains incompletely characterized. Finally, pharmacologic target annotation, upstream transcription-factor enrichment, database-supported miRNA–target annotation, and ranked-transcriptome pathway analysis have not been integrated into a single LFH-focused regulatory framework.

To address these gaps, an integrated transcriptomic study was designed using publicly available human datasets accessed from the Gene Expression Omnibus database. Single-cell RNA sequencing data from GSE294458 were analyzed to characterize the cellular and transcriptional landscape of hypertrophic and non-hypertrophic ligamentum flavum tissues. Bulk transcriptomic data from GSE113212 were used to identify LFH-associated gene-expression alterations and to support downstream enrichment, PPI network construction, hub gene prioritization, LASSO regression, ROC analysis, gene set enrichment analysis, and remodeling-signature evaluation. Dysregulated miRNAs from GSE106256 were integrated with LFH-associated mRNA profiles through an inverse miRNA–mRNA regulatory framework. GSE106253 and GSE267819 were included only as supplementary supportive evidence layers. Hub genes were further annotated using DGIdb, upstream transcription-factor enrichment was evaluated using ChEA, and database-supported miRNA–target annotation was performed using multiMiR.

The central hypothesis is that LFH is characterized by coordinated fibrotic and immune remodeling programs associated with disease-related miRNA regulation. The specific aim of this study is to integrate cellular-resolution transcriptomic context, bulk mRNA remodeling, disease-associated miRNA dysregulation, inverse miRNA–mRNA regulatory filtering, network topology, remodeling-signature analysis, and database-supported regulatory annotation to prioritize experimentally testable miRNA candidates linked to fibrotic–immune remodeling in LFH.

## 2. Materials and Methods

### 2.1. Study Design and Analytical Workflow

This study was designed as an integrated transcriptomic and miRNA-regulatory investigation to characterize the molecular landscape of ligamentum flavum hypertrophy (LFH) and to identify disease-associated miRNAs potentially involved in fibrotic–immune remodeling. Rather than focusing on a single differential-expression layer, the analytical framework was based on the integration of single-cell transcriptomic, bulk transcriptomic, and miRNA-expression datasets to define regulatory pathways associated with LFH pathogenesis.

The overall workflow consisted of four sequential analytical stages. First, a single-cell RNA-sequencing dataset (GSE294458) obtained from the Gene Expression Omnibus (GEO) database was analyzed to characterize the cellular composition of hypertrophic and non-hypertrophic ligamentum flavum tissues and to evaluate transcriptional remodeling at cellular resolution [9,11]. Particular emphasis was placed on fibroblast-, myofibroblast-, endothelial-, ECM-, and immune-related cellular populations. GSE294458 was accessed on 7 June 2026.

Second, a bulk transcriptomic dataset (GSE113212) was analyzed to identify differentially expressed genes (DEGs) associated with LFH [12]. Differential-expression results were subsequently used for functional enrichment analyses, PPI network construction, hub gene identification, LASSO-based candidate-feature prioritization, and pathway-level remodeling analyses. GSE113212 was accessed on 10 June 2026.

Third, an independent miRNA-expression dataset (GSE106256) was analyzed to identify differentially expressed miRNAs (DEMs) associated with ligament remodeling [13,14,15]. Because no publicly available LFH-specific miRNA dataset with suitable characteristics was identified, an ossified ligamentum flavum (OLF) miRNA dataset was used as a complementary regulatory resource. Dysregulated miRNAs were integrated with LFH-associated mRNA signatures using an inverse-expression framework to identify candidate regulatory interactions. GSE106256 was accessed on 18 June 2026.

In addition, GSE106253 was analyzed as a supplementary supportive transcript-level dataset to evaluate broader transcriptional alterations associated with ligament ossification and remodeling [13,15]. Because GSE106253 represents ossified rather than hypertrophic ligamentum flavum tissue, the dataset was used only as supportive evidence and was not included in primary LFH discovery analyses, hub gene prioritization, LASSO and ROC analyses, or mechanistic inference. GSE106253 was accessed on 18 June 2026.

**Figure 1:**
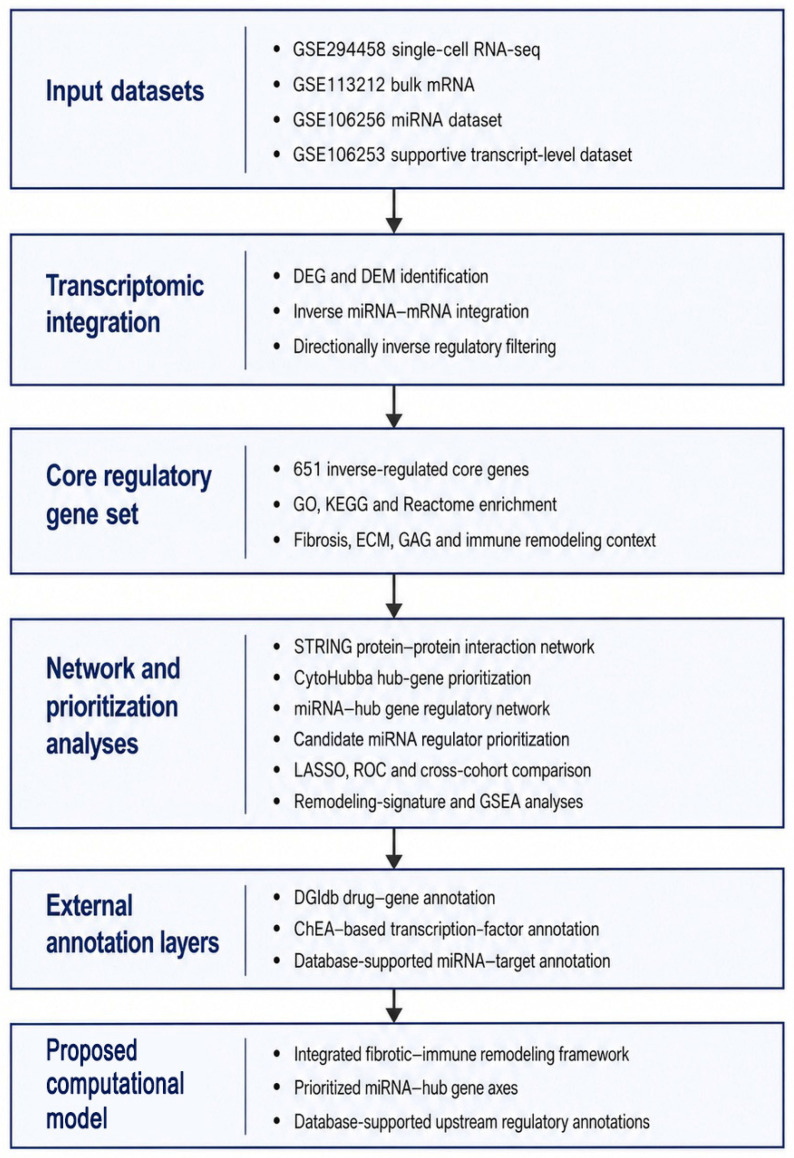
Overall analytical workflow of the study. The 651 inverse-regulated core-gene set shown in the workflow was generated through miRNA-target retrieval and inverse-expression filtering of LFH-associated mRNA profiles; it should not be interpreted as an expansion of the nine significant DEGs identified in the primary GSE113212 bulk comparison.

Following miRNA–mRNA integration, inverse-regulated core genes were subjected to Gene Ontology (GO), Kyoto Encyclopedia of Genes and Genomes (KEGG), and Reactome enrichment analyses. PPI networks were constructed using the STRING database and further analyzed in Cytoscape (version 3.10.4) to identify topologically important hub genes. Hub gene-centered miRNA regulatory networks were subsequently generated using miRNA–target information retrieved through multiMiR. Least absolute shrinkage and selection operator (LASSO) regression together with receiver operating characteristic (ROC) analysis were performed to identify informative gene features. Finally, fibrosis-, proteoglycan-, antigen-presentation-, and immune-associated remodeling signatures were integrated with network-level findings to prioritize disease-associated miRNAs and to construct a proposed computational model of LFH-associated fibrotic–immune remodeling. The overall analytical workflow is summarized in Figure 1. The analyses were classified as primary, supportive, or exploratory according to their role in the workflow. The primary discovery framework comprised differential mRNA analysis in GSE113212, differential miRNA analysis in GSE106256, and inverse-direction miRNA–mRNA integration used to define the core interaction set. Single-cell contextualization using GSE294458; supplementary analyses using GSE106253 and GSE267819; GO, KEGG, Reactome, and GSEA; STRING/PPI and cytoHubba analyses; remodeling-signature scoring and Spearman correlation analysis; and DGIdb, ChEA, and multiMiR annotations were considered supportive analyses providing cellular, functional, network, or database context. LASSO/ROC assessment, non-weighted miRNA prioritization, and the proposed computational model were treated as exploratory, hypothesis-generating analyses. Supportive and exploratory findings were not used to alter the upstream differential-expression criteria or the composition of the core miRNA–mRNA interaction set.

### 2.2. Single-Cell Transcriptomic Dataset and Preprocessing

Single-cell RNA-sequencing data from GSE294458 were analyzed to characterize the cellular and transcriptional landscape of ligamentum flavum tissues [9,11]. The dataset included one hypertrophic ligamentum flavum specimen obtained from a patient with lumbar spinal stenosis (LSS_HLF; LSS-associated hypertrophic ligamentum flavum) and one non-hypertrophic ligamentum flavum specimen obtained from a patient with lumbar disk herniation (LDH_NLF; LDH-associated non-hypertrophic ligamentum flavum). Accordingly, single-cell analyses were primarily intended to provide cellular-resolution insight into ligamentum flavum remodeling and to support biological interpretation of downstream transcriptomic findings.

Single-cell analyses were performed in R version 4.6.0 using the Seurat package (version 5.5.0) [16]. Raw count matrices were imported and subjected to quality control assessment based on the number of detected genes per cell (nFeature_RNA), total transcript counts per cell (nCount_RNA), and the proportion of mitochondrial transcripts (percent.mt). Cells were retained using the following predefined quality control thresholds: nFeature_RNA > 200, nFeature_RNA < 6000, and percent.mt < 15%. nCount_RNA was evaluated as a quality control metric but was not used as an additional filtering cutoff. Cells not meeting these criteria were excluded prior to downstream analyses. Following quality filtering, expression data were normalized and highly variable features were identified for dimensionality reduction and clustering analyses.

Principal component analysis (PCA) was performed using variable genes, and graph-based clustering was subsequently applied to identify transcriptionally distinct cellular populations. Uniform manifold approximation and projection (UMAP) was used to visualize the single-cell transcriptional landscape and cluster distribution patterns.

To facilitate biological interpretation, cellular populations were interpreted using marker-based annotation based on established lineage-associated and functionally relevant marker genes. Fibroblast and ECM-associated populations were evaluated using markers including COL1A1, COL1A2, COL3A1, DCN, LUM, and PDGFRA. Contractile and myofibroblast-associated populations were assessed using ACTA2, TAGLN, and MYL9, whereas endothelial populations were evaluated using PECAM1, VWF, KDR, and ENG. Additional cluster-specific marker genes were identified to support the annotation of immune and stromal cellular populations.

In addition, an exploratory all-cell differential-expression comparison between LSS_HLF and LDH_NLF cells was performed to identify sample-associated transcriptional signatures. Biologically relevant genes were selected from significant DEGs to summarize ECM, stromal, fibrosis-associated, immune, inflammatory, and myeloid-associated expression patterns. Cluster composition, marker-gene expression patterns, and transcriptional distributions were subsequently examined to provide an exploratory overview of cellular remodeling associated with hypertrophic ligamentum flavum tissue. Because GSE294458 contains a single specimen in each study group, all disease-group comparisons derived from this dataset were interpreted as exploratory and sample-associated observations rather than population-level estimates.

### 2.3. Bulk Transcriptomic Dataset and Differential Gene-Expression Analysis

The GSE113212 dataset was used as the primary bulk transcriptomic dataset to characterize LFH-associated gene-expression alterations [12]. The analysis included eight ligamentum flavum samples: four older hypertrophic ligamentum flavum samples (Old/LFH) and four younger non-hypertrophic control ligamentum flavum samples (Young/control). Throughout the manuscript, Old/LFH denotes the older hypertrophic group, whereas Young/control denotes the younger non-hypertrophic control group. Because age category and hypertrophic status were aligned in this cohort, GSE113212 was analyzed as an Old/LFH versus Young/control comparison rather than as an age-matched LFH-versus-control design. As this dataset provided sample-level bulk transcriptomic information, it was used for differential gene-expression analysis, functional enrichment, network construction, hub gene prioritization, LASSO and ROC analyses, and remodeling-signature analyses.

Expression data were processed in R, and differential gene-expression analysis was performed using the limma package (version 3.68.4) [17]. Group comparisons were conducted using moderated linear modeling. Multiple-testing correction was performed using the Benjamini–Hochberg false-discovery-rate method. DEGs were defined according to an adjusted *p* value < 0.05 and an absolute log2 fold change > 1. Probe-level features were annotated to gene symbols using the available platform annotation where applicable.

Significantly upregulated and downregulated genes were used to define the bulk transcriptomic remodeling landscape of LFH. Volcano plots and heatmaps were generated to visualize the distribution and expression patterns of DEGs. The resulting LFH-associated gene-expression layer was subsequently integrated with dysregulated miRNAs to identify inverse miRNA–mRNA regulatory relationships and to support downstream enrichment, PPI-network, hub gene, LASSO, ROC, and remodeling-signature analyses.

### 2.4. Supportive Transcript-Level Remodeling Analysis

GSE106253 was analyzed as a supplementary supportive dataset to evaluate broader transcriptional alterations associated with ligament remodeling [13,15]. The dataset compared OLF tissues with normal ligamentum flavum tissues and was examined to determine whether ligament pathology is accompanied by transcript-level molecular perturbations beyond those identified in the primary LFH datasets.

Differential transcript-expression analysis was performed using the same analytical framework applied to the primary bulk transcriptomic dataset. Differentially expressed transcript features were summarized to provide an overview of remodeling-associated transcriptional alterations in OLF tissue.

Because GSE106253 represents ossified rather than hypertrophic ligamentum flavum tissue, the dataset was not incorporated into primary LFH discovery analyses, inverse miRNA–mRNA integration, hub gene prioritization, LASSO/ROC evaluation, or mechanistic inference. Instead, the findings were interpreted as supportive transcript-level evidence of ligament-remodeling-associated molecular alterations and were used solely to provide additional biological context for the primary LFH transcriptomic analyses.

### 2.5. Supplementary LFH-Only Single-Cell Marker Assessment

An additional LFH-only single-cell RNA-sequencing dataset (GSE267819) was analyzed as a supplementary resource to evaluate marker-level consistency across independent LFH samples. Because no non-LFH control group was available, the dataset was used exclusively for quality control assessment, cluster visualization, and marker-expression analyses. GSE267819 was not included in differential-expression analyses, miRNA–mRNA integration, enrichment analyses, PPI network construction, hub gene prioritization, LASSO/ROC analyses, or mechanistic inference. GSE267819 was accessed on 18 June 2026. The resulting analyses were interpreted as supportive marker-level evidence and are presented in the Supplementary Materials.

### 2.6. miRNA Expression Dataset and Differential miRNA Analysis

GSE106256 was analyzed to identify dysregulated miRNAs associated with ligament remodeling [13,14]. The dataset compared OLF tissues with normal ligamentum flavum tissues and was used as a complementary regulatory dataset for downstream miRNA–mRNA integration analyses. Because no publicly available LFH-specific miRNA expression dataset with suitable characteristics was identified, GSE106256 was utilized to explore disease-associated miRNAs potentially involved in ligament-remodeling processes.

Raw miRNA count data were imported into R and subjected to differential expression analysis. Low-abundance miRNAs were filtered prior to statistical testing to reduce the influence of sparsely expressed features. Expression data were normalized, and differential miRNA-expression analysis was performed between OLF and normal ligamentum flavum samples using the edgeR package (version 4.10.1) [18].

Multiple-testing correction was performed using the Benjamini–Hochberg false-discovery-rate method. Differentially expressed miRNAs were identified according to adjusted *p* value and fold-change criteria and were subsequently classified as upregulated or downregulated.

Because GSE106256 represents OLF rather than LFH tissue, the resulting miRNA signatures were not interpreted as LFH-specific regulatory events. Instead, dysregulated miRNAs were used as a regulatory resource for subsequent integration with LFH-associated mRNA signatures to identify inverse miRNA–mRNA relationships potentially linked to fibrosis-, proteoglycan-, and immune-associated remodeling programs.

### 2.7. miRNA–mRNA Integration and Identification of Inverse-Regulated Core Genes

To identify regulatory genes potentially linked to ligamentum flavum remodeling, differentially expressed miRNAs from GSE106256 were first matched with their predicted and/or validated target genes retrieved using the multiMiR package [19], which integrates experimentally validated and computationally predicted miRNA–target interactions from multiple curated databases. All retrieved predicted and validated multiMiR target records were used at this stage, and no additional confidence-score or evidence-level cutoff was applied before inverse-expression filtering. Database-supported evidence categories were evaluated subsequently as an annotation layer and were not used to exclude miRNA–target pairs from the initial inverse-expression framework. Directional information from LFH-associated mRNA expression profiles in GSE113212 was then used to retain inverse miRNA–mRNA relationships based on the canonical inhibitory relationship between miRNAs and their target transcripts. Accordingly, upregulated miRNAs were paired with relatively downregulated target genes, whereas downregulated miRNAs were paired with relatively upregulated target genes. Only inverse miRNA–mRNA pairs satisfying these directional criteria were retained for downstream analyses.

The resulting inverse regulatory network was used to identify a core set of genes potentially regulated by dysregulated miRNAs within ligament-remodeling-associated molecular programs. This integration yielded 651 inverse-regulated core genes. Among these, 650 genes were successfully recognized and mapped for downstream enrichment and network analyses, including GO, KEGG, Reactome, STRING, and Cytoscape-based analyses. The full list of 651 genes was retained for reporting purposes, whereas the 650 mapped genes were used for downstream computational analyses. The inverse-regulated core gene set served as the principal regulatory framework for functional enrichment analysis, PPI network construction, hub gene prioritization, downstream LASSO/ROC evaluation of hub genes, and remodeling-signature analyses.

### 2.8. Functional Enrichment Analyses

Functional enrichment analyses were performed to characterize the biological processes and signaling pathways represented by the inverse-regulated core gene set. The 650 mapped genes were analyzed using the clusterProfiler package (version 4.20.0) [20]. GO Biological Process enrichment analysis was performed to identify overrepresented biological functions associated with the integrated regulatory gene set. In parallel, KEGG pathway enrichment analysis was performed using clusterProfiler [21]. Reactome pathway enrichment was obtained from the STRING functional enrichment output generated from the inverse-regulated core gene network and was used as a complementary pathway-level annotation [22].

Multiple-testing correction was performed using the Benjamini–Hochberg false-discovery-rate method, and significantly enriched terms were retained for downstream interpretation. Enrichment results were subsequently examined with particular emphasis on fibrosis-associated pathways, ECM remodeling, proteoglycan and GAG biology, immune regulation, inflammatory signaling, antigen presentation, and tissue-remodeling processes.

The resulting enrichment profiles were used to provide biological context for the inverse-regulated core genes and to support downstream network-based and regulatory analyses.

### 2.9. PPI Network Construction and Hub Gene Identification

To characterize potential functional connectivity among the inverse-regulated core genes, a PPI network was constructed using the STRING database (available online: https://string-db.org/; accessed on 20 June 2026) [23]. The 651 inverse-regulated core genes were submitted to STRING, and the analysis was restricted to Homo sapiens. A high-confidence interaction threshold was applied, and disconnected nodes were hidden during network visualization to facilitate interpretation of the connected interaction structure.

The resulting network contained 650 nodes and 547 edges, with an average node degree of 1.68, an average clustering coefficient of 0.319, and a PPI enrichment *p* value of 2.04 × 10^−14^. These parameters were used to describe the overall topology and enrichment of the interaction network.

The STRING-derived interaction network was imported into Cytoscape (available online: https://cytoscape.org/; accessed on 20 June 2026) for visualization and topological analysis [24]. Hub gene prioritization was performed using the cytoHubba plugin (available online: https://apps.cytoscape.org/apps/cytohubba; accessed on 20 June 2026) [25]. Maximal clique centrality was used as the principal ranking method to identify highly connected and topologically central genes within the PPI network. Additional centrality metrics, including degree, closeness, betweenness, and edge-percolated component scores, were assessed as supportive topological analyses.

Hub genes identified from the PPI network were not interpreted as experimentally validated disease drivers. Instead, they were used as network-prioritized candidates for downstream miRNA–hub gene regulatory network construction, LASSO-based candidate-feature prioritization, and remodeling-signature analyses. Accordingly, all network-derived prioritization results were interpreted as exploratory and hypothesis-generating rather than as evidence of causality or biological validation.

This integrated STRING–Cytoscape–cytoHubba workflow for network construction and hub gene prioritization follows the same computational framework that our group has previously applied across a range of transcriptomic and network-pharmacology studies [26,27,28,29].

### 2.10. Construction of the miRNA–Hub Gene Regulatory Network

To refine the regulatory network toward biologically central remodeling-associated targets, CytoHubba-prioritized hub genes were intersected with the inverse miRNA–mRNA interaction network. Only miRNA–gene pairs meeting both criteria were retained: inverse regulation between the dysregulated miRNA and its predicted or validated target gene, and inclusion of the target gene among the PPI-derived hub genes.

The resulting miRNA–hub gene regulatory network was used to identify dysregulated miRNAs potentially linked to topologically central genes within the LFH-associated remodeling network. Network relationships were visualized to summarize integrated transcriptomic evidence linking dysregulated miRNAs with prioritized hub genes involved in fibrosis, ECM remodeling, proteoglycan and GAG biology, antigen presentation, and immune-associated remodeling pathways.

This network was interpreted as a regulatory prioritization framework rather than as experimental validation of direct miRNA–target interactions. The inferred miRNA–hub gene relationships were therefore treated as hypothesis-generating and used to inform downstream LASSO-based feature assessment, remodeling-signature interpretation, and miRNA regulator prioritization. These relationships were derived from integrated transcriptomic evidence and were not regarded as experimentally validated.

### 2.11. LASSO Feature Selection, ROC Analysis, and Supplementary Sensitivity Assessment

To identify hub genes with candidate discriminatory relevance, expression profiles of the CytoHubba-prioritized hub genes were evaluated using LASSO regression. LASSO analysis was performed using the glmnet package (version 5.0) [30], with LFH status used as the outcome variable. Cross-validation was applied within the discovery cohort to guide regularization and to identify informative candidate gene features from the hub gene set. Genes selected through this procedure were subsequently used to construct an exploratory candidate-feature score within the GSE113212 dataset. ROC analysis was performed using the pROC package (version 1.19.0.1) [31] to evaluate apparent discovery-cohort discrimination. Area under the curve (AUC), sensitivity, and specificity were calculated as descriptive measures of exploratory model performance. Because GSE113212 contained a limited number of samples and no independent diagnostic-validation cohort was available for model development, the resulting analyses were interpreted as exploratory and hypothesis-generating rather than as evidence of clinically validated diagnostic biomarkers. The selected genes were subsequently incorporated into downstream regulatory and remodeling-signature analyses.

Supplementary uncertainty, internal-stability, and separation-aware sensitivity analyses were performed to evaluate the exploratory PABPC1–RPL4 candidate-feature score in GSE113212. This discovery cohort comprised eight samples, including four Old/LFH and four Young/control samples. PABPC1 and RPL4 were evaluated using their traceable platform probes. A predefined direction-aware PABPC1–RPL4 score was calculated such that lower PABPC1/RPL4 expression corresponded to a higher Old/LFH score. Discovery-cohort discrimination was summarized by ROC analysis, AUC, the Youden-optimal cutoff, and exact Clopper–Pearson confidence intervals for sensitivity and specificity. DeLong confidence intervals were also calculated for the apparent AUC.

Because the standard two-gene logistic model showed complete-separation behavior, standard maximum-likelihood coefficient estimates were not used for biological or diagnostic inference. Internal stability of the predefined PABPC1–RPL4 score was assessed using leave-one-out evaluation and all 24 possible balanced stratified four-fold allocations; in each four-fold allocation, each fold contained one Old/LFH and one Young/control sample, and scaling parameters were estimated from the training samples within each split. Feature selection was not repeated within these resampling analyses; therefore, the resampling results were interpreted as internal-stability assessments of the predefined PABPC1–RPL4 candidate-feature score rather than as nested model validation.

To further contextualize the small-sample behavior of the score, a class-stratified case-resampling bootstrap of the apparent score was performed.

Because resampling remained degenerate under complete separation, these bootstrap results were reported only as explanatory supplementary evidence and were not interpreted as formal uncertainty estimates. A separation-aware Firth bias-reduced logistic sensitivity analysis was additionally performed using Jeffreys-prior penalization with a Newton–Raphson algorithm and step-halving. Firth coefficients were reported descriptively, whereas Wald confidence intervals and Wald *p* values were not used for manuscript inference. In addition, perturbation-based sensitivity analyses were performed by applying graded Gaussian feature-level perturbations from 0% to 100% of the mean feature standard deviation. These perturbation results were interpreted as noise-sensitivity summaries rather than as formal AUC confidence intervals.

Collectively, these analyses assessed the uncertainty and internal stability of the exploratory PABPC1–RPL4 candidate-feature score. They do not constitute independent diagnostic validation.

### 2.12. Cross-Cohort Evaluation Strategy for LASSO-Selected Genes

To explore whether the LASSO-selected genes showed comparable expression patterns across independent transcriptomic layers, PABPC1 and RPL4 expression changes in the bulk dataset were compared with their expression patterns in the single-cell dataset. For GSE113212, group-level expression differences were derived from the bulk differential-expression analysis. For GSE294458, mean and median expression values were calculated across cells separately for the LSS_HLF and LDH_NLF specimens.

Because GSE113212 and GSE294458 differ in platform, biological design, sample structure, and analytical resolution, this analysis was interpreted as a cross-cohort comparison rather than formal external validation. Directional agreement or disagreement between datasets was used only to describe whether the LASSO-selected genes showed concordant or discordant expression patterns across the available data layers.

### 2.13. Remodeling Signature Analyses and Hub Gene Associations

To evaluate the biological context of the identified regulatory networks, predefined gene signatures representing ECM fibrosis, proteoglycan/GAG biology, immune-inflammatory processes, and antigen presentation were compiled from genes associated with the corresponding biological programs. For each sample, the signature score was calculated by averaging the gene-wise normalized expression values of all genes from the corresponding predefined signature that were present in the GSE113212 expression matrix. The complete gene membership of each remodeling signature is provided in the Supplementary Materials. Genes not detected or not annotated in the dataset were not included in score calculation. Signature scores were compared between study groups to evaluate remodeling-associated transcriptional programs in LFH. Differences in signature scores were assessed using two-sided Welch’s *t*-tests. These analyses were performed to determine whether LFH-associated transcriptional alterations were preferentially linked to fibrosis-, proteoglycan/GAG-, immune-, or antigen-presentation-related biological processes.

To further investigate the relationship between LASSO-selected candidate features and remodeling-associated molecular programs, expression levels of the LASSO-selected genes were compared between study groups. Associations between LASSO-selected genes and remodeling-signature scores were subsequently evaluated using Spearman correlation analysis across all samples. Correlation analyses were used to determine whether variation in hub gene expression was associated with relative changes in fibrosis-, proteoglycan/GAG-, immune-inflammatory-, and antigen-presentation-related transcriptional signatures.

The resulting signature scores and correlation profiles were interpreted as relative transcriptomic representations of biological processes and were used to provide biological context for the integrated regulatory network. These analyses were not intended to directly quantify cellular abundance, pathway activity, or causal molecular mechanisms.

### 2.14. Candidate miRNA Regulator Prioritization

To identify high-priority regulatory miRNAs potentially involved in LFH-associated fibrotic–immune remodeling, a multi-layer prioritization strategy was applied. Candidate miRNAs were evaluated according to their differential-expression status, participation in inverse miRNA–mRNA regulatory interactions, targeting of PPI-derived hub genes, association with LASSO-selected genes, and relationship to remodeling-associated biological programs identified through enrichment and signature analyses. Particular attention was given to miRNAs targeting genes implicated in fibrosis, ECM remodeling, proteoglycan/GAG biology, antigen presentation, and immune-associated processes. Regulatory candidates supported by multiple analytical layers were considered higher-priority miRNAs for downstream biological interpretation.

Dysregulated miRNAs were not ranked using a formal weighted scoring algorithm. Instead, prioritization was based on the convergence of evidence across independent analytical layers, including differential expression, inverse miRNA–mRNA regulation, hub gene targeting, association with LASSO-selected genes, and biological relevance to remodeling-associated pathways and signatures. The prioritization framework was intended to identify disease-associated regulatory candidates rather than experimentally validated therapeutic targets. Accordingly, the resulting miRNAs were interpreted as candidate regulatory miRNAs that may warrant future mechanistic and experimental investigation.

### 2.15. Database-Supported Annotation Strategy for Prioritized miRNA–Hub Gene Interactions

To provide an additional evidence-based annotation layer for the LFH regulatory network, database-supported miRNA–target interaction evidence was evaluated using the multiMiR package (version 1.34.0) in R. This analysis was performed after miRNA–hub gene network construction, hub gene prioritization, LASSO-based candidate-feature prioritization, remodeling-signature analyses, and candidate miRNA regulator prioritization.

Candidate miRNA–target interactions were derived exclusively from the integrated LFH regulatory framework generated in the present study, including the core miRNA–hub gene regulatory network, the complete miRNA–hub gene edge list, candidate miRNA regulator prioritization outputs, and the proposed computational model. No additional differential-expression analyses, target-prediction procedures, network reconstructions, hub gene prioritization steps, machine-learning analyses, enrichment analyses, or signature-scoring procedures were performed during this stage.

Database-supported annotations were retrieved using the multiMiR integrated database (database version 2.4.0; updated 28 August 2024), which aggregates experimentally curated and computationally predicted miRNA–target interaction resources. For each candidate miRNA–target pair, interaction records were queried against both validated and predicted databases available within the multiMiR framework. Experimentally curated resources included validated interaction repositories such as TarBase and miRTarBase when available, whereas prediction support was obtained from prediction databases incorporated within multiMiR.

For each interaction, the presence or absence of database-supported evidence was recorded separately for curated validated sources and prediction-based sources. Database names, evidence annotations, and database-reported evidence descriptors were retained exactly as returned by multiMiR. Each miRNA–target pair was then annotated according to the highest level of database support identified, distinguishing interactions with curated validated-database evidence from those supported only by prediction resources or lacking identifiable database support.

Particular attention was given to candidate regulatory miRNAs and interactions involving LASSO-selected genes and hub gene targets identified within the integrated LFH regulatory model. Candidate prioritization was performed according to the predefined analytical workflow described below. Importantly, this analysis was conducted solely as a database-derived regulatory annotation procedure and should not be interpreted as experimental validation of miRNA–target interactions in LFH. The resulting annotations were used to evaluate the degree of external database support for candidate regulatory relationships identified through the transcriptomic integration analyses and did not alter the underlying differential-expression, enrichment, network, hub gene, machine-learning, or signature-association results.

### 2.16. ChEA-Based Upstream Transcription-Factor Enrichment Analysis

To provide an additional database-derived upstream regulatory annotation layer for the LFH hub gene network, transcription-factor enrichment analysis was performed using the CytoHubba maximal clique centrality (MCC)-prioritized top 20 hub genes as input. The input gene set consisted of *RPL5*, *RPL3*, *RPL4*, *RPL9*, *RPL19*, *RPS6*, *RPS20*, *RPS27*, *RPS27A*, *CD74*, *EEF1A1*, *PABPC1*, *SMKR1*, *BUB1B*, *TOP2A*, *CEP55*, *KIF15*, *KIF14*, *HMMR*, and *DLGAP5*.

Enrichment analysis was conducted in R version 4.6.0 using the enrichR package (version 3.4) and the ChEA 2022 ChIP-based transcription-factor target gene set library. Significant enrichment terms were defined according to a Benjamini–Hochberg-adjusted *p* value < 0.05. For each enriched transcription-factor term, the dataset descriptor, overlap, nominal *p* value, adjusted *p* value, odds ratio, combined score, and overlapping genes were retained. To provide a supportive annotation layer, the same hub gene input list was also queried against the ENCODE and ChEA Consensus transcription-factor library.

This analysis was performed solely as a database-derived upstream regulatory annotation of MCC-prioritized hub genes. Because ChEA and ENCODE-ChEA libraries integrate ChIP-derived transcription-factor target sets generated across diverse species, cell types, and experimental contexts, the resulting enrichments were interpreted as candidate upstream regulatory annotations rather than direct evidence of transcription-factor activity in LFH tissue.

### 2.17. Statistical Analysis

All statistical analyses were performed in R version 4.6.0 (R Foundation for Statistical Computing, Vienna, Austria). Differential mRNA, transcript-level, and miRNA expression analyses were corrected for multiple testing using the Benjamini–Hochberg false-discovery-rate method. Unless otherwise specified, adjusted *p* values or FDR values below 0.05 were considered statistically significant.

For group comparisons of remodeling-signature scores and LASSO-selected gene expression levels, two-sided Welch’s *t*-tests were used. Associations between LASSO-selected genes and remodeling-signature scores were evaluated using Spearman correlation analysis. Apparent discovery-cohort discrimination was summarized using ROC analysis, AUC, sensitivity, specificity, and the Youden-optimal cutoff. Exact Clopper–Pearson 95% confidence intervals were calculated for sensitivity and specificity, and the DeLong method was used to estimate the 95% confidence interval for the AUC. Internal stability of the PABPC1–RPL4 score was assessed using leave-one-out evaluation and all 24 possible balanced stratified four-fold allocations. Complete-separation behavior was further examined using standard logistic regression and a Firth bias-reduced logistic sensitivity analysis. The class-stratified case-resampling bootstrap and graded feature-level perturbation analyses were interpreted descriptively as supplementary sensitivity assessments rather than as formal uncertainty estimates.

Given the limited sample sizes of the analyzed public datasets, all LASSO-based candidate-feature analyses, cross-cohort comparison, and regulatory prioritization analyses were interpreted as exploratory and hypothesis-generating. No causal, experimentally validated, or clinically validated biomarker claims were inferred from the computational analyses.

## 3. Results

### 3.1. Single-Cell Quality Control and Transcriptional Landscape of Ligamentum Flavum Tissues

**Figure 2:**
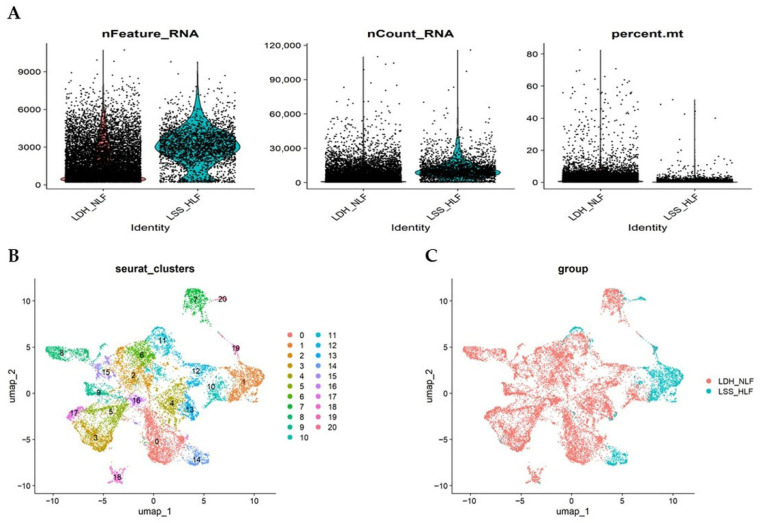
Quality control assessment and single-cell landscape of GSE294458 ligamentum flavum cells. (**A**) Violin plots showing the distribution of the number of detected genes per cell (nFeature_RNA), total UMI counts per cell (nCount_RNA), and mitochondrial transcript percentage (percent.mt) during quality control assessment in LDH_NLF and LSS_HLF samples. (**B**) UMAP visualization of quality-filtered cells colored by Seurat cluster identity. A total of 21 clusters were identified. (**C**) UMAP visualization of the same cells colored by disease group, showing the distribution of LDH_NLF and LSS_HLF cells across the single-cell transcriptional landscape.

Single-cell transcriptomic profiling of GSE294458 was performed to characterize the cellular landscape of hypertrophic and non-hypertrophic ligamentum flavum tissues. After quality control filtering, 15,184 high-quality cells were retained for downstream analyses, including 12,610 cells from the LDH_NLF sample and 2574 cells from the LSS_HLF sample (Table S1). Quality control assessment demonstrated satisfactory distributions of the number of detected genes per cell (nFeature_RNA), total transcript counts per cell (nCount_RNA), and mitochondrial transcript percentages across both groups (Figure 2A). Additional quality control diagnostics, including relationships among nCount_RNA, nFeature_RNA, and mitochondrial transcript content, as well as principal-component selection metrics, are presented in Figure S1. Visualization of cells according to sample identity further confirmed the distribution of cells across the integrated single-cell landscape (Figure S2).

Dimensionality reduction and unsupervised clustering identified 21 transcriptionally distinct cell clusters within the integrated dataset (Figure 2B). UMAP visualization demonstrated broad cellular heterogeneity across ligamentum flavum tissues and revealed distinct distributions of LDH_NLF and LSS_HLF cells within the transcriptional landscape (Figure 2C).

Cluster-specific marker analysis identified representative transcriptional programs associated with stromal, ECM-producing, contractile, endothelial, immune, granulocytic, lymphoid, and erythroid cell populations (Table S2).

These findings demonstrate substantial cellular heterogeneity within the analyzed ligamentum flavum tissues and provide a framework for subsequent cell-type annotation and remodeling-associated transcriptomic analyses.

### 3.2. Cellular Annotation Identifies Fibroblast, Myofibroblast, and Endothelial Populations

**Figure 3:**
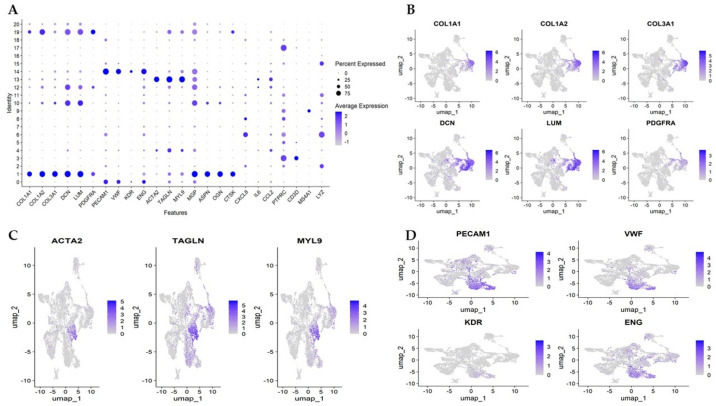
Marker-based annotation of major cellular populations in GSE294458. (**A**) Dot plot showing the expression of selected canonical marker genes across Seurat clusters. Dot size represents the percentage of cells expressing each marker, and color intensity represents scaled average expression. (**B**) Feature plots showing the UMAP distribution of fibroblast- and ECM-associated markers, including COL1A1, COL1A2, COL3A1, DCN, LUM, and PDGFRA. (**C**) Feature plots showing the expression of contractile and myofibroblast-associated markers, including ACTA2, TAGLN, and MYL9. (**D**) Feature plots showing the expression of endothelial-associated markers, including PECAM1, VWF, KDR, and ENG.

To define the major cellular populations contributing to ligamentum flavum remodeling, marker-based annotation was performed using cluster-specific transcriptional signatures identified in the quality-filtered GSE294458 dataset. Representative marker genes for each cluster are summarized in Tables S2 and S3. Dot-plot visualization of canonical markers demonstrated distinct transcriptional programs across the 21 Seurat clusters (Figure 3A). Several clusters exhibited strong expression of ECM-associated genes, including COL1A1, COL1A2, COL3A1, DCN, LUM, and PDGFRA, consistent with fibroblast-like stromal populations. Feature-plot analysis further confirmed the spatial localization of these markers within discrete regions of the UMAP landscape (Figure 3B).

A separate contractile cell population was identified by prominent expression of ACTA2, TAGLN, and MYL9, supporting the presence of myofibroblast-like cells within the hypertrophic ligamentum flavum microenvironment (Figure 3C). In addition, endothelial-associated clusters demonstrated a characteristic expression of PECAM1, VWF, KDR, and ENG, consistent with vascular endothelial cell populations (Figure 3D).

These marker-based annotations indicate that the ligamentum flavum cellular landscape contains fibroblast, myofibroblast, endothelial, and immune cell populations, thereby providing a cellular framework for subsequent analyses of fibrosis- and immune-associated remodeling programs.

### 3.3. Cellular Composition Differences Between LDH_NLF and LSS_HLF Tissues

**Figure 4:**
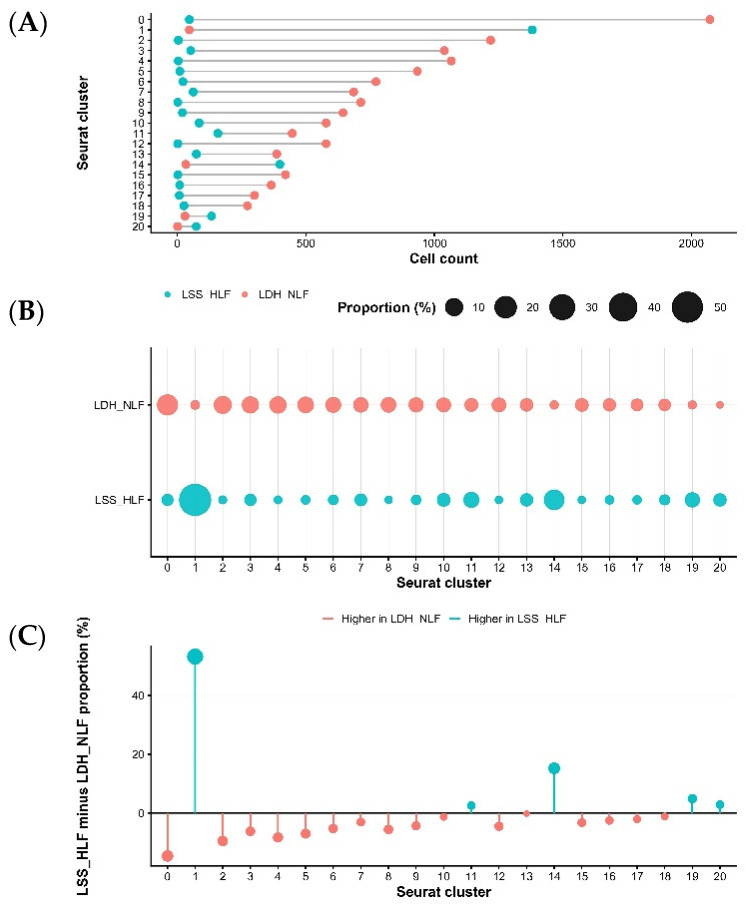
Cluster composition differences between LDH_NLF and LSS_HLF samples. (**A**) Dot plot showing the absolute number of cells assigned to each Seurat cluster in LDH_NLF and LSS_HLF samples. Each point represents the cell count for one group within a given cluster, and connecting lines indicate the difference between groups. (**B**) Bubble plot showing the relative contribution of each Seurat cluster within each group. Bubble size represents the proportion of cells assigned to each cluster within LDH_NLF or LSS_HLF samples. (**C**) Diverging lollipop plot showing the difference in cluster proportions between LSS_HLF and LDH_NLF samples. Positive values indicate clusters proportionally enriched in LSS_HLF, whereas negative values indicate clusters proportionally enriched in LDH_NLF. Cluster proportions were calculated after quality filtering.

Cluster composition analysis was performed to compare the distribution of Seurat clusters between LDH_NLF and LSS_HLF samples. Absolute cell counts demonstrated marked differences in the contribution of individual clusters between the two tissue groups (Figure 4A). In particular, cluster 1 represented the dominant cellular population in LSS_HLF, whereas several clusters that were more broadly represented in LDH_NLF contributed only minimally to the LSS_HLF sample.

Relative composition analysis further highlighted sample-specific differences in cluster abundance (Figure 4B). Cluster-wise proportions showed that cluster 1 accounted for more than half of LSS_HLF cells, whereas cluster 14 and cluster 19 also contributed more prominently to LSS_HLF than to LDH_NLF. In contrast, several clusters, including clusters 0, 2, 3, 4, 5, 6, 8, 9, 12, and 15, were proportionally more represented in LDH_NLF. Detailed cluster-wise cell counts and within-group proportions are provided in Table S4.

A diverging lollipop plot was used to summarize proportional differences between LSS_HLF and LDH_NLF across all clusters (Figure 4C).

This visualization confirmed that the largest positive proportional shift in LSS_HLF was observed for cluster 1, whereas multiple clusters showed relative enrichment in LDH_NLF. These composition differences suggest distinct cellular organization patterns between the LDH_NLF and LSS_HLF samples. Given the availability of one sample per group in GSE294458, these composition patterns should be interpreted as sample-associated observations rather than definitive disease-wide estimates, and they require confirmation in larger cohorts.

### 3.4. Bulk Differential Gene-Expression Analysis Identifies a Focused LFH-Associated Transcriptomic Signature

To characterize transcriptomic alterations associated with LFH, differential gene-expression analysis was performed using the GSE113212 dataset, which compared Old ligamentum flavum samples with Young controls. Principal component analysis (PCA) of the normalized expression matrix demonstrated separation tendencies between the two groups, with PC1 and PC2 explaining 29.5% and 21.1% of the total variance, respectively (Figure S3). Differential expression analysis was conducted using the limma framework, and significant DEGs were defined using a Benjamini–Hochberg-adjusted *p* value < 0.05 and an absolute log2 fold-change > 1. Among 35,789 analyzed probes/transcripts, nine significant DEGs were identified, comprising seven upregulated and two downregulated genes in Old samples relative to Young controls (Table 1; Table S5). The upregulated genes were *ULBP1*, *CHRM3*, *BMPR1B*, *CHST10*, *TGFB1*, *COL13A1*, and *MDM2*, whereas *MYADML* and *LOC283404* were downregulated (Table 1; Tables S6 and S7). It should be noted that the 651 inverse-regulated core genes reported in Section 3.9 were not obtained by expanding this focused nine-gene DEG signature. Direct comparison confirmed that none of the nine primary DEGs were present among the 651 inverse-regulated core genes; the entire core-gene set was instead derived by intersecting the predicted and validated targets of the dysregulated OLF-derived miRNAs with the directional LFH-associated expression profiles of GSE113212 (Section 2.7). The two gene sets therefore reflect distinct selection strategies—a stringent, threshold-based differential-expression signature versus a broader miRNA-target-informed inverse-regulatory layer—and address complementary analytical questions. Reassuringly, despite this methodological independence, the 651-gene set remained biologically coherent with the fibrotic–immune remodeling context of LFH, encompassing extracellular-matrix and remodeling-associated genes such as multiple collagens, *MMP9*, *TIMP1*, *SPARC*, fibrillins, ADAM/ADAMTS proteinases, integrins, and laminins.

**Figure 5:**
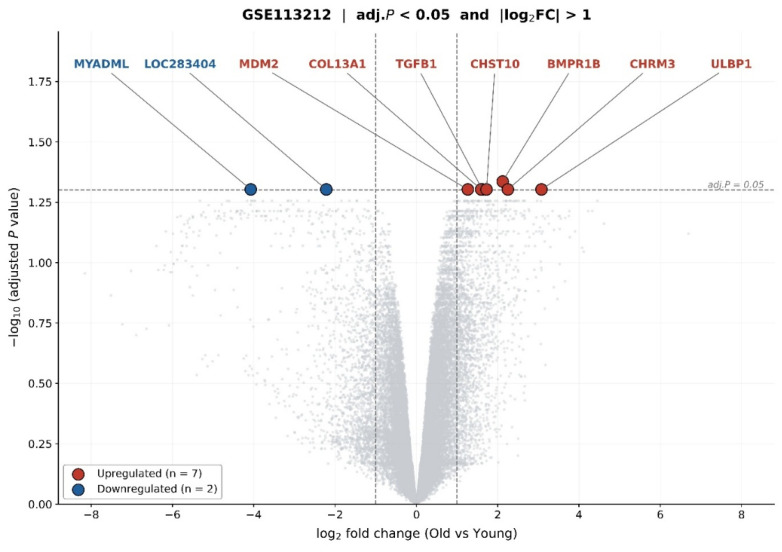
Differential gene-expression landscape of GSE113212. Volcano plot showing differential gene-expression results for the comparison of Old and Young ligamentum flavum samples. Each point represents a single probe/transcript. The x-axis indicates log2 fold change (Old vs. Young), and the y-axis indicates −log10 adjusted *p* value. Vertical dashed lines denote the |log2FC| > 1 threshold, and the horizontal dashed line denotes the adjusted *p* = 0.05 significance threshold. Red points indicate significantly upregulated genes, blue points indicate significantly downregulated genes, and gray points indicate probes/transcripts that did not meet the predefined thresholds. The analysis identified nine significant DEGs, comprising seven upregulated and two downregulated genes.

Visualization of the differential-expression landscape using a volcano plot confirmed that these genes represented a discrete subset of the transcriptome meeting the predefined statistical and fold-change thresholds (Figure 5).

Supplementary volcano and heatmap visualizations further demonstrated distinct expression patterns of the identified DEGs across Young and Old samples (Figure S4).

Several of the upregulated genes were associated with biological processes relevant to LFH pathophysiology. In particular, *TGFB1*, *COL13A1*, and *CHST10* are linked to extracellular-matrix remodeling, fibrotic signaling, and GAG-associated biology, whereas ULBP1 may reflect immune-associated transcriptional alterations. These findings identified a focused LFH-associated DEG signature that provided the bulk-transcriptomic foundation for subsequent miRNA integration and downstream regulatory-network analyses.

### 3.5. Exploratory Single-Cell Gene-Expression Signatures in GSE294458

To further characterize transcriptional remodeling at single-cell resolution, an exploratory all-cell differential-expression comparison was performed between LSS_HLF and LDH_NLF cells in GSE294458. This analysis identified 5781 significant DEGs, comprising 2753 genes with higher expression and 3028 genes with lower expression in LSS_HLF cells relative to LDH_NLF cells (Table S8). To facilitate biological interpretation, representative genes were selected from the significant DEG set to summarize major transcriptional programs associated with the two samples (Figure S5). Genes with higher expression in LSS_HLF cells included extracellular-matrix, stromal, and fibrosis-associated transcripts such as ASPN, LRRC15, MXRA5, GDF10, CCN5, FNDC1, OGN, and FIBIN (Figure S5; Table S9). In contrast, genes with lower expression in LSS_HLF cells included immune-, inflammatory-, granulocytic-, and myeloid-associated transcripts, including *IL1R2*, *S100A12*, *AQP9*, *CSF3R*, *S100A8*, *S100A9*, *ADAMTS4*, and *MNDA* (Figure S5; Table S10).

These transcriptional patterns were consistent with enhanced stromal/extracellular-matrix-associated remodeling accompanied by lower expression of several immune- and myeloid-associated transcriptional signatures in the LSS_HLF sample. Because GSE294458 contains a single specimen per study group, these findings were interpreted as exploratory and sample-associated observations rather than population-level estimates.

### 3.6. Supplementary LFH-Only Single-Cell Marker Assessment Using GSE267819

An independent LFH-only single-cell dataset, GSE267819, was evaluated as a supplementary marker-level resource. After quality filtering, 18,470 cells were retained, including 1914 LFH1 cells, 13,094 LFH2 cells, and 3462 LFH3 cells (Table S11). Because LFH2 contributed substantially more cells than LFH1 and LFH3, a balanced object was generated by downsampling each sample to 1914 cells, yielding 5742 cells for sample-balanced visualization and marker assessment (Tables S12 and S13). QC plots and balanced UMAP visualizations documented the distribution of LFH1, LFH2, and LFH3 cells across 11 Seurat clusters in the balanced object (Figure S6; Tables S14 and S15).

Canonical marker assessment showed that all 31 selected marker genes were detected in the balanced object, covering fibroblast/ECM, contractile, endothelial, pericyte/smooth-muscle, immune/myeloid, lymphoid, mast-cell-associated, and Schwann/neural-associated marker categories (Table S16). DotPlot and FeaturePlot visualization supported the presence of stromal/matrix-associated, contractile, endothelial, immune/myeloid, and mast-cell-related transcriptional patterns within LFH samples (Figure S7; Table S17). Because GSE267819 lacked a non-LFH control group, these results were interpreted only as supportive marker-level evidence and were not used for LFH-versus-control differential expression, integration, enrichment, network, diagnostic, or mechanistic analyses.

### 3.7. Supportive Transcript-Level Remodeling Analysis Using GSE106253

To determine whether ligament ossification/remodeling is accompanied by broader transcript-level alterations, GSE106253 was analyzed as an independent supportive dataset comparing OLF tissues with normal ligamentum flavum tissues. Differential-expression analysis identified 894 significant protein-coding transcript-level features and 1300 significant noncoding transcript-level features in OLF tissues compared with normal ligamentum flavum tissues. These transcript-level alterations are summarized by PCA, volcano-plot, and heatmap visualizations in Figure S8.

Because GSE106253 represents OLF rather than LFH, these findings were interpreted as supportive evidence of ligament ossification/remodeling-associated transcriptional remodeling and were not used as direct LFH discovery evidence, miRNA–mRNA integration input, hub gene prioritization input, diagnostic-modeling input, or mechanistic proof.

### 3.8. Supportive OLF miRNA Remodeling Analysis Using GSE106256

To provide a complementary regulatory layer for downstream integration, the independent OLF miRNA-sequencing dataset GSE106256 was analyzed. This dataset included four OLF and four normal ligamentum flavum samples. Following expression filtering, 605 mature miRNAs were retained for differential-expression analysis. A total of 33 DEMs were identified using FDR < 0.05 and |log2FC| > 1, including 20 upregulated and 13 downregulated miRNAs in OLF tissues (Figure S9; Table S18).

Among the significantly upregulated DEMs were miR-181a-5p, miR-653-5p, miR-642a-5p, miR-181a-3p, and miR-708-5p, whereas miR-381-3p, miR-379-5p, miR-337-5p, miR-215-5p, and miR-136-5p were among the downregulated DEMs (Table S18). Because GSE106256 represents OLF rather than LFH, these miRNAs were interpreted as a complementary ligament-remodeling regulatory resource rather than LFH-specific miRNA events. These DEMs were subsequently incorporated into inverse miRNA–mRNA integration analyses.

### 3.9. Identification of Inverse-Regulated miRNA–mRNA Core Genes

LFH-associated mRNA expression profiles from GSE113212 were integrated with predicted and/or validated target genes of dysregulated miRNAs identified in GSE106256. Directional filtering was subsequently applied to retain inverse miRNA–mRNA relationships, generating a regulatory framework of candidate inverse-regulated genes potentially associated with ligamentum flavum remodeling. This integrative analysis identified 651 inverse-regulated core genes representing OLF-derived miRNA–target relationships filtered against LFH-associated mRNA expression patterns (Table S19). These genes were retained as cross-dataset links between OLF-derived miRNA target information and LFH-associated transcriptomic alterations.

The inverse-regulated core-gene set contained numerous genes involved in ECM organization, immune regulation, cell differentiation, signal transduction, and tissue-remodeling processes and was consistent with overlap between OLF-derived miRNA target information and LFH-associated fibrotic and immune transcriptional programs. Because these genes satisfied both the LFH-associated transcriptomic filter and the OLF-derived miRNA–target criterion, the resulting core-gene set was carried forward for functional-enrichment, PPI, hub gene, and LASSO/ROC analyses.

### 3.10. Functional Enrichment Analysis of the Inverse-Regulated Core Genes

**Figure 6:**
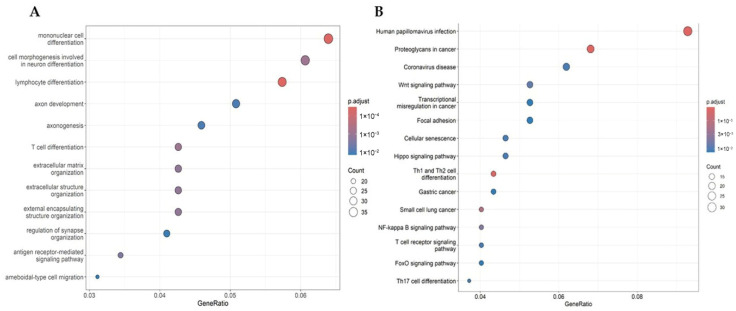
Functional enrichment analysis of the 651 inverse-regulated core genes. (**A**) GO-BP enrichment dot plot showing significantly enriched biological processes. Dot size represents the number of genes associated with each term, and color indicates the adjusted *p* value. Enriched GO-BP terms included mononuclear cell differentiation, lymphocyte differentiation, T cell differentiation, antigen receptor-mediated signaling pathway, ECM organization, extracellular structure organization, external encapsulating structure organization, axonogenesis, axon development, and regulation of synapse organization. (**B**) KEGG enrichment dot plot showing significantly enriched pathways. Dot size represents the number of mapped genes, and color indicates the adjusted *p* value. Enriched KEGG pathways included proteoglycans in cancer, Wnt signaling pathway, focal adhesion, Hippo signaling pathway, cellular senescence, NF-kappa B signaling pathway, T cell receptor signaling pathway, Th1 and Th2 cell differentiation, Th17 cell differentiation, and FoxO signaling pathway.

Functional enrichment analysis was performed to characterize the biological processes and pathways represented by the 651 inverse-regulated core genes. GO-BP analysis identified 44 significantly enriched biological processes (Table S20). The enriched terms included mononuclear cell differentiation, lymphocyte differentiation, T cell differentiation, antigen receptor-mediated signaling pathway, ECM organization, extracellular structure organization, external encapsulating structure organization, axonogenesis, axon development, and regulation of synapse organization (Figure 6A). These findings indicate that the inverse-regulated gene set is associated with both immune cell differentiation and tissue-structure remodeling processes.

KEGG analysis identified 42 significantly enriched pathways (Table S21). The enriched pathways included proteoglycans in cancer, Wnt signaling pathway, focal adhesion, Hippo signaling pathway, cellular senescence, NF-kappa B signaling pathway, T cell receptor signaling pathway, Th1 and Th2 cell differentiation, Th17 cell differentiation, and FoxO signaling pathway (Figure 6B).

Reactome enrichment, obtained from the STRING functional enrichment output, identified 12 enriched pathways (Figure S10; Table S22). Enriched Reactome terms included ECM organization, nervous system development, axon guidance, developmental biology, ECM degradation, NCAM1 interactions, NCAM signaling for neurite outgrowth, ECM proteoglycans, signal transduction, and collagen biosynthesis and modifying enzymes. The combined GO-BP, KEGG, and Reactome results support enrichment of the inverse-regulated core-gene set for ECM/proteoglycan remodeling, immune cell differentiation, inflammatory signaling, and developmental-remodeling programs in LFH.

### 3.11. PPI Network Construction of the Inverse-Regulated Core Genes

**Figure 7:**
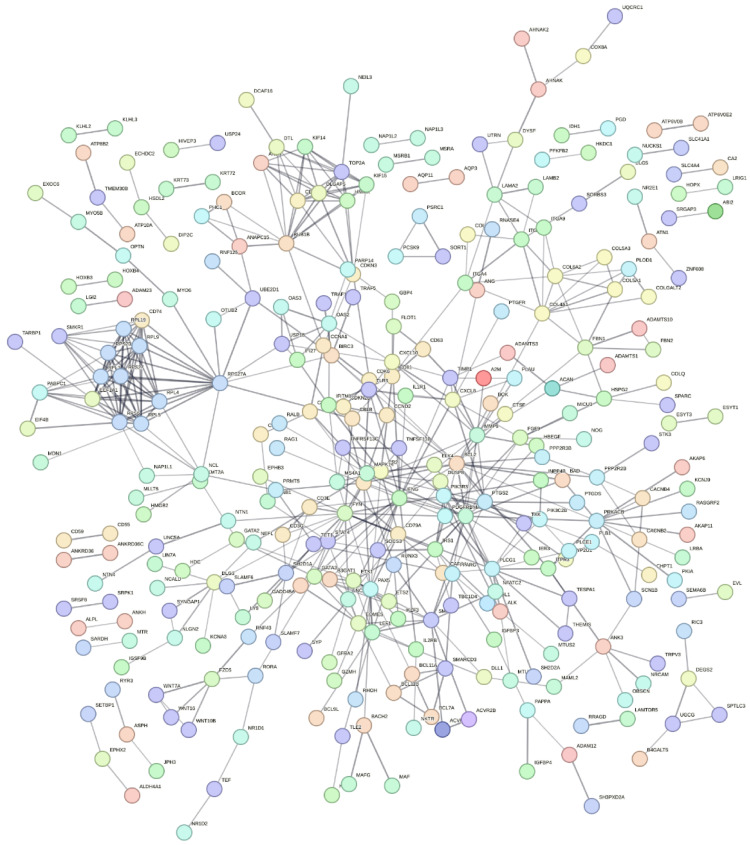
PPI network of inverse-regulated core genes. The 651 inverse-regulated core genes were submitted to STRING, and 650 genes were recognized as network nodes. The network was constructed using a high-confidence interaction threshold, and disconnected nodes were hidden during visualization. The resulting network contained 650 nodes and 547 edges, with an average node degree of 1.68, an average clustering coefficient of 0.319, and a PPI enrichment *p* value of 2.04 × 10^−14^.

To evaluate functional connectivity among the inverse-regulated core genes, the 651-gene set was submitted to STRING for PPI network construction. STRING recognized 650 genes as network nodes. After applying a high-confidence interaction threshold and hiding disconnected nodes for visualization, the resulting PPI network contained 650 nodes and 547 edges, with an average node degree of 1.68, an average clustering coefficient of 0.319, and a PPI enrichment *p* value of 2.04 × 10^−14^ (Figure 7).

The significant PPI enrichment indicated that the inverse-regulated core genes were more functionally connected than expected by chance. Therefore, the STRING-derived PPI network was used as the basis for subsequent Cytoscape-based hub gene prioritization.

### 3.12. CytoHubba-Based Hub Gene Prioritization

**Figure 8:**
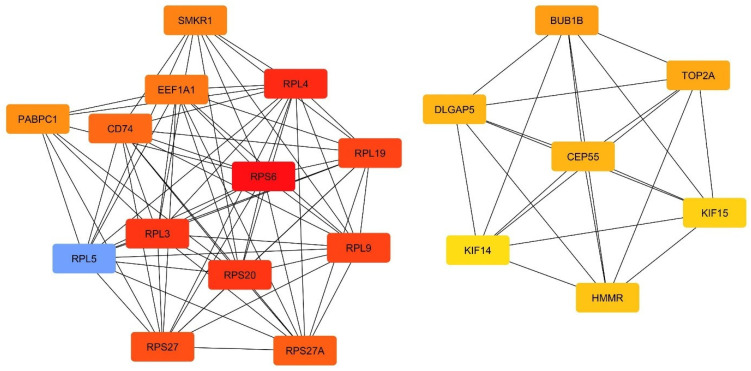
CytoHubba-derived hub gene network from the PPI network. The STRING-derived PPI network of inverse-regulated core genes was analyzed using the cytoHubba plugin in Cytoscape. The top 20 hub genes identified by maximal clique centrality (MCC) are shown. These hub genes were subsequently used for miRNA–hub gene regulatory-network construction and downstream prioritization analyses.

To identify key regulatory genes within the STRING-derived PPI network, hub gene analysis was performed using the cytoHubba plugin in Cytoscape. The maximal clique centrality (MCC) algorithm was used as the primary ranking method, while degree, closeness, betweenness, and edge-percolated component (EPC) metrics were evaluated as supportive topological measures. Evaluation of supportive topological metrics demonstrated that several MCC-prioritized genes also exhibited high network connectivity. Among the hub genes, *RPS27A* showed the highest degree score (17), followed by *RPS6* (15) and *RPL5* (14). *RPS27A* also exhibited the highest closeness and betweenness centrality values, indicating a potentially important role in maintaining network connectivity and information flow. Additional highly connected nodes included *RPL4*, *BUB1B, RPL3*, and *RPS20* (Table 2). The MCC-prioritized hub gene set consisted predominantly of ribosomal and translation-associated genes, including *RPS6*, *RPL5*, *RPL4*, *RPS20*, *RPL3*, *RPL9*, *RPL19*, *RPS27*, *RPS27A*, *EEF1A1*, *SMKR1*, and *PABPC1*, together with additional genes implicated in antigen presentation (CD74), cell-cycle regulation (*BUB1B*, *TOP2A*, *CEP55*, *KIF14*, *KIF15*, and *DLGAP5*), and microenvironmental remodeling (*HMMR*) (Figure 8; Table 3).

MCC ranking identified *RPS6*, *RPL5*, *RPL4*, *RPS20*, and *RPL3* as the highest-priority hub genes, with MCC scores ranging from 771,120 to 771,130. EPC analysis yielded broadly concordant results, with *RPS27A*, *RPS6*, *RPL5*, *RPS20*, and *RPL3* among the highest-scoring nodes, supporting the robustness of hub gene prioritization across multiple network-centrality algorithms (Table 3).

Overall, cytoHubba analysis highlighted a compact hub gene module dominated by ribosomal/translation-related genes together with cell-cycle and regulatory factors, suggesting that protein-synthesis machinery and proliferative-remodeling programs may represent candidate components of the integrated miRNA–mRNA regulatory landscape in LFH.

### 3.13. miRNA–Hub Gene Regulatory Network

**Figure 9:**
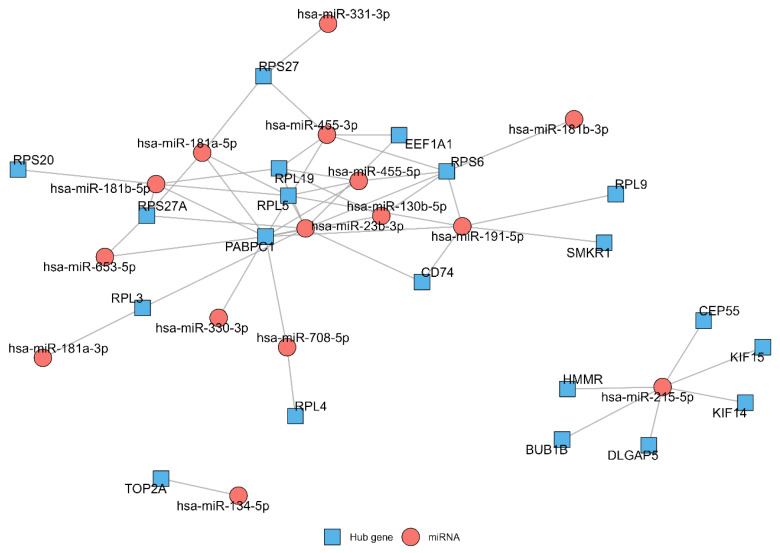
miRNA–hub gene regulatory network in LFH. Integrated miRNA–mRNA analysis identified candidate regulatory interactions between dysregulated miRNAs and hub genes derived from the STRING PPI network. Red circular nodes represent dysregulated miRNAs, whereas blue square nodes represent hub genes. Edges indicate database-supported candidate miRNA–target interactions. Highly connected candidate miRNAs, including hsa-miR-23b-3p, hsa-miR-191-5p, hsa-miR-215-5p, and hsa-miR-181b-5p, targeted multiple hub genes within the integrated regulatory network.

To further refine the integrated regulatory network toward topologically central remodeling-associated genes, the 20 CytoHubba-prioritized hub genes were intersected with the inverse miRNA–mRNA regulatory network. This analysis identified a core miRNA–hub gene network comprising 50 inverse miRNA–mRNA regulatory interactions involving 20 hub genes and 15 dysregulated miRNAs (Figure 9; Tables S23 and S24).

The retained interactions fulfilled both predefined criteria: inverse regulation between the dysregulated miRNA and its predicted or validated target gene, and inclusion of the target gene among the PPI-derived hub genes.

The network highlighted several miRNAs with multiple hub gene connections, including hsa-miR-23b-3p, hsa-miR-191-5p, hsa-miR-215-5p, and hsa-miR-181b-5p. These miRNAs were linked to hub genes representing ribosomal/translation-associated nodes, antigen-presentation-related genes, and remodeling-associated targets. Because the network was derived from integrated transcriptomic and database-based miRNA–target information, these miRNA–hub gene relationships were interpreted as candidate regulatory interactions rather than experimentally validated regulatory events.

### 3.14. LASSO-Based Candidate Feature Prioritization and Apparent Discovery-Cohort Discrimination

**Figure 10:**
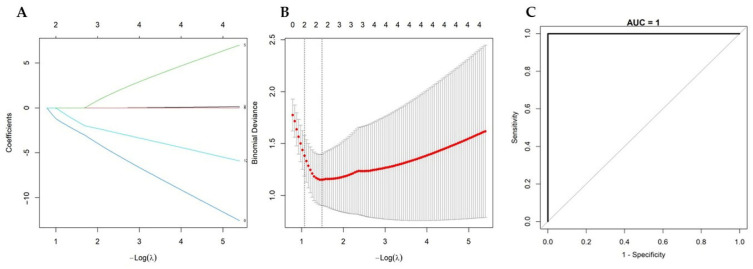
LASSO feature selection and apparent discovery-cohort discrimination of candidate hub features. (**A**) LASSO coefficient profiles for cytoHubba-prioritized hub genes. (**B**) Cross-validation curve used for lambda selection. (**C**) ROC curve of the two-gene PABPC1–RPL4 candidate-feature score in the GSE113212 discovery cohort. The AUC of 1.00 represents apparent performance within the small GSE113212 discovery cohort and does not constitute external diagnostic validation. In panel (**A**), the differently colored lines represent the coefficient trajectories of individual candidate hub genes across the regularization path. In panel (**B**), the red points and curve indicate the mean cross-validated error, the gray vertical bars represent the corresponding standard errors, and the vertical dashed lines indicate the cross-validation-based criteria used for lambda selection. In panel (**C**), the black line represents the ROC curve, whereas the light-gray diagonal line indicates chance-level discrimination.

LASSO regression prioritized *PABPC1* and *RPL4* as exploratory candidate hub features among the CytoHubba-prioritized hub gene set (Figure 10A,B). Both genes showed reduced expression in the Old/LFH group relative to Young/control samples. The two-gene *PABPC1*–*RPL4* candidate-feature score showed apparent discovery-cohort discrimination in GSE113212, with an AUC of 1.000 and sensitivity and specificity values of 1.000 (Figure 10C). Given the absence of an independent diagnostic-validation cohort, this finding was interpreted as an exploratory discovery-cohort result rather than validated diagnostic performance.

To provide additional statistical context, supplementary uncertainty, internal-stability, and separation-aware sensitivity analyses were performed for the exploratory PABPC1–RPL4 candidate-feature score (Table S25; Figure S11). In GSE113212, this score showed apparent discovery-cohort discrimination between Old/LFH and Young/control samples. The cohort included eight samples, comprising four Old/LFH and four Young/control samples. The direction-aware score yielded an AUC of 1.000. At the Youden-optimal cutoff of 0.145227, sensitivity and specificity were both 1.000, with exact 95% confidence intervals of 0.398–1.000 for each measure. The DeLong AUC confidence interval was 1.000–1.000, which in this setting reflected complete separation within a small discovery cohort rather than independent diagnostic validation.

Internal-stability analyses of the predefined *PABPC1*–*RPL4* score showed the same apparent class separation within this dataset, with an LOOCV AUC of 1.000 and a mean AUC of 1.000 across all 24 balanced stratified four-fold allocations; the corresponding standard deviation, minimum, and maximum AUC values were 0.000, 1.000, and 1.000, respectively. Consistent with this behavior, the standard two-gene logistic model generated the warning glm.fit: fitted probabilities numerically 0 or 1 occurred and were therefore not interpreted as a stable diagnostic model.

As a separation-aware sensitivity analysis, Firth bias-reduced logistic modeling converged and yielded finite fitted probabilities while preserving the same class ordering; the corresponding Firth-probability AUC was 1.000, with a Youden cutoff of 0.5219. A class-stratified case-resampling bootstrap remained degenerate at 1.000–1.000 under complete separation and was therefore not interpreted as formal uncertainty evidence. In complementary perturbation-based sensitivity analyses, the mean AUC remained 1.000 at 10% feature-level noise, was 0.990 with a 2.5th–97.5th percentile range of 0.938–1.000 at 50% noise, and was 0.932 with a 2.5th–97.5th percentile range of 0.688–1.000 at 100% noise. Taken together, these analyses contextualize the uncertainty and internal stability of the *PABPC1*–*RPL4* score and support its interpretation as a discovery-cohort finding rather than independent diagnostic validation.

### 3.15. Cross-Cohort Comparison of LASSO-Selected Genes

**Figure 11:**
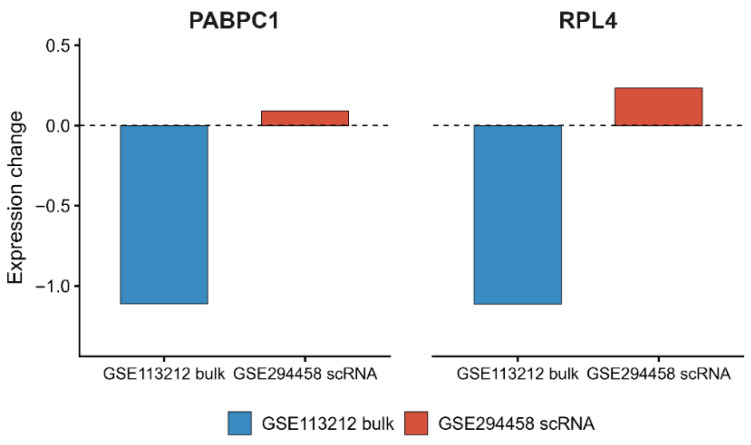
Cross-cohort comparison of LASSO-selected genes across bulk and single-cell ligamentum flavum datasets. Expression changes of the two LASSO-selected genes identified in GSE113212 were compared with mean expression differences observed in the independent single-cell dataset GSE294458. In GSE113212, PABPC1 and RPL4 showed reduced expression in the hypertrophic/elderly ligamentum flavum group, whereas both genes showed higher mean expression in LSS_HLF cells than in LDH_NLF cells in GSE294458. The horizontal dotted line denotes zero expression change; values below and above this reference line indicate decreased and increased expression, respectively.

To assess whether the LASSO-selected genes exhibited comparable expression patterns across independent transcriptomic datasets, expression changes of *PABPC1* and *RPL4* identified in GSE113212 were compared with expression differences observed in the independent single-cell dataset GSE294458 (Figure 11; Table S26).

In the bulk transcriptomic dataset, both genes were downregulated in the elderly LFH-associated group (*PABPC1* logFC = −1.1115; *RPL4* logFC = −1.1138). In contrast, both genes showed higher mean expression in LSS_HLF cells than in LDH_NLF cells in GSE294458 (mean expression change = 0.0919 for *PABPC1* and 0.2335 for *RPL4*). Consequently, neither gene demonstrated directional concordance across the two datasets (Table S26). These findings indicate that the selected genes were detectable across transcriptomic layers but exhibited discordant expression patterns between bulk and single-cell analyses.

### 3.16. Fibrotic and Immune Remodeling Signatures in the Independent Single-Cell Dataset

**Figure 12:**
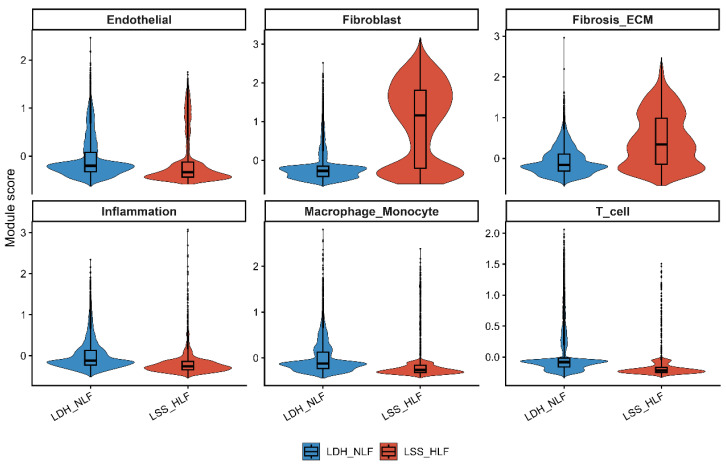
Fibrotic and immune remodeling signatures in GSE294458. Single-cell module-score analysis was performed to evaluate fibrosis-, ECM-, endothelial-, fibroblast-, inflammatory-, macrophage/monocyte-, and T cell-associated transcriptional programs in LSS_HLF and LDH_NLF cells. Violin plots show the distribution of single-cell module scores, with embedded boxplots representing the median and interquartile range. Statistical comparisons were performed using Wilcoxon rank-sum tests with Benjamini–Hochberg false-discovery-rate correction. Abbreviations: LSS_HLF, lumbar spinal stenosis-associated hypertrophic ligamentum flavum; LDH_NLF, lumbar disc herniation-associated non-hypertrophic ligamentum flavum; ECM, extracellular matrix. Because GSE294458 consisted of a single specimen per condition, these comparisons should be interpreted as exploratory, sample-associated observations rather than population-level or statistically generalizable findings, notwithstanding the use of formal significance testing.

To further characterize transcriptional remodeling patterns in an independent dataset, module-score analysis was performed in GSE294458 using predefined fibrosis-, fibroblast-, endothelial-, inflammation-, macrophage/monocyte-, and T cell-associated gene signatures (Figure 12; Tables S27–S29).

Within the two specimens represented in GSE294458, LSS_HLF cells showed higher Fibroblast and Fibrosis_ECM signature scores than LDH_NLF cells, consistent with a sample-associated shift toward fibrocellular and ECM-related transcriptional programs.

In contrast, Endothelial, Inflammation, Macrophage_Monocyte, and T_cell signature scores were lower in LSS_HLF cells than in LDH_NLF controls (Figure 12; Tables S28 and S29). Cell-level summary statistics within the two specimens were consistent with relatively stronger fibroblast-associated and ECM-related transcriptional signals in LSS_HLF cells and lower immune-associated signals than in LDH_NLF cells. Median Fibroblast scores increased from −0.272 in LDH_NLF cells to 1.160 in LSS_HLF cells, whereas median Fibrosis_ECM scores increased from −0.159 to 0.343. Conversely, median Inflammation, Macrophage_Monocyte, T_cell, and Endothelial scores were reduced in LSS_HLF cells (Table S28).

Within the two specimens represented in GSE294458, these findings were consistent with relatively stronger fibrotic and fibroblast-associated transcriptional signals in LSS_HLF cells. Because GSE294458 consisted of a single sample per group and the analysis was performed at the single-cell level, these observations should be interpreted as exploratory transcriptomic patterns rather than definitive evidence of altered cellular abundance or biological causality.

### 3.17. Network-Level miRNA Prioritization Based on Hub Gene Targeting Capacity

**Figure 13:**
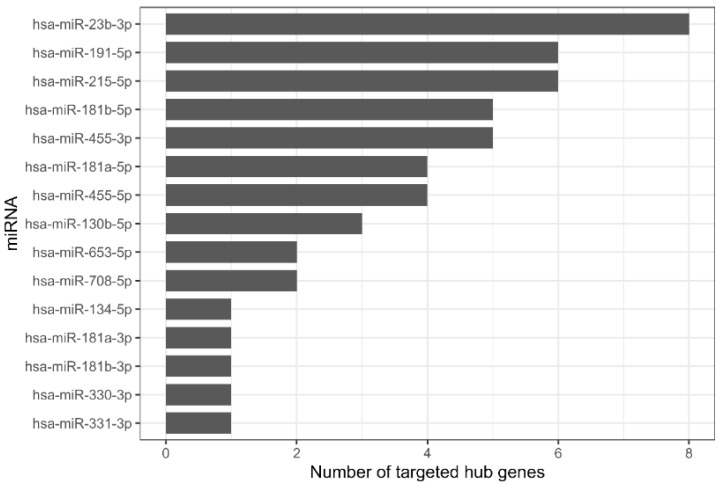
Network-level miRNA prioritization based on hub gene targeting capacity. Dysregulated miRNAs were ranked according to the number of unique hub genes targeted within the integrated miRNA–hub gene regulatory network. The bar plot shows hub gene targeting breadth for each candidate miRNA. hsa-miR-23b-3p exhibited the broadest regulatory coverage, followed by hsa-miR-191-5p, hsa-miR-215-5p, hsa-miR-181b-5p, and hsa-miR-455-3p. These miRNAs represent network-level regulatory candidates selected according to hub gene targeting capacity.

To identify high-priority regulatory miRNAs within the LFH-associated remodeling network, dysregulated miRNAs were ranked according to the number of CytoHubba-prioritized hub genes targeted within the integrated inverse miRNA–hub gene network (Figure 13; Table S30).

Among the identified candidates, hsa-miR-23b-3p exhibited the broadest regulatory coverage, targeting eight hub genes (*CD74*, *EEF1A1*, *PABPC1*, *RPL19*, *RPL3*, *RPL5*, *RPS27A*, and *RPS6*). hsa-miR-191-5p and hsa-miR-215-5p each targeted six hub genes, whereas hsa-miR-181b-5p and hsa-miR-455-3p targeted five hub genes. Additional candidate regulators included hsa-miR-181a-5p and hsa-miR-455-5p (four targets each), hsa-miR-130b-5p (three targets), and hsa-miR-653-5p and hsa-miR-708-5p (two targets each) (Table S30).

The highest-ranked miRNAs targeted multiple ribosomal hub genes together with *PABPC1* and *CD74*, linking them to the fibrosis-, extracellular-matrix-, proteoglycan/GAG-, and antigen-presentation-associated remodeling programs identified throughout the integrated analyses. These findings suggest that a relatively small subset of dysregulated miRNAs may exert broad regulatory influence across multiple network-prioritized genes involved in LFH-associated molecular remodeling.

Accordingly, hsa-miR-23b-3p, hsa-miR-191-5p, hsa-miR-215-5p, hsa-miR-181b-5p, and hsa-miR-455-3p were prioritized as the principal network-level regulatory candidates for downstream integrative interpretation. These candidates were selected on the basis of network connectivity and hub gene targeting breadth and should therefore be regarded as network-level candidate regulatory miRNAs rather than experimentally validated therapeutic targets. Thus, hub gene targeting breadth was used as a network-connectivity criterion to identify miRNAs with broad regulatory coverage, whereas subsequent prioritization in the proposed computational model gave precedence to miRNAs overlapping with the LASSO-selected candidate features *PABPC1* and *RPL4* and their remodeling-signature associations. Under this hierarchy, hsa-miR-23b-3p represented the broadest network-level regulator, whereas hsa-miR-708-5p was prioritized in the proposed computational model because it simultaneously targeted both *PABPC1* and *RPL4*. 

### 3.18. KEGG Gene Set Enrichment Analysis of Ranked Transcriptomic Signatures

**Figure 14:**
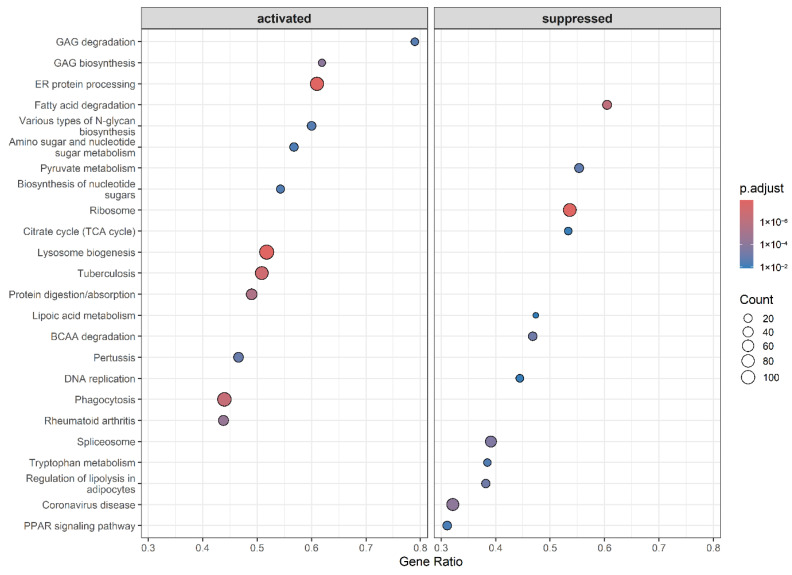
KEGG gene set enrichment analysis (GSEA) of ranked transcriptomic signatures in ligamentum flavum hypertrophy (LFH). Pre-ranked GSEA was performed using the complete GSE113212 transcriptomic dataset ranked according to moderated t-statistics derived from the comparison between hypertrophic ligamentum flavum and non-hypertrophic control ligamentum flavum. Significantly enriched KEGG pathways are displayed according to normalized enrichment score, gene ratio, pathway size, and adjusted *p* value. Positive normalized enrichment scores indicate pathways enriched in the LFH-associated group, whereas negative normalized enrichment scores indicate pathways enriched in the control group.

Pre-ranked GSEA was performed using the complete GSE113212 bulk transcriptomic profile to evaluate pathway-level remodeling beyond threshold-based DEG selection. KEGG GSEA identified both activated and suppressed pathway signatures in elderly LFH-associated samples (Figure 14; Table S31).

Pathways enriched in the LFH-associated group included GAG degradation, GAG biosynthesis, protein processing in the endoplasmic reticulum, lysosome biogenesis, phagocytosis, and protein digestion and absorption. In contrast, pathways enriched in the control group included ribosome, fatty-acid degradation, citrate cycle, pyruvate metabolism, tryptophan metabolism, regulation of lipolysis in adipocytes, and the PPAR signaling pathway (Figure 14; Table S31). These findings indicate a transcriptomic shift from metabolic and translational homeostasis toward ECM, GAG, lysosomal, and immune-associated remodeling programs in LFH.

### 3.19. GO Biological Process GSEA of Ranked Transcriptomic Signatures

**Figure 15:**
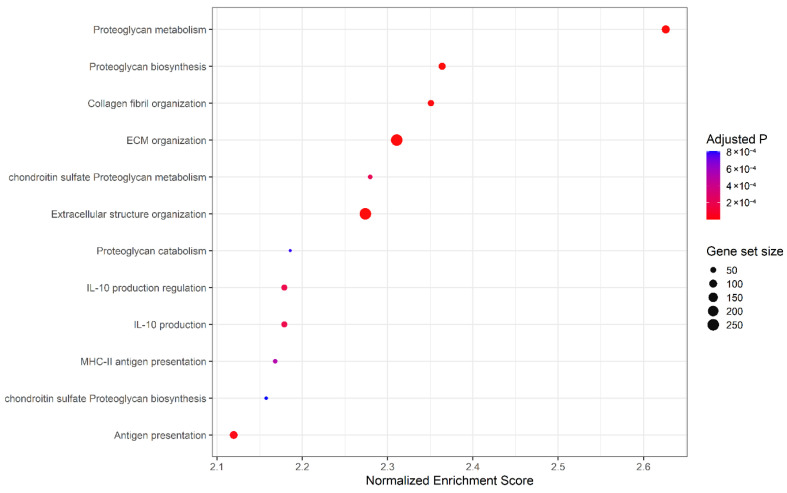
GO Biological Process gene set enrichment analysis highlights extracellular matrix, proteoglycan, and immune-remodeling programs in LFH. Gene set enrichment analysis (GSEA) was performed using the complete ranked transcriptomic profile of GSE113212, ordered by moderated t-statistics from the hypertrophic/Old versus non-hypertrophic/Young ligamentum flavum comparison. Selected significantly enriched GO Biological Process terms are shown to emphasize the dominant biological programs associated with LFH. Dot position represents the normalized enrichment score (NES), dot size indicates gene set size, and color denotes the Benjamini–Hochberg-adjusted *p* value. Positive NES values indicate enrichment in hypertrophic ligamentum flavum. The enriched processes were dominated by proteoglycan metabolism, proteoglycan biosynthesis, proteoglycan catabolism, chondroitin sulfate proteoglycan remodeling, collagen fibril organization, ECM organization, extracellular structure organization, antigen presentation, MHC-II antigen presentation, and IL-10-related immune regulation.

GO Biological Process (GO-BP) GSEA was performed using the complete ranked GSE113212 transcriptomic profile to further characterize biological programs associated with LFH-related remodeling (Figure 15; Table S32).

Enriched processes in the LFH-associated group were dominated by ECM organization, extracellular structure organization, collagen fibril organization, collagen metabolic process, and positive regulation of ECM organization. Prominent enrichment was also observed for proteoglycan- and GAG-related processes, including proteoglycan metabolism, proteoglycan biosynthesis and catabolism, chondroitin sulfate proteoglycan metabolism and biosynthesis, dermatan sulfate proteoglycan metabolism, heparan sulfate proteoglycan metabolism, and GAG biosynthetic and metabolic processes.

In parallel, multiple immune-associated processes were enriched, including antigen processing and presentation, MHC class I- and class II-related antigen presentation, MHC protein complex assembly, peptide antigen assembly, leukocyte migration, mononuclear-cell migration, macrophage migration, macrophage chemotaxis, granulocyte migration, and granulocyte chemotaxis. Additional enrichment was observed for interleukin-10 production and regulation of interleukin-10 production.

Overall, the results indicate that LFH-associated transcriptomic remodeling involves coordinated extracellular-matrix and proteoglycan turnover together with enhanced antigen-presentation and immune cell migration programs, supporting a fibrotic–immune remodeling phenotype.

### 3.20. Remodeling Signature Analysis and LASSO-Gene Associations in GSE113212

**Figure 16:**
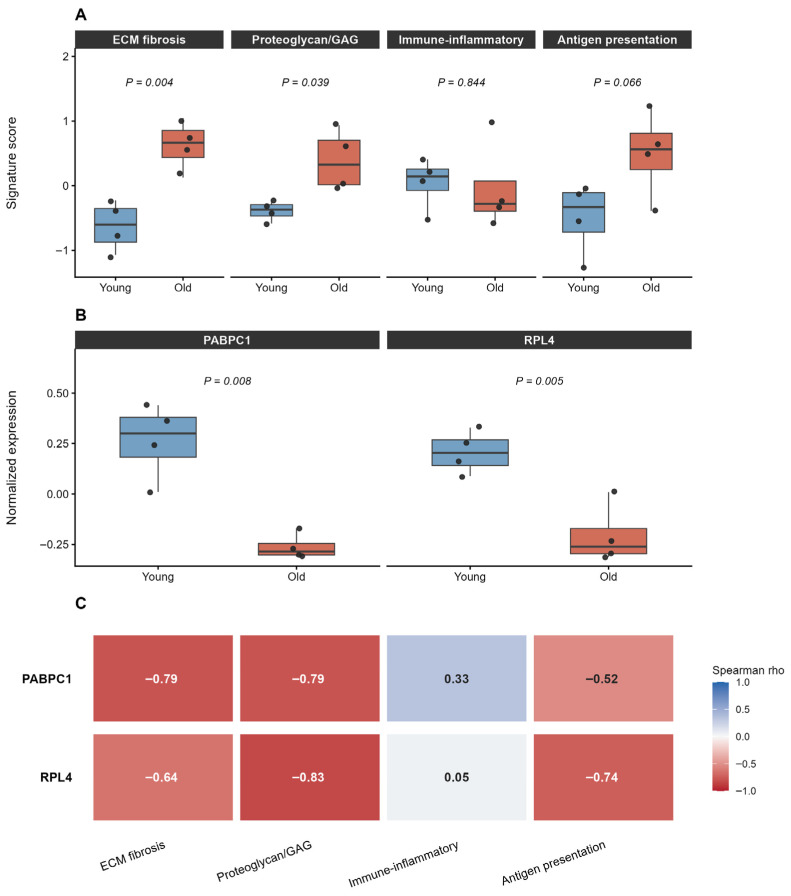
Integrated fibrosis–immune signature remodeling and LASSO-gene association in GSE113212. (**A**) Comparison of fibrosis-, proteoglycan/GAG-, immune-inflammatory-, and antigen-presentation-related signature scores between young and elderly ligamentum flavum samples. Signature scores were calculated as the mean of the gene-wise normalized expression values of all available genes within each predefined signature set. ECM fibrosis and proteoglycan/GAG signatures were significantly increased in elderly samples, whereas antigen-presentation activity showed a trend toward increase and immune-inflammatory signatures were not significantly different between groups. (**B**) Expression levels of the LASSO-selected genes *PABPC1* and *RPL4* in young and elderly samples. Both genes exhibited significantly reduced expression in the elderly group. (**C**) Spearman correlation heatmap showing associations between LASSO-selected genes and fibrosis–immune signature scores across all GSE113212 samples. Negative correlation coefficients indicate lower gene expression associated with higher signature activity. Correlation coefficients are displayed within each cell. Statistical comparisons in panels A and B were performed using two-sided Welch’s *t*-tests. Complete sample-level signature scores and correlation statistics are provided in Table S33A,B, and the complete gene lists defining each signature are provided in Table S33C. Abbreviations: ECM, extracellular matrix; GAG, glycosaminoglycan; LFH, ligamentum flavum hypertrophy.

To further evaluate the biological relevance of the LASSO-selected genes, fibrosis-, proteoglycan/GAG-, immune-inflammatory-, and antigen-presentation-related signature scores were calculated in the GSE113212 cohort. ECM fibrosis and proteoglycan/GAG signatures were significantly increased in the elderly LFH-associated group compared with young controls (*p* = 0.004 and *p* = 0.039, respectively), whereas antigen-presentation activity showed a trend toward increase (*p* = 0.066) and the immune-inflammatory signature did not differ significantly between groups (*p* = 0.844) (Figure 16A). Sample-level signature scores are provided in Table S33A.

Consistent with the LASSO model, *PABPC1* and *RPL4* both showed significantly reduced expression in the elderly LFH-associated group (*p* = 0.008 and *p* = 0.005, respectively) (Figure 16B). Spearman correlation analysis further showed that reduced *PABPC1* and *RPL4* expression was inversely associated with ECM fibrosis, proteoglycan/GAG, and antigen-presentation signature activity, whereas correlations with the immune-inflammatory signature were weak or absent (Figure 16C). Complete correlation coefficients and corresponding *p* values are provided in Table S33B.

Together, these findings link the LASSO-selected genes to LFH-associated fibrotic, proteoglycan, and antigen-presentation remodeling at the transcriptomic signature level. The complete gene lists defining each remodeling signature, together with a leave-one-gene-out sensitivity analysis of signature composition, are provided in Table S33C.

### 3.21. Integrated miRNA–Hub Gene–Signature Framework and Proposed Computational Model

To integrate the miRNA regulatory network with transcriptomic remodeling signatures, miRNAs were evaluated according to hub gene targeting breadth and links to the LASSO-selected genes *PABPC1* and *RPL4* (Table S34). Among the candidate miRNAs, hsa-miR-708-5p emerged as the highest-priority regulator because it simultaneously targeted both LASSO-selected genes, *PABPC1* and *RPL4*. Several additional dysregulated miRNAs, including hsa-miR-23b-3p, hsa-miR-191-5p, hsa-miR-181b-5p, hsa-miR-455-3p, hsa-miR-181a-5p, and hsa-miR-653-5p, were linked to *PABPC1*, suggesting a convergent regulatory architecture centered on this LASSO-selected hub gene (Table S34).

To further connect regulatory miRNAs with biologically relevant transcriptomic processes, inverse miRNA–hub gene interactions involving the LASSO-selected genes shown in Figure 16 were integrated with fibrosis-, proteoglycan/GAG-, and antigen-presentation-related signature analyses (Table S35). All identified interactions involved upregulated miRNAs paired with downregulated hub genes, consistent with inverse regulatory relationships. PABPC1 was linked to ECM fibrosis, proteoglycan/GAG remodeling, and antigen-presentation signatures through multiple dysregulated miRNAs, whereas hsa-miR-708-5p simultaneously targeted both *PABPC1* and *RPL4*. *RPL4* showed significant associations with proteoglycan/GAG remodeling and antigen-presentation activity. These findings suggest that dysregulated miRNAs may contribute to LFH-associated fibrotic–immune remodeling through coordinated suppression of key hub genes.

**Figure 17:**
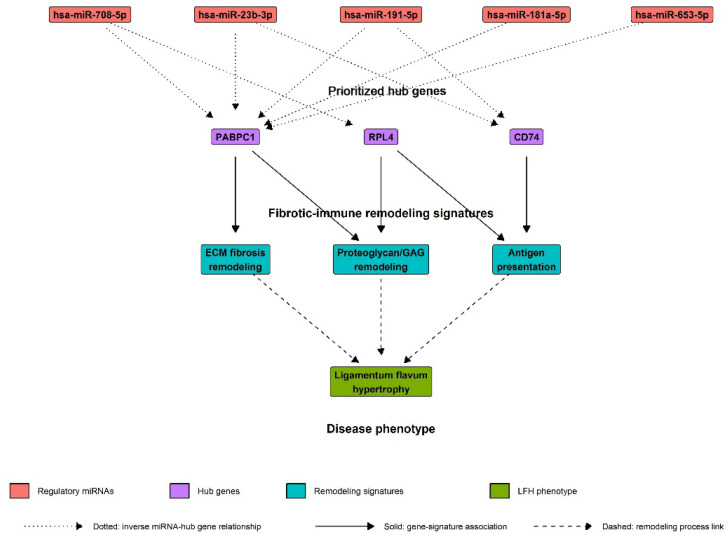
Proposed computational model linking prioritized candidate miRNA regulators, hub genes, and fibrotic–immune remodeling in LFH. Candidate regulatory miRNAs were prioritized by integrating miRNA–hub gene network connectivity with links to the LASSO-selected genes *PABPC1* and *RPL4*. Upregulated miRNAs were connected to hub genes through inverse regulatory relationships, whereas hub genes were linked to transcriptomic remodeling signatures associated with ECM fibrosis, proteoglycan/GAG remodeling, and antigen presentation. Dotted arrows indicate inverse miRNA–hub gene relationships, solid arrows indicate gene–signature associations, and dashed arrows indicate remodeling-process links. Among the prioritized candidates, hsa-miR-708-5p was highlighted because it simultaneously targeted both LASSO-selected genes, *PABPC1* and *RPL4*. The integrated model suggests that dysregulated miRNA–hub gene interactions may contribute to LFH-associated fibrotic–immune remodeling. This proposed mechanism is hypothesis-generating and warrants further validation in future in vitro and in vivo studies.

Based on the integrated mRNA–miRNA network, hub gene analysis, LASSO-based candidate-feature prioritization, transcriptomic remodeling signatures, and candidate miRNA regulator prioritization, a proposed computational model was constructed (Figure 17).

The model suggests that dysregulated miRNAs may contribute to LFH-associated remodeling through coordinated suppression of key hub genes, particularly PABPC1 and RPL4, which were associated with ECM fibrosis, proteoglycan/GAG remodeling, and antigen-presentation pathways. Among the prioritized candidates, hsa-miR-708-5p emerged as the most prominent regulator because it simultaneously targeted both LASSO-selected genes, supporting its potential relevance within the proposed LFH-associated fibrotic–immune remodeling axis.

### 3.22. DGIdb-Based Drug–Gene Interaction Annotation of Hub Genes

To provide a pharmacologic annotation layer for the LFH-associated hub gene network, the CytoHubba-prioritized hub genes were queried in the Drug–Gene Interaction Database (DGIdb; available online: https://dgidb.org/; accessed on 24 June 2026). DGIdb integrates drug–gene interaction information from multiple curated sources and was used exclusively for annotation of database-curated pharmacologic associations. This analysis did not alter the DEG, inverse-regulated core-gene, STRING, CytoHubba, LASSO, or integrated miRNA–hub gene–signature results.

Across the queried hub gene set, DGIdb identified 183 drug–gene interaction entries involving 15 hub genes. Among these entries, 44 were annotated as approved and 139 as not approved in the DGIdb output. TOP2A showed the broadest pharmacologic annotation profile, with 117 total interactions, including 30 approved entries. The highest interaction score was observed for EEF1A1–sparsomycin (52.20), followed by CD74–milatuzumab (41.76). Among the annotated interactions, HMMR–hyaluronic acid was biologically consistent with the GAG and ECM remodeling axis identified in this study, whereas CD74-associated interactions were consistent with the antigen-presentation and immune-remodeling context of the LFH network. Selected DGIdb annotations are summarized in Table S36. These findings should be interpreted strictly as database-derived pharmacologic annotations of hub genes. They do not demonstrate therapeutic efficacy, direct target engagement, or clinical relevance in LFH. Any potential relevance of these drug–hub gene relationships to LFH-associated miRNA–hub gene axes will require direct in vitro validation in ligamentum flavum cells, followed by in vivo and clinical evaluation.

### 3.23. Database-Supported Annotation of Prioritized miRNA–Hub Gene Interactions

**Figure 18:**
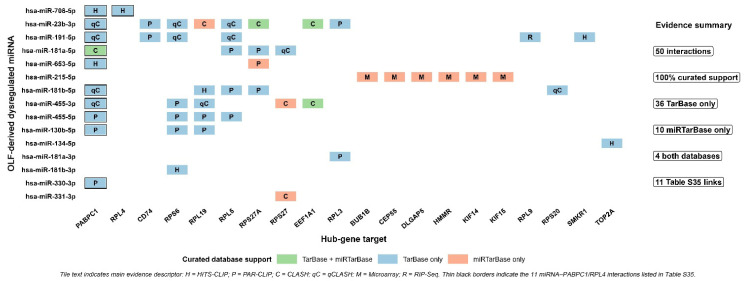
Database-supported evidence matrix of LFH-associated miRNA–hub gene interactions. Matrix summarizing database-supported evidence for the 50 candidate miRNA–hub gene interactions identified in the integrated LFH regulatory framework. Rows represent OLF-derived dysregulated miRNAs integrated into the LFH regulatory framework, whereas columns represent LFH-associated hub gene targets. Colored tiles indicate the curated database supporting each interaction: TarBase only (blue), miRTarBase only (orange), or support from both TarBase and miRTarBase (green). Tile labels denote the predominant experimental evidence descriptor associated with each interaction (H, HITS-CLIP; P, PAR-CLIP; C, CLASH; qC, qCLASH; M, Microarray; R, RIP-Seq). Thin black borders identify the 11 miRNA–PABPC1/RPL4 interactions listed in Table S35 and used to support the proposed computational model shown in Figure 17. The evidence summary panel indicates the total number of database-supported interactions and the distribution of supporting curated resources, including 36 interactions supported exclusively by TarBase, 10 supported exclusively by miRTarBase, and four supported by both databases. Database-supported validated interaction refers to records present in external curated miRNA–target interaction databases and does not indicate experimental validation in LFH tissue.

To further characterize the regulatory evidence underlying the LFH-associated miRNA–hub gene network, the 50 candidate miRNA–hub gene interactions listed in Tables S23 and S24 were systematically interrogated using the multiMiR resource. The corresponding candidate-miRNA prioritization and Figure 16-linked interaction summaries are presented in Tables S34 and S35, respectively, whereas evidence from experimentally curated miRNA–target interaction databases and computational prediction resources is provided in Table S37. The resulting database-supported evidence landscape is summarized in Figure 18. All 50 candidate interactions were represented in at least one external curated miRNA–target interaction database, indicating complete database-supported coverage of the integrated LFH regulatory network.

TarBase constituted the predominant source of curated interaction evidence, supporting 36 interactions, whereas miRTarBase uniquely supported 10 interactions. Four additional interactions were documented in both TarBase and miRTarBase, providing convergent support across curated repositories. By contrast, only two interactions were additionally represented in computational prediction resources, indicating that the overall evidence profile was dominated by experimentally curated database records rather than prediction-based annotations.

Within the integrative framework, hsa-miR-708-5p showed curated database support for interactions with both *PABPC1* and *RPL4*, the two genes selected by LASSO analysis. Curated interaction records were likewise identified for other prioritized regulatory miRNAs, including hsa-miR-23b-3p, hsa-miR-191-5p, hsa-miR-181a-5p, and hsa-miR-653-5p, across their corresponding hub gene targets, further supporting the biological plausibility of the proposed LFH regulatory network.

Overall, these findings provide an independent database-supported annotation layer for the integrated LFH miRNA–hub gene network (Figure 18; Table S37), showing that the proposed regulatory interactions are represented in established external miRNA–target interaction resources. Importantly, database-supported validated interactions refer exclusively to records present in external curated miRNA–target interaction databases and should not be interpreted as experimental validation of miRNA–target regulation in LFH tissue.

### 3.24. Transcription-Factor Enrichment of MCC-Prioritized Hub Genes

**Figure 19:**
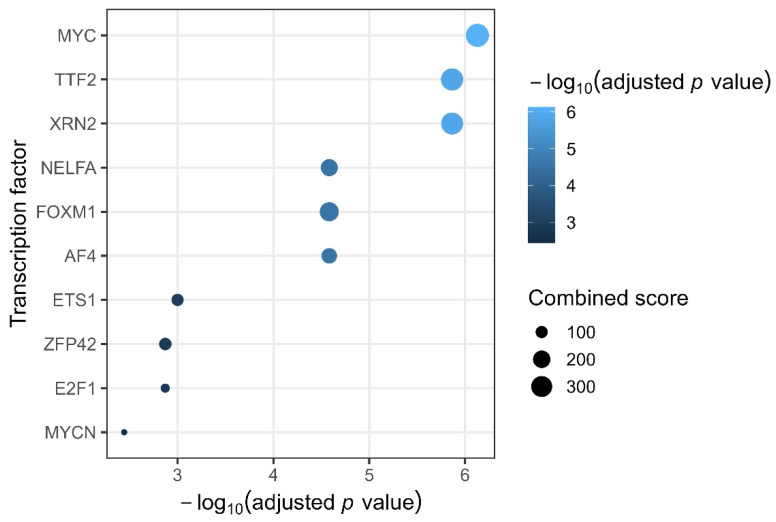
ChEA 2022 transcription-factor enrichment of MCC-prioritized hub genes in LFH. Bubble plot summarizing ChEA 2022 enrichment analysis of CytoHubba maximal clique centrality (MCC)-prioritized hub genes. The x-axis and color scale represent −log10 Benjamini–Hochberg-adjusted *p* value, and point size represents the Enrichr combined score. The plotted transcription factors represent the top non-duplicated ChEA 2022 terms after retaining the most significant record for each transcription factor. Full enrichment statistics and overlapping genes are provided in Table S38.

To annotate potential upstream regulatory context for the LFH-associated hub gene network, the CytoHubba maximal clique centrality (MCC)-prioritized hub genes were subjected to ChIP-based transcription-factor enrichment analysis using the ChEA 2022 library (Figure 19; Table S38). This analysis identified several significantly enriched transcription-factor target sets. *MYC* represented the top-ranked ChEA 2022 term, with the strongest statistical support (adjusted *p* = 7.43 × 10^−7^; combined score = 370.64; overlap = 15/2842), and was represented across multiple ChIP-derived datasets. *TTF2* and *XRN2* followed with comparable adjusted *p* values (1.37 × 10^−6^), whereas *NELFA, FOXM1*, and *AF4* also showed significant enrichment among the MCC-prioritized hub genes (Figure 19; Table S38).

The overlapping genes contributing to the top-ranked terms were dominated by ribosomal protein and translation-associated hub genes, including *RPL3*, *RPL4*, *RPL5*, *RPL9*, *RPL19*, *RPS6*, *RPS20*, *RPS27*, *RPS27A*, *EEF1A1*, and PABPC1. These findings suggest that the MCC hub gene layer is associated with database-derived transcription-factor target sets involving protein-synthesis and proliferative regulatory contexts. A supportive ENCODE-ChEA Consensus analysis showed a similar enrichment pattern, with *MYC* ranked among the leading terms together with *NELFE*, *TAF7*, *KAT2A*, *ATF2*, *E2F4*, *SIN3A*, *MAX*, and *RELA* (Table S39). Because ChEA and ENCODE-ChEA libraries integrate ChIP-derived datasets from diverse cell types and organisms, these results should be interpreted as database-derived upstream regulatory annotations rather than direct evidence of transcription-factor activity in LFH tissue.

## 4. Discussion

In the present study, we constructed an integrated mRNA–miRNA transcriptomic framework to characterize the molecular architecture of LFH and to prioritize candidate regulatory miRNAs associated with fibrotic–immune remodeling. By combining single-cell and bulk transcriptomic analyses with inverse miRNA–mRNA integration, PPI–based hub gene prioritization, machine-learning feature selection, and transcriptomic signature scoring, we identified a coordinated remodeling program dominated by ECM fibrosis, proteoglycan/GAG turnover, and antigen-presentation activity. Within this landscape, PABPC1 and RPL4 emerged as exploratory candidate hub features, and hsa-miR-708-5p was prioritized as a particularly relevant candidate regulator because it was linked to both genes in the integrated miRNA–hub gene network.

A central observation of this study was the predominance of fibroblast- and myofibroblast-associated transcriptional programs within hypertrophic ligamentum flavum tissue. Single-cell module-score analysis showed higher Fibroblast and Fibrosis_ECM signature scores in LSS_HLF cells relative to non-hypertrophic controls, consistent with enhanced fibrocellular activation. This pattern aligns with recent single-cell evidence demonstrating substantial fibroblast heterogeneity and active myofibroblast conversion as core cellular features of LFH [32]. Mechanistically, fibroblast-to-myofibroblast transition in the ligamentum flavum has been shown to proceed through the canonical TGF-β/Smad2/3 axis, with regulatory checkpoints such as Sirt1 modulating the dynamic progression of this transition [33]. These findings provide biological support for the coordinated activation of ECM–fibrosis and Fibroblast signatures observed in hypertrophic tissue and reinforce the interpretation that stromal activation is a dominant driver of LFH-associated remodeling. However, our integrative framework was designed to characterize the downstream transcriptomic architecture of established LFH rather than to directly identify the initiating cause of the remodeling process. Based on prior experimental and mechanistic studies, chronic mechanical overload and repetitive micro-injury likely act as upstream triggers by inducing local inflammation, macrophage recruitment, and TGF-β1-centered fibroblast activation, thereby establishing a self-reinforcing fibrotic loop [4,6]. Additional modulation through GSK-3β/β-catenin and SERPINE2/β-catenin signaling further supports the view that the fibrotic–immune programs captured here most likely represent downstream molecular correlates of a mechanically driven and age-associated remodeling cascade rather than the primary initiating event itself [5,7,8,34].

The integrated analysis revealed coexisting fibrotic and immune-associated transcriptional programs that were not uniformly represented across analytical layers. While bulk-level GSEA highlighted antigen presentation, MHC class I/II programs, and leukocyte/macrophage migration, the single-cell dataset showed comparatively lower immune-signature scores alongside strong fibroblast enrichment. Rather than representing a contradiction, this pattern is consistent with the current understanding of fibrosis as a spatially and temporally organized process in which macrophage–fibroblast crosstalk is activated in a context-dependent manner rather than uniformly across all cellular compartments and disease stages [35,36]. The dynamic and stage-dependent activation of macrophage populations during fibrotic progression [35], together with the bidirectional signaling that couples innate immune cells to stromal effector cells [36], offers a coherent explanation for why immune-remodeling signals may be more readily captured at the bulk transcriptomic level while a single-cell snapshot is dominated by stromal expansion.

Beyond classical collagen-centered fibrosis, our enrichment analyses consistently highlighted proteoglycan and GAG remodeling programs, including proteoglycan metabolism and biosynthesis, chondroitin sulfate and dermatan sulfate proteoglycan metabolism, and GAG biosynthetic and catabolic processes. This broadened ECM signature, together with the upregulation of CHST10 in the bulk dataset, supports a contemporary view of organ fibrosis in which non-collagenous matrix constituents, basement-membrane proteins, and proteoglycan/GAG turnover are integral to fibroblast phenotype and tissue stiffening rather than secondary phenomena [37]. Framing LFH within this expanded ECM-remodeling paradigm strengthens the biological plausibility of the proteoglycan/GAG signatures identified here and indicates that ligamentum flavum fibrosis cannot be fully explained by collagen accumulation alone.

The prioritization of CD74 as an antigen-presentation-associated hub gene further connects the fibrotic and immune dimensions of LFH. CD74 has been mechanistically implicated as an active signaling node in fibrosis, where the MIF/CD74 axis in stromal effector cells promotes fibrogenic activation rather than serving merely as an immunological marker [38]. Within the integrated network, this finding supports the interpretation that CD74 may represent a fibro-inflammatory effector node linking antigen-presentation activity to ECM remodeling, consistent with the convergent fibrotic–immune architecture identified by the integrated analyses.

The MCC-prioritized hub gene module was strongly enriched for ribosomal and translation-associated genes, including *PABPC1* and multiple RPL/RPS family members. The transcription-factor enrichment analysis added an upstream regulatory annotation layer to the MCC-prioritized LFH hub gene network. In the ChEA 2022 analysis, MYC was the most significantly enriched transcription-factor term (adjusted *p* = 7.43 × 10^−7^; combined score = 370.6) and appeared across multiple ChIP-derived datasets, suggesting that the MYC target-gene repertoire overlaps substantially with the LFH hub gene set (Figure 19; Table S38). A supportive ENCODE-ChEA Consensus analysis also ranked *MYC* among the leading terms from both CHEA and ENCODE evidence sources, together with additional factors including *NELFE*, *TAF7*, *KAT2A*, *ATF2*, *E2F4*, *SIN3A*, *MAX*, and *RELA* (Table S39). The genes contributing to the top-ranked ChEA 2022 terms were dominated by ribosomal protein and translation-associated hub genes, including *RPL3*, *RPL4*, *RPL5*, *RPL9*, *RPL19*, *RPS6*, *RPS20*, *RPS27*, *RPS27A*, *EEF1A1*, and *PABPC1*. This pattern is consistent with enrichment of transcription-factor target sets related to translational capacity, ribosome-associated biology, and proliferative regulatory programs. In the context of the present study, this finding complements the hub gene and miRNA-prioritization results by suggesting that the LFH regulatory network contains an upstream annotation layer linked to transcriptional control of translation-associated hub genes.

An important interpretive caveat concerns the discordant direction of *PABPC1* and *RPL4* between the bulk and single-cell datasets. In GSE113212, both genes were reduced in the Old/LFH group, whereas in GSE294458 both showed higher mean expression in LSS_HLF cells than in LDH_NLF cells. This discordance is biologically and methodologically important because *PABPC1* and *RPL4* are central exploratory candidate hub features in the integrative model. The discrepancy may reflect differences in platform, sample structure, analytical resolution, age–disease composition, and cell-state or cell-type composition between bulk tissue and single-cell profiles. Accordingly, *PABPC1* and *RPL4* should not be interpreted as directionally validated biomarkers across datasets. Instead, their role in the present study is limited to exploratory candidate-feature prioritization within the GSE113212 discovery cohort and network-level regulatory hypothesis generation. Future LFH-specific studies using donor-level, age-aware, and preferably paired bulk/single-cell designs will be required to determine whether these genes show consistent disease-associated regulation in defined ligamentum flavum cell populations.

The transcription-factor enrichment findings should be interpreted cautiously. ChEA and ENCODE-ChEA analyses identify transcription factors whose published ChIP-derived target-gene sets significantly overlap with the input hub genes; they do not demonstrate transcription-factor activation, direct binding in ligamentum flavum tissue, or causal regulation in LFH. In addition, the source ChIP datasets were generated across diverse cell types, organisms, and experimental contexts. Therefore, the *MYC*-centered enrichment pattern should be regarded as a database-derived, hypothesis-generating upstream regulatory annotation that warrants future validation in LFH tissue and experimental models.

At the regulatory layer, several prioritized miRNAs are biologically consistent with fibrotic and aging-associated remodeling. miR-181a-5p, which was linked to PABPC1 in the integrated miRNA–hub gene network, has been shown to attenuate fibrotic progression by directly targeting TGFBR1 and modulating epithelial–mesenchymal transition in experimental fibrosis, situating it within the same TGF-β-centered axis that dominates LFH pathology [39]. miR-23b-3p, the broadest hub gene-targeting candidate in our analysis, has been mechanistically linked to cellular senescence and altered metabolic remodeling [40], which is particularly relevant given that our bulk discovery cohort compared older hypertrophic ligamentum flavum samples with younger non-hypertrophic control samples. This aging dimension is further contextualized by evidence that increasing matrix stiffness can drive myofibroblast senescence and sustain fibrotic progression [34], suggesting that age-associated changes in the mechanical and matrix microenvironment may reinforce the remodeling programs captured in our datasets. Within this framework, hsa-miR-708-5p was prioritized as the highest-priority candidate because it was linked to both LASSO-selected genes, *PABPC1* and *RPL4*, in the integrated miRNA–hub gene network; however, given that the miRNA layer was derived from an ossification-associated dataset and integrated as a complementary regulatory resource, this candidate should be regarded as a hypothesis-generating regulator rather than an established LFH-specific effector.

From a biological and translational perspective, our drug–gene annotation analysis identified pharmacologically annotated targets among the hub genes. Although these DGIdb-derived annotations suggest that selected miRNA–hub gene axes may intersect with pharmacologically annotated targets, they remain hypothesis-generating and require direct experimental validation in ligamentum flavum cells, followed by in vivo and clinical evaluation before any therapeutic relevance can be inferred. Encouragingly, recent experimental work has demonstrated that targeted suppression of fibroblast activation and ECM remodeling via the TGF-β/SMAD pathway can attenuate LFH in preclinical models [41], providing proof-of-concept that the fibrotic axis emphasized in our analyses is pharmacologically tractable and supporting the rationale for future miRNA- and pathway-directed intervention studies.

An additional strength of the present study is the incorporation of an external database-supported annotation layer for the prioritized miRNA–hub gene network. All 50-candidate miRNA–hub gene interactions identified through the integrative analytical framework had database-supported annotations in at least one experimentally curated miRNA–target interaction resource, with the majority supported by TarBase and a smaller subset by miRTarBase or both repositories. This observation increases confidence that the proposed regulatory relationships are not isolated computational predictions but have been independently documented in established miRNA–target interaction resources. Importantly, this database-supported evidence should be interpreted as external regulatory annotation rather than experimental validation in LFH tissue. Nevertheless, the concordance between the integrative transcriptomic framework and curated interaction databases provides additional biological credibility for the proposed regulatory network, particularly for the prioritized hsa-miR-708-5p–PABPC1 and hsa-miR-708-5p–RPL4 interactions highlighted in the proposed computational model.

Several limitations should be considered when interpreting these findings. First, all analyses relied on the secondary reuse of publicly available transcriptomic datasets with relatively small sample sizes, which limits statistical power and generalizability. Second, the single-cell dataset (GSE294458) contained only one specimen per group, so all single-cell comparisons should be regarded as exploratory and sample-associated rather than population-level. Third, the dysregulated miRNAs were derived from ossified ligamentum flavum tissue (GSE106256) rather than from hypertrophic ligamentum flavum, and were therefore used only as a complementary regulatory resource rather than as LFH-specific events. Fourth, all regulatory relationships were inferred computationally and were not experimentally validated. Accordingly, the prioritized miRNAs and hub genes are presented as candidates for future experimental testing rather than as established diagnostic or therapeutic targets.

Despite these limitations, the integration of differential-expression analysis, miRNA–mRNA regulatory network construction, PPI analysis, LASSO-based candidate-feature prioritization, transcriptomic remodeling signatures, database-supported regulatory annotation, and single-cell contextualization provides a systems-level framework for understanding LFH-associated fibrotic–immune remodeling.

## 5. Conclusions

This study provides an integrated systems-level reconstruction of the transcriptomic architecture underlying LFH combining single-cell and bulk mRNA profiling, inverse miRNA–mRNA integration, hub gene prioritization, LASSO-based candidate-feature selection, remodeling-signature scoring, and external database-supported annotation within a unified analytical framework. The principal conceptual contribution is the characterization of LFH as a coordinated fibrotic–immune remodeling process rather than an isolated fibrotic phenotype, with ECM fibrosis, proteoglycan/GAG turnover, and antigen-presentation activity converging on a compact hub gene network enriched for translation-associated regulators. Within this framework, *PABPC1* and *RPL4* emerged as exploratory candidate hub features associated with fibrotic and proteoglycan-related remodeling signatures, rather than as independently validated diagnostic biomarkers. Integration across the regulatory layers placed hsa-miR-708-5p at the forefront because it was linked to both *PABPC1* and *RPL4* and received concordant annotation from external miRNA–target databases.

Additional candidates, including hsa-miR-23b-3p, hsa-miR-191-5p, hsa-miR-181a-5p, and hsa-miR-653-5p, further support a convergent regulatory topology centered on *PABPC1*, *CD74*, and the broader ribosomal hub gene module. From a translational pharmacology perspective, these findings do not establish therapeutic efficacy or direct clinical actionability. Rather, they provide a prioritized regulatory map that may guide future mechanistic studies. The most immediate next step is experimental validation of the prioritized miRNA–hub gene axes, particularly the hsa-miR-708-5p–PABPC1/RPL4 relationships, using gain- and loss-of-function approaches in ligamentum flavum fibroblasts, followed by assessment of fibrotic, proteoglycan/GAG, and antigen-presentation readouts. In this context, the framework defined here offers a coherent hypothesis-generating foundation for future mechanistic and regulatory investigations in LFH.

## Figures and Tables

**Table 1:** Significant differentially expressed genes identified in GSE113212 (Old vs. Young ligamentum flavum samples).

Gene	Probe_ID	Regulation	logFC	AveExpr	T	*p* Value	Adjusted *p* Value	B
*ULBP1*	A_33_P3422802	Upregulated	3.0750	1.388 × 10^−17^	9.571	1.251 × 10^−5^	0.0498	3.553
*CHRM3*	A_23_P401472	Upregulated	2.2500	−2.776 × 10^−17^	11.503	3.183 × 10^−6^	0.0498	4.568
*BMPR1B*	A_24_P63380	Upregulated	2.1200	−2.776 × 10^−17^	12.963	1.288 × 10^−6^	0.0461	5.173
*CHST10*	A_33_P3277198	Upregulated	1.7250	4.163 × 10^−17^	9.721	1.115 × 10^−5^	0.0498	3.643
*TGFB1*	A_24_P79054	Upregulated	1.6200	−1.388 × 10^−17^	11.091	4.186 × 10^−6^	0.0498	4.374
*COL13A1*	A_23_P1331	Upregulated	1.5925	1.250 × 10^−3^	10.096	8.431 × 10^−6^	0.0498	3.858
*MDM2*	A_23_P502750	Upregulated	1.2650	1.388 × 10^−17^	9.740	1.100 × 10^−5^	0.0498	3.654
*MYADML*	A_33_P3422923	Downregulated	−4.0750	2.776 × 10^−17^	−10.110	8.348 × 10^−6^	0.0498	3.865
*LOC283404*	A_33_P3606692	Downregulated	−2.2175	1.250 × 10^−3^	−10.580	5.955 × 10^−6^	0.0498	4.118

Differential expression analysis of GSE113212 was performed using the limma framework. Significant DEGs were defined as genes meeting Benjamini–Hochberg-adjusted *p* value < 0.05 and absolute log2 fold change > 1. Among 35,789 analyzed probes/transcripts, nine significant DEGs were identified, including seven upregulated and two downregulated genes in Old ligamentum flavum samples compared with Young controls.

**Table 2:** Supportive topological centrality metrics for the MCC-prioritized hub genes. Degree, closeness, and betweenness centrality values were calculated using cytoHubba for the STRING PPI network of the inverse-regulated core genes. The table reports the 20 MCC-prioritized hub genes visualized in Figure 8 and summarizes their supportive centrality profiles. Genes are ordered by descending degree score.

Rank	Gene	STRING Protein ID	Degree	Closeness	Betweenness
1	*RPS27A*	9606.ENSP00000272317	17	78.16429	9662.51563
2	*RPS6*	9606.ENSP00000369757	15	65.77540	1036.66985
3	*RPL5*	9606.ENSP00000359345	14	65.27540	809.33175
4	*RPL4*	9606.ENSP00000311430	13	64.77540	333.33175
5	*BUB1B*	9606.ENSP00000287598	13	58.98456	2123.93800
6	*RPL3*	9606.ENSP00000346001	12	63.00437	102.10441
7	*RPS20*	9606.ENSP00000429374	12	63.00437	102.10441
8	*EEF1A1*	9606.ENSP00000339063	11	62.50437	529.50957
9	*RPS27*	9606.ENSP00000499044	11	62.50437	54.28735
10	*RPL19*	9606.ENSP00000225430	11	62.50437	47.86151
11	*RPL9*	9606.ENSP00000494697	11	62.50437	47.86151
12	*CD74*	9606.ENSP00000009530	10	61.83770	46.84564
13	*TOP2A*	9606.ENSP00000411532	10	54.70004	670.96350
14	*CEP55*	9606.ENSP00000360540	9	54.20004	194.96350
15	*DLGAP5*	9606.ENSP00000247191	9	54.20004	194.96350
16	*KIF15*	9606.ENSP00000324020	8	53.70004	143.99967
17	*HMMR*	9606.ENSP00000377492	8	53.53337	93.10250
18	*PABPC1*	9606.ENSP00000313007	8	52.54921	8.00000
19	*SMKR1*	9606.ENSP00000454370	8	52.38254	0.00000
20	*KIF14*	9606.ENSP00000356319	7	47.89134	0.33333

**Table 3:** MCC and EPC scores of the MCC-prioritized hub genes. MCC was used as the principal cytoHubba ranking method for downstream hub gene prioritization. EPC scores are shown as a complementary topology metric for the same 20 hub genes. Genes are ordered by descending MCC score and correspond to the hub gene network shown in Figure 8.

Rank	Gene	STRING Protein ID	MCC	EPC
1	*RPS6*	9606.ENSP00000369757	771,130	51.812
2	*RPL5*	9606.ENSP00000359345	771,127	51.741
3	*RPL4*	9606.ENSP00000311430	771,126	51.523
4	*RPS20*	9606.ENSP00000429374	771,120	51.667
5	*RPL3*	9606.ENSP00000346001	771,120	51.647
6	*RPL9*	9606.ENSP00000494697	766,080	51.305
7	*RPL19*	9606.ENSP00000225430	766,080	51.204
8	*RPS27*	9606.ENSP00000499044	730,800	50.743
9	*RPS27A*	9606.ENSP00000272317	725,768	52.524
10	*CD74*	9606.ENSP00000009530	403,200	51.303
11	*EEF1A1*	9606.ENSP00000339063	367,921	50.791
12	*SMKR1*	9606.ENSP00000454370	40,320	49.711
13	*PABPC1*	9606.ENSP00000313007	5042	47.943
14	*BUB1B*	9606.ENSP00000287598	2288	37.693
15	*TOP2A*	9606.ENSP00000411532	2281	36.343
16	*CEP55*	9606.ENSP00000360540	2280	36.727
17	*DLGAP5*	9606.ENSP00000247191	2280	35.548
18	*HMMR*	9606.ENSP00000377492	2160	35.947
19	*KIF15*	9606.ENSP00000324020	1560	35.501
20	*KIF14*	9606.ENSP00000356319	1440	34.796

## Data Availability

The datasets analyzed in this study are publicly available from the NCBI Gene Expression Omnibus (GEO) database under accession numbers GSE294458, GSE113212, GSE106256, GSE106253, and GSE267819. No new sequencing or transcriptomic datasets were generated in the present study. Additional supporting results generated during the analyses are provided in the Supplementary Materials and the accompanying Supplementary_Tables.xlsx workbook. Further information regarding the analyses performed in this study is available from the corresponding author upon reasonable request.

## Supplementary Materials

The following supporting information can be downloaded at: https://www.mdpi.com/article/10.3390/biomedicines14071614/s1, Figure S1: Additional quality control and dimensionality-reduction diagnostics for GSE294458; Figure S2: Sample-level distribution of cells in the GSE294458 UMAP space; Figure S3: Principal component analysis of GSE113212 samples; Figure S4: Differential gene-expression analysis of GSE113212 Old versus Young ligamentum flavum samples; Figure S5: Exploratory biologically selected gene-expression signature between LSS_HLF and LDH_NLF cells in GSE294458; Figure S6: Quality control and sample-balanced visualization of GSE267819 ligamentum flavum single-cell RNA-seq data; Figure S7: Marker-level evaluation of the balanced GSE267819 single-cell dataset; Figure S8: Supportive transcript-level analysis of GSE106253; Figure S9: Volcano plot of differentially expressed microRNAs in the GSE106256 OLF miRNA-sequencing dataset; Figure S10: Reactome pathway enrichment analysis of the 651 inverse-regulated core genes from integrated mRNA–miRNA analysis; Figure S11. Separation-aware sensitivity analyses of the exploratory PABPC1–RPL4 candidate-feature score in GSE113212; Table S1: Cell counts and quality control metrics before and after filtering; Table S2: Representative top marker genes for each Seurat cluster; Table S3: Selected biologically relevant marker genes supporting cluster interpretation; Table S4: Cluster-wise cell counts and proportions by specimen; Table S5: Significant differentially expressed genes in GSE113212; Table S6: Upregulated DEGs in Old ligamentum flavum samples; Table S7: Downregulated DEGs in Old ligamentum flavum samples; Table S8: Exploratory cell-level differential-expression summary for the LSS_HLF and LDH_NLF specimens in GSE294458; Table S9: Selected biologically relevant genes upregulated in LSS_HLF cells; Table S10: Selected biologically relevant genes downregulated in LSS_HLF cells; Table S11: Quality control summary of GSE267819 after filtering; Table S12: Sample counts before balancing in GSE267819; Table S13: Balanced sample counts in GSE267819; Table S14: Balanced Seurat cluster counts in GSE267819; Table S15: Sample fractions within balanced Seurat clusters in GSE267819; Table S16: Canonical marker gene presence in GSE267819; Table S17: Average canonical marker expression by Seurat cluster in GSE267819; Table S18: Significant differentially expressed microRNAs in GSE106256; Table S19: Complete list of the 651 inverse-regulated core genes identified through integrated mRNA–miRNA analysis; Table S20: Gene Ontology Biological Process (GO-BP) enrichment analysis of the 651 inverse-regulated core genes; Table S21: KEGG pathway enrichment analysis of the 651 inverse-regulated core genes; Table S22: Reactome pathway enrichment analysis of the 651 inverse-regulated core genes; Table S23: Core miRNA–hub gene regulatory network identified in LFH; Table S24: Complete miRNA–hub gene regulatory-network edge list; Table S25: Discovery-cohort discrimination, internal stability, and separation-aware sensitivity analyses of the exploratory PABPC1–RPL4 candidate-feature score in GSE113212; Table S26: Cross-cohort comparison of LASSO-selected genes identified in GSE113212 and evaluated in GSE294458; Table S27: Immune and fibrosis signature-score column mapping for GSE294458 single-cell signature analysis; Table S28: Descriptive summary of immune and fibrosis signature scores in the GSE294458 single-cell dataset.; Table S29: Exploratory cell-level comparison of immune and fibrosis signature scores between the LDH_NLF and LSS_HLF specimens in GSE294458; Table S30: Prioritization of candidate miRNA regulators based on hub gene targeting capacity; Table S31: KEGG gene set enrichment analysis (GSEA) results from the ranked GSE113212 bulk transcriptomic dataset; Table S32: Gene Ontology Biological Process (GO-BP) gene set enrichment analysis (GSEA) results from the ranked GSE113212 bulk transcriptomic dataset; Table S33: Sample-level fibrosis–immune signature scores (S33A), Spearman correlation with LASSO-selected genes (S33B), and complete signature gene lists with leave-one-gene-out sensitivity analysis (S33C) in the GSE113212 bulk transcriptomic dataset; Table S34: Prioritization of candidate miRNA regulators linked to fibrotic–immune remodeling; Table S35: miRNA–Figure 16 gene–signature links; Table S36: Drug–gene interactions of LFH-associated hub genes retrieved from DGIdb; Table S37: Database-supported annotation of LFH-associated miRNA–hub gene interactions; Table S38: ChEA 2022 transcription-factor enrichment analysis of CytoHubba MCC-prioritized hub genes; Table S39: ENCODE-ChEA Consensus transcription-factor enrichment analysis of CytoHubba MCC-prioritized hub genes.

## Abbreviations

The following abbreviations are used in this manuscript:

AUC	Area under the curve
ChEA	ChIP Enrichment Analysis
ChIP	Chromatin immunoprecipitation
CILP	Cartilage intermediate layer protein
DEG	Differentially expressed gene
DEM	Differentially expressed microRNA
DGIdb	Drug–Gene Interaction Database
ECM	Extracellular matrix
ENCODE	Encyclopedia of DNA Elements
EPC	Edge-Percolated Component
FDR	False discovery rate
GAG	Glycosaminoglycan
GEO	Gene Expression Omnibus
GO	Gene Ontology
GO-BP	Gene Ontology Biological Process
GSEA	Gene set enrichment analysis
KEGG	Kyoto Encyclopedia of Genes and Genomes
LASSO	Least absolute shrinkage and selection operator
LDH	Lumbar disk herniation
LDH_NLF	Lumbar disk herniation–associated non-hypertrophic ligamentum flavum
LFH	Ligamentum flavum hypertrophy
logFC	Log2 fold change
LSS	Lumbar spinal stenosis
LSS_HLF	Lumbar spinal stenosis–associated hypertrophic ligamentum flavum
MCC	Maximal clique centrality
MHC	Major histocompatibility complex
MIF	Macrophage migration inhibitory factor
miRNA	MicroRNA
mRNA	Messenger RNA
NCBI	National Center for Biotechnology Information
NES	Normalized enrichment score
OLF	Ossified ligamentum flavum
PCA	Principal component analysis
PPI	Protein–protein interaction
ROC	Receiver operating characteristic
scRNA-seq	Single-cell RNA sequencing
STRING	Search Tool for the Retrieval of Interacting Genes/Proteins
TCA	Tricarboxylic acid
TGF-β1	Transforming growth factor beta-1
UMAP	Uniform manifold approximation and projection
UMI	Unique molecular identifier
