# Supplementary material for: Prioritization of Candidate miRNA Regulators Targeting Fibrotic–Immune Remodeling in Ligamentum Flavum Hypertrophy: An Integrated mRNA–miRNA Transcriptomic Study
Source: Biomedicines. 2026 Jul 17;14(7):1614. doi: 10.3390/biomedicines14071614 (PMC13406011; doi:10.3390/biomedicines14071614)
Supplement: Supplementary file 1 [file biomedicines-14-01614-s001.zip › Supplementary Materials.pdf]

Supplementary Materials

Prioritization of candidate miRNA regulators targeting fibrotic–immune remodeling in ligamentum flavum hypertrophy: an integrated mRNA–miRNA transcriptomic study

Overview

This Supplementary Materials file provides the supporting figures and concise summary tables accompanying the main manuscript. It includes additional QC diagnostics, sample-level UMAP visualizations, supplementary analyses of the independent LFH-only single-cell dataset (GSE267819), supportive transcript-level analyses, miRNA differential-expression visualization, Reactome pathway enrichment results, and compact summary tables supporting the miRNA–hub gene network, candidate miRNA regulator prioritization, GSEA, remodeling-signature, integrative regulatory-model, pharmacologic-annotation, database-supported miRNA–target annotation, and transcription-factor enrichment analyses presented in the main manuscript. Supplementary Tables S1–S18, S33A–S36, S38, and S39 are included in this document. Complete Supplementary Tables S19–S32 and S**37** are provided in the accompanying Excel workbook, Supplementary_Tables.xlsx. Descriptive entries for Tables S19–S**32** and S**37** are also included below to facilitate cross-referencing. Each worksheet in the Excel workbook is labeled according to the corresponding supplementary table number.

Supplementary Figures

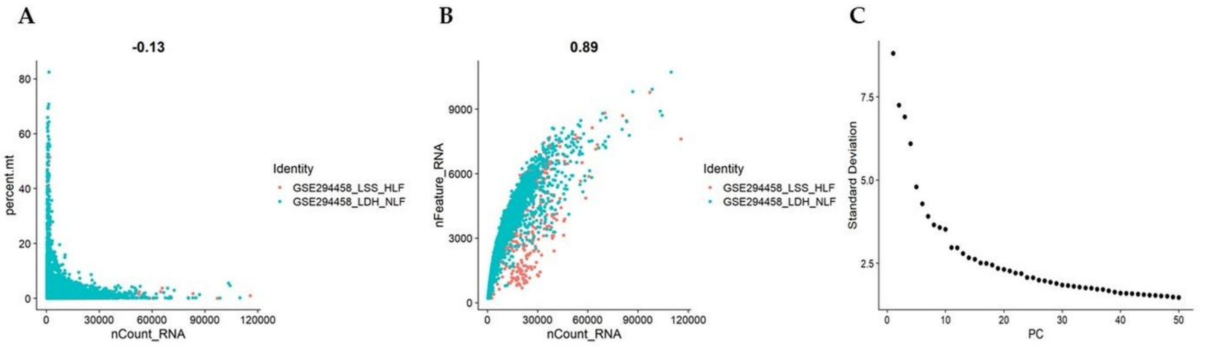

Figure S1. Additional quality-control and dimensionality-reduction diagnostics for GSE294458. (A) Scatter plot showing the relationship between total UMI counts per cell (nCount_RNA) and mitochondrial transcript percentage (percent.mt). (B) Scatter plot showing the relationship between total UMI counts per cell (nCount_RNA) and the number of detected genes per cell (nFeature_RNA). (C) Elbow plot showing the standard deviation explained by principal components used to guide dimensionality reduction and downstream clustering.

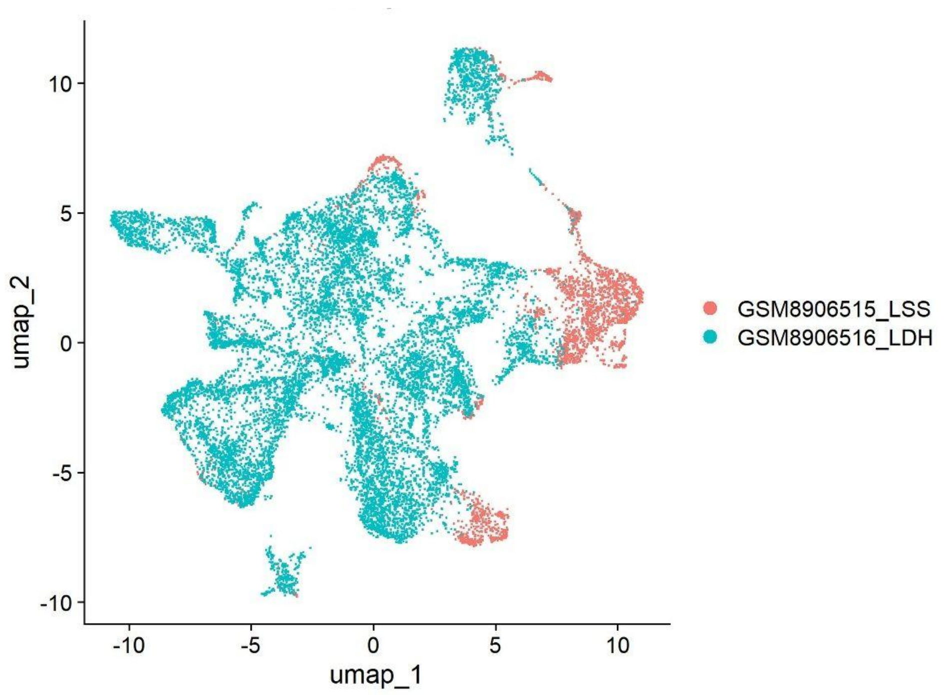

Figure S2. Sample-level distribution of cells in the GSE294458 UMAP space. UMAP visualization of quality-filtered cells colored by sample identity. The LSS sample corresponds to GSM8906515_LSS, and the LDH sample corresponds to GSM8906516_LDH. This plot is provided to document sample-level distribution across the integrated single-cell landscape.

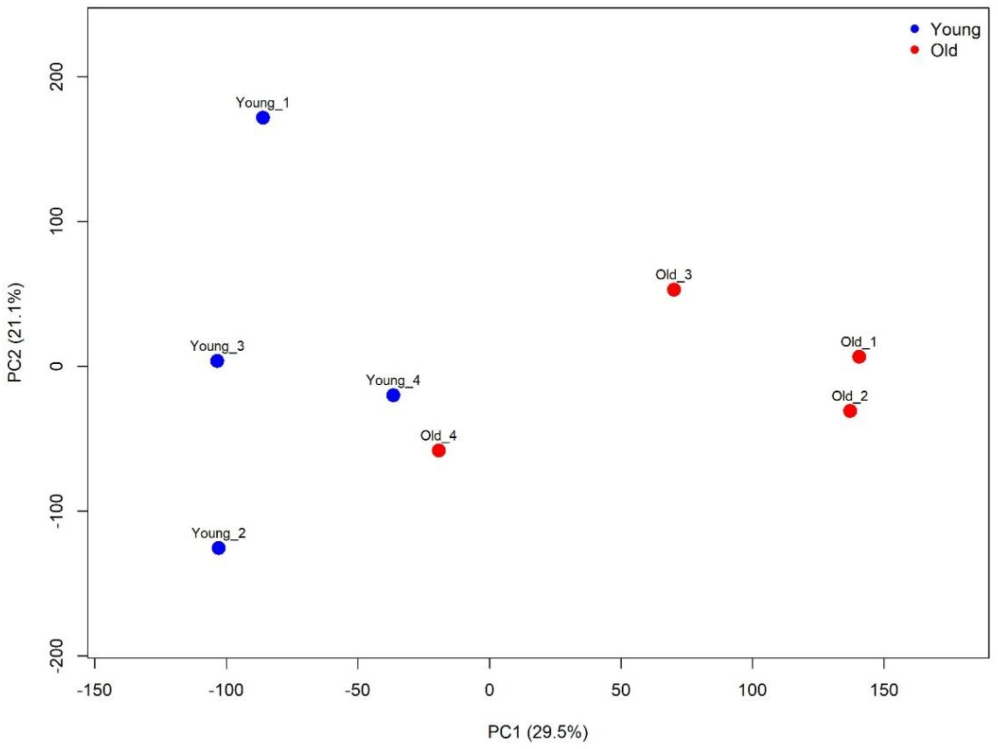

Figure S3. Principal component analysis of GSE113212 samples. Principal component analysis was performed using the normalized expression matrix of GSE113212 samples. Each dot represents one sample. Blue dots indicate Young ligamentum flavum samples, and red dots indicate Old ligamentum flavum samples. PC1 and PC2 explained 29.5% and 21.1% of the total variance, respectively.

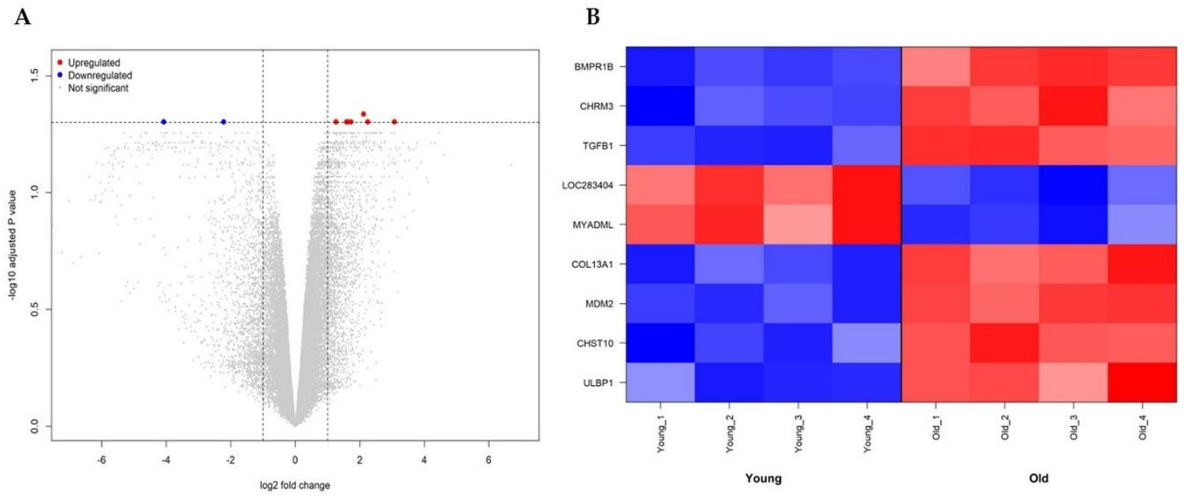

Figure S4. Differential gene-expression analysis of GSE113212 Old versus Young ligamentum flavum samples. (A) Volcano plot showing differentially expressed transcripts between Old and Young ligamentum flavum samples in GSE113212. Red dots indicate significantly upregulated genes in Old samples, blue dots indicate significantly downregulated genes in Old samples, and grey dots indicate non-significant transcripts. Differential expression was defined using Benjamini–Hochberg adjusted p value < 0.05 and absolute logFC > 1. A total of 9 significant differentially expressed genes were identified, including 7 upregulated and 2 downregulated genes in Old ligamentum flavum samples. (B) Heatmap showing the expression patterns of the 9 significant differentially expressed genes identified in the Old versus Young comparison. Rows represent genes and columns represent individual samples. Expression values were transformed into row-wise z-scores. Red indicates relatively higher expression, whereas blue indicates relatively lower expression. Young samples are shown on the left and Old samples on the right.

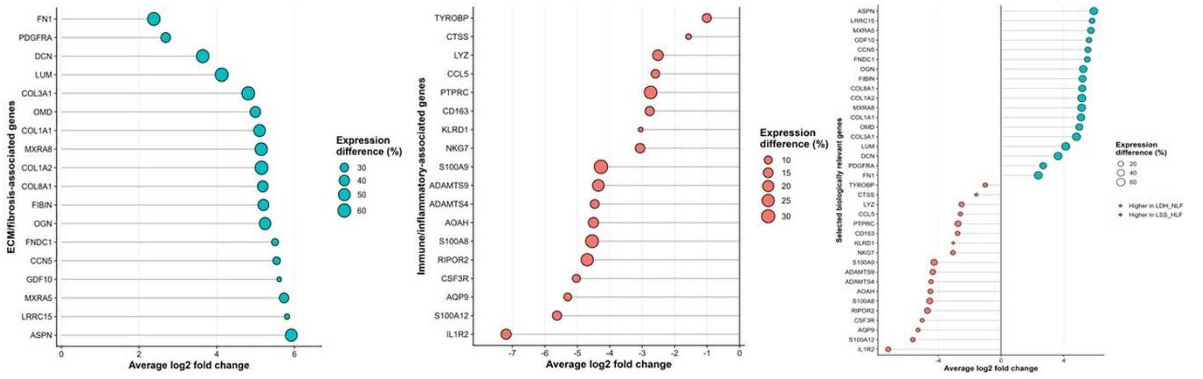

Figure S5. Exploratory biologically selected gene-expression signature between LSS_HLF and LDH_NLF cells in GSE294458. (A) Lollipop plot showing selected extracellular matrix, stromal, and fibrosis-associated genes with higher average log2 fold change in LSS_HLF cells. Dot size represents the absolute difference in the percentage of expressing cells between groups. (B) Lollipop plot showing selected immune, inflammatory, and myeloid-associated genes with lower average log2 fold change in LSS_HLF cells, corresponding to relatively higher expression in LDH_NLF cells. Dot size represents the absolute difference in the percentage of expressing cells between groups. (C) Bidirectional dot-lollipop summary of selected biologically relevant genes from both signatures. Positive average log2 fold change indicates higher expression in LSS_HLF cells, whereas negative average log2 fold change indicates higher expression in LDH_NLF cells. Genes were selected from significant differentially expressed genes based on biological relevance to extracellular matrix

remodeling, fibroblast/stromal activation, immune signaling, and inflammatory cell-associated transcriptional features. Because GSE294458 contains one specimen per condition, these results should be interpreted as exploratory, sample-associated observations rather than donor-level evidence.

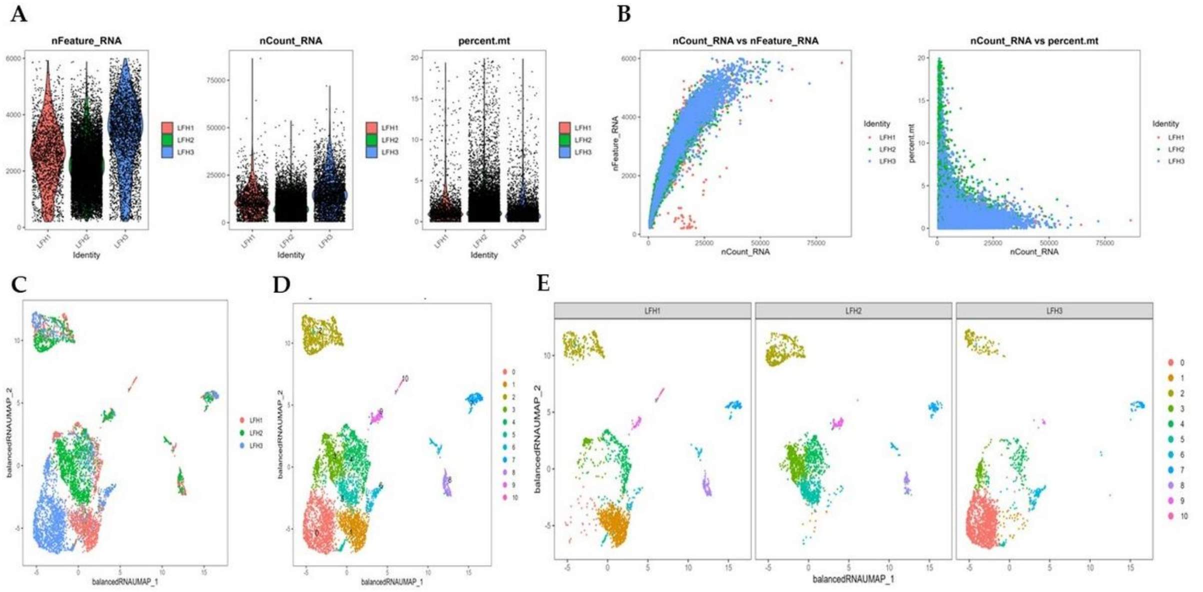

Figure S6. Quality control and sample-balanced visualization of GSE267819 ligamentum flavum single-cell RNA-seq data. (A) Violin plots showing nFeature_RNA, nCount_RNA, and percent.mt after quality filtering in LFH1, LFH2, and LFH3. (B) Scatter plots illustrating the relationships between nCount_RNA and nFeature_RNA and between nCount_RNA and percent.mt. (C–E) UMAP representation of the balanced object, shown by sample identity, Seurat cluster, and split sample view, respectively. These panels document the quality of the processed LFH-only dataset and the distribution of the three samples after balancing. As no non-LFH control sample is present in GSE267819, these data were used only for supplementary visualization and marker-level assessment, not for disease–control differential expression.

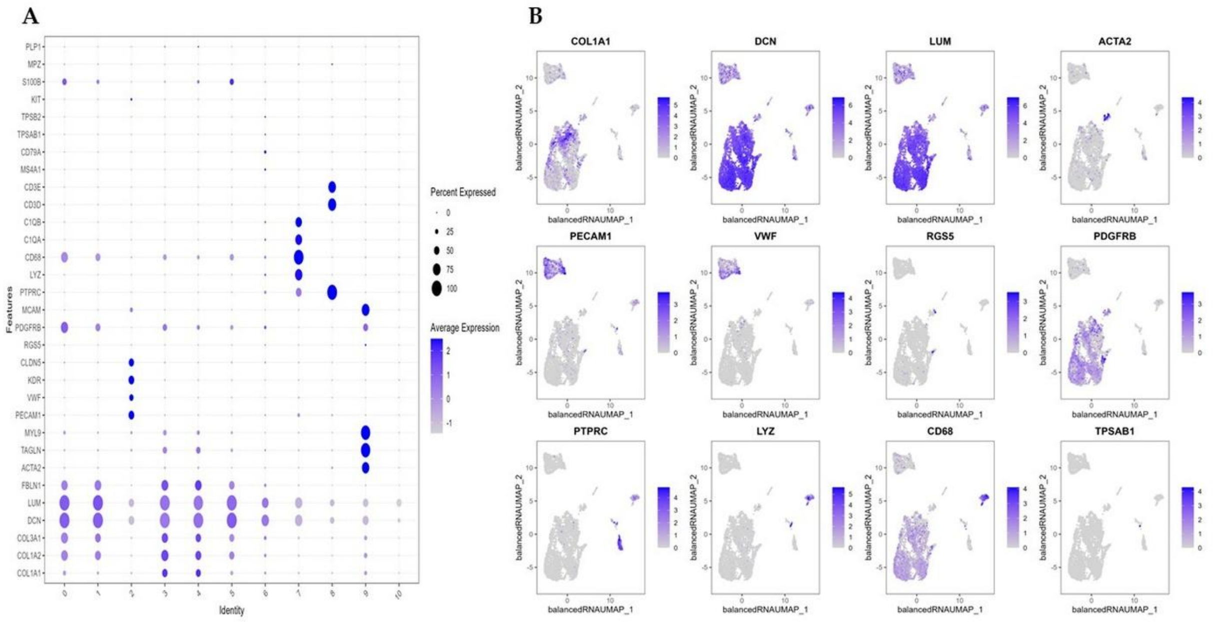

Figure S7. Marker-level evaluation of the balanced GSE267819 single-cell dataset. (A) Dot plot summarizing the expression of selected canonical markers across Seurat clusters. Dot size denotes the proportion of cells expressing each marker, and color intensity denotes average expression. (B) Feature plots showing the spatial distribution of representative canonical markers on the balanced UMAP. Together, these plots provide marker-level support for stromal, matrix-associated, contractile, endothelial, immune, and mast-cell-related transcriptional patterns within the LFH samples. Given the absence of a non-LFH control group, these findings were interpreted as supportive marker evidence rather than definitive cell-type annotation or disease–control differential expression.

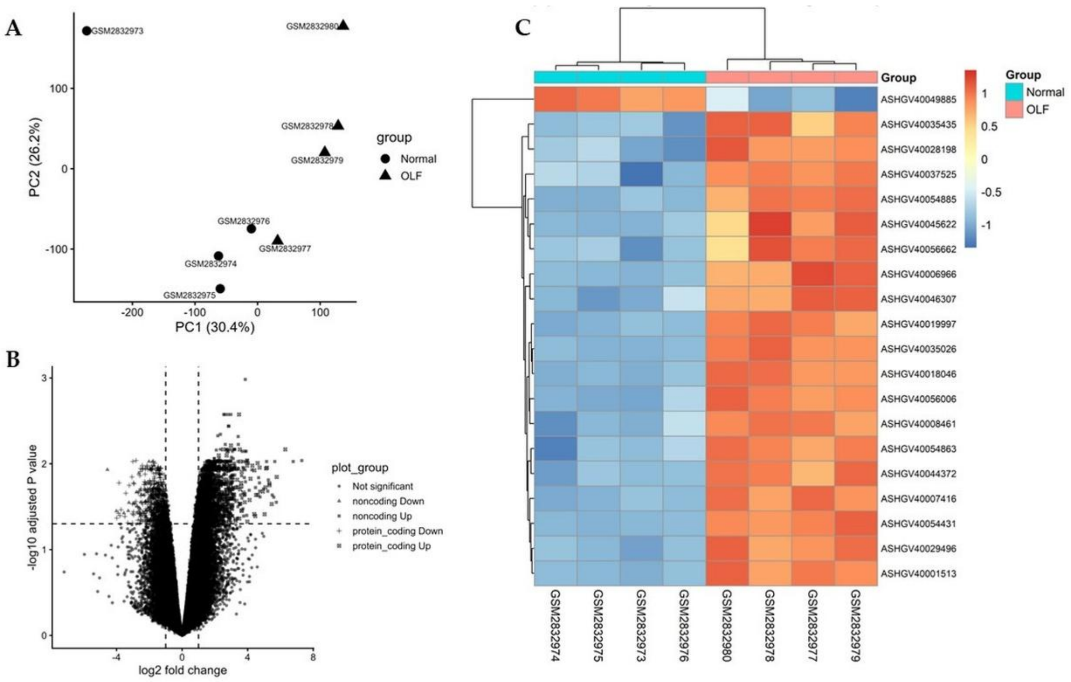

Figure S8. Supportive transcript-level analysis of GSE106253. (A) Principal component analysis of GSE106253 samples using the normalized transcript-level expression matrix. The dataset included 4

normal ligamentum flavum samples and 4 ossified ligamentum flavum samples. (B) Volcano plot of transcript-level differential expression between ossified and normal ligamentum flavum tissues. Differential expression was assessed at the transcript-feature level, and protein-coding and noncoding transcript-level features are indicated separately. (C) Heatmap of the top differentially expressed protein-coding and noncoding transcript-level features. Expression values are shown as row-wise z-scores. Because gene-symbol annotation was not available in the GPL21827 annotation file, probe/transcript identifiers are displayed.

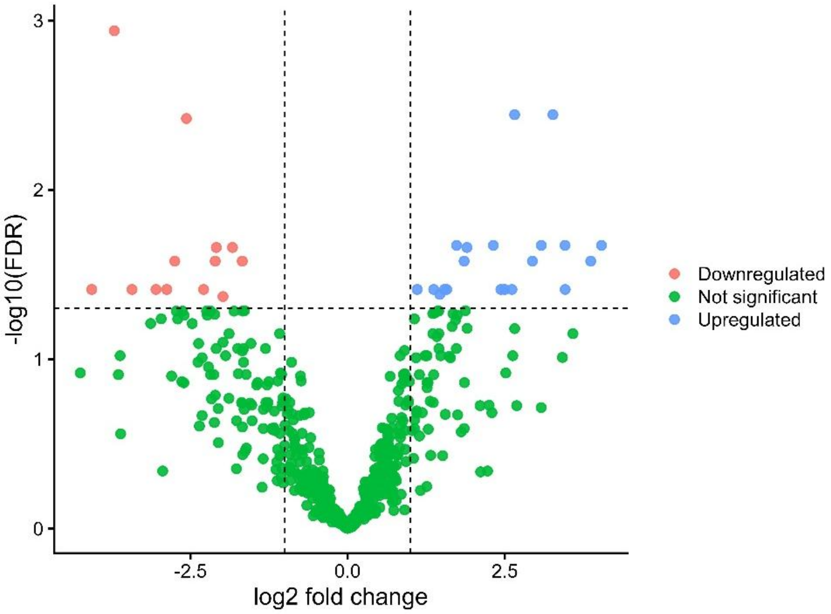

Figure S9. Volcano plot of differentially expressed microRNAs in the GSE106256 OLF miRNA-sequencing dataset. The volcano plot displays the distribution of 605 tested mature miRNAs according to log2 fold change (OLF versus normal ligamentum flavum) and −log10(FDR). Red dots represent significantly upregulated miRNAs and blue dots represent significantly downregulated miRNAs, defined by FDR < 0.05 and |log2 fold change| > 1. A total of 33 significant differentially expressed miRNAs were identified, comprising 20 upregulated and 13 downregulated miRNAs in OLF tissues.

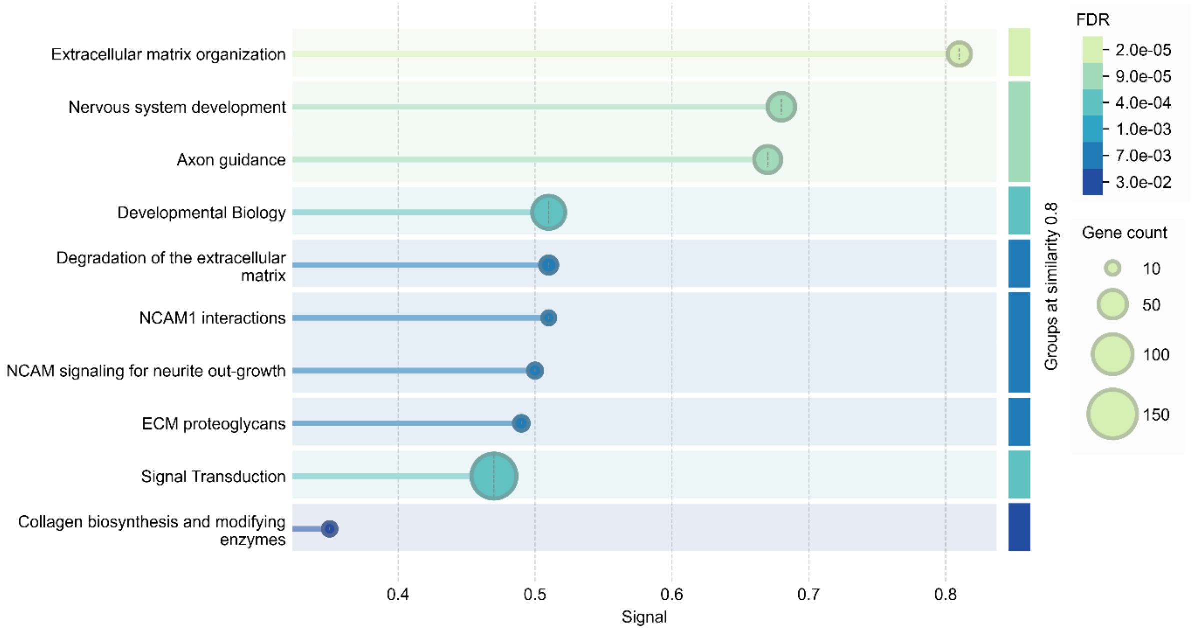

Figure S10. Reactome pathway enrichment analysis of the 651 inverse-regulated core genes identified from integrated mRNA–miRNA analysis. Bubble size represents the number of genes associated with each pathway, whereas color intensity corresponds to the false discovery rate (FDR). Extracellular matrix organization, nervous system development, axon guidance, developmental biology, extracellular matrix degradation, and related pathways were among the most significantly enriched Reactome terms.

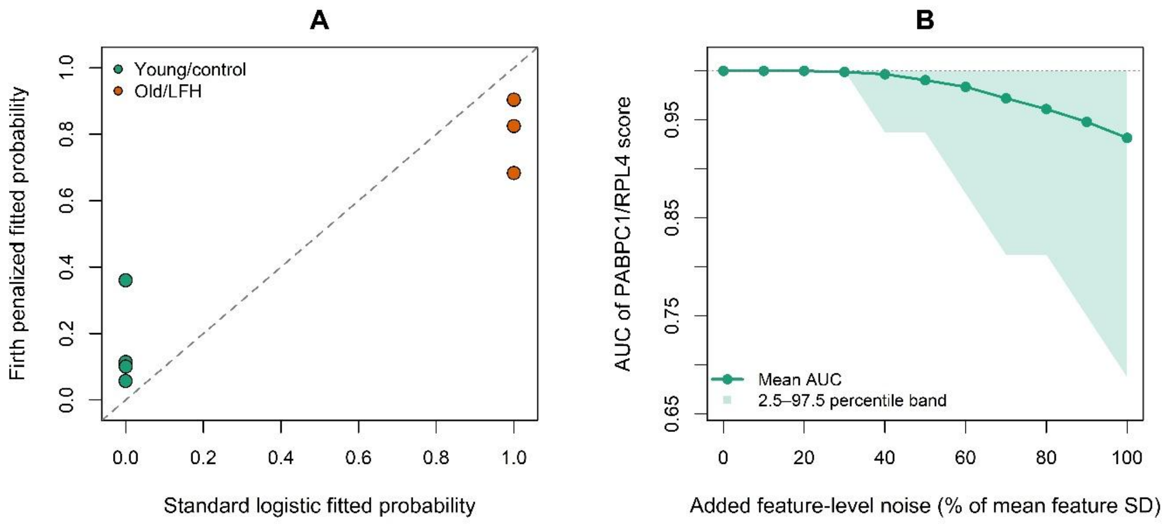

Figure S11. Separation-aware sensitivity analyses of the exploratory PABPC1–RPL4 candidate-feature score in GSE113212. (A) Comparison of standard maximum-likelihood logistic fitted probabilities with Firth penalized fitted probabilities for the two-gene PABPC1–RPL4 model. Firth penalization was used as a sensitivity approach to obtain finite fitted probabilities in the presence of complete-separation behavior. (B) Perturbation-based sensitivity analysis of the direction-aware PABPC1–RPL4 score after graded feature-level Gaussian perturbations from 0% to 100% of the mean feature standard deviation. The shaded area represents the 2.5th–97.5th percentile range across perturbation replicates and is provided as a noise-sensitivity summary rather than as a formal AUC confidence interval. These

analyses were performed to contextualize the apparent discovery-cohort discrimination of PABPC1 and RPL4 and do not constitute independent diagnostic validation.

Supplementary Tables

Table S1. Cell counts and quality-control metrics before and after filtering. Before-QC metrics were calculated from the raw GSE294458 count matrices, and after-QC metrics were calculated from the quality-filtered Seurat object. Retention percentage was calculated as the number of cells after QC divided by the number of cells before QC.

Table S1A. Cell recovery after quality control.

Group	Cells before QC	Cells after QC	Cells removed	Retention (%)
LDH_NLF	13033	12610	423	96.754
LSS_HLF	2655	2574	81	96.949
Total	15688	15184	504	96.787

Table S1B. Quality-control metrics before and after filtering.

Group	Metric	Before QC	After QC
LDH_NLF	Median nFeature_RNA	980.0	982.0
LDH_NLF	Median nCount_RNA	1607	1604
LDH_NLF	Median percent.mt	1.72	1.697
LDH_NLF	Mean percent.mt	2.713	2.215
LSS_HLF	Median nFeature_RNA	3058.0	3026.0
LSS_HLF	Median nCount_RNA	9702	9570
LSS_HLF	Median percent.mt	0.43	0.427
LSS_HLF	Mean percent.mt	0.948	0.837
Total	Median nFeature_RNA	1291.5	1291.5
Total	Median nCount_RNA	2367	2360
Total	Median percent.mt	1.41	1.418
Total	Mean percent.mt	2.415	1.981

Table S2. Representative top marker genes for each Seurat cluster. Representative top marker genes identified for each Seurat cluster in the quality-filtered GSE294458 dataset are listed. Marker genes were ranked according to average log2 fold change from the positive cluster marker analysis and are presented as a concise summary to support marker-based cluster interpretation.

Cluster	Top marker genes
0	*INHBB, THSD7A, FAM110D, PLCXD3, ADAMTS9, ARHGEF15, FAM107A, ACKR1, SHANK3, SNTG2*
1	*ASPN, CYS1, FIBIN, OGN, PART1, LRRC15, CCN5, GDF10, FNDC1, COL3A1*
2	*FCN1, LYZ, S100A9, AIF1, IFI30, SLC11A1, S100A8, VCAN, FCER1G, C1orf162*
3	*NELL2, THEMIS, CAMK4, GZMK, TC2N, CD3D, IL7R, LINC01934, TRAC, BCL11B*
4	*TPM2, MT1A, ACTA2, MYL9, C11orf96, TAGLN, MEDAG, TNFRSF12A, CTSC, ANGPTL4*
5	*GNLY, KLRD1, CD247, CCL5, NKG7, AOAH, SYTL3, SKAP1, PPP2R5C, FYN*
6	*SERPINB2, CLEC5A, EREG, IL10, CD300E, SIGLEC12, HPSE, SIGLEC10, AC025580.2, CCL20*
7	*TRIM10, KEL, SPTA1, GFI1B, CA1, MYL4, SPTB, SLC25A21, KCNH2, HEMGN*
8	*ADGRG3, PADI4, CSF3R, S100P, S100A12, GCA, G0S2, AQP9, SAMSN1, PAK1*
9	*FCRL1, PAX5, MS4A1, TCL1A, LINC02397, RHEX, BANK1, BLK, COL19A1, NIBAN3*
10	*MMP3, TNFRSF11B, HAPLN1, SERPINA3, SERPINE2, KCNMA1, MT1E, FN1, CILP, FMOD*
11	*FOLR2, LILRB5, C1QA, C1QC, F13A1, C1QB, MRC1, FCGR2B, FPR3, ME1*
12	*CYP4B1, PLA2G2A, MFAP5, IGF1, PRG4, DCLK1, SCARA5, PTX3, APOD, NEGR1*
13	*RERGL, RGS5, PLN, MYOCD, ADGRL3, ADRA2A, ACTA2, MUSTN1, LRRC10B, CDH6*
14	*PTCHD4, CD34, AC104211.2, TM4SF18, AGRN, EMCN, TSPAN18, CAVIN2, IL33, MEOX1*
15	*PRTN3, CTSG, AZU1, ELANE, DEFA4, MPO, MS4A3, RNASE2, ATP8B4, TYMS*
16	*PMEL, GPNMB, CALU, CD63, MDH2, BRI3, TRAM1, MT-CO3, ZC3H13, DBI*
17	*SH2D1B, KLRF1, KIR2DL4, S1PR5, LINGO2, CLIC3, LINC00299, CX3CR1, GZMB, TMIGD2*
18	*IGHGP, IGHG3, IGLC1, IGHG4, IGLC3, IGHG1, MZB1, IGHA1, IGKC, DERL3*
19	*WIF1, KLK4, BGLAP, AC062004.1, KLK1, WDR86, INSC, LINC01436, CHL1, SPP1*
20	*IFIT1B, TMCC2, HEPACAM2, LINC00570, LINC02772, TRIM58, SLFN14, AC130456.3, AC079804.3, AC123912.4*

Table S3. Selected biologically relevant marker genes supporting cluster interpretation. Selected biologically relevant marker genes supporting cluster interpretation in the quality-filtered GSE294458 dataset are listed. Genes were selected from the positive cluster marker output using statistical and biological criteria, including adjusted p value < 0.05, average log2 fold change > 1, expression in at least 20% of cells within the target cluster, and higher expression in the target cluster than in other clusters. The selected marker panel was focused on stromal, fibroblast, extracellular matrix, myofibroblast/contractile, endothelial, immune, erythroid, granulocytic, inflammatory, and fibrosis-related genes. Clusters without selected genes meeting these criteria are not shown.

Cluster	Selected biologically relevant marker genes
0	*ADAMTS9, ACKR1, CLDN5, VWF, ESAM, FLT1, PECAM1, MCAM, RAMP2, PLVAP*
1	*ASPN, FIBIN, OGN, LRRC15, COL3A1, COL1A1, COL1A2, MXRA5, COMP, OMD*
2	*FCN1, LYZ, S100A9, AIF1, S100A8, CD163, TYROBP, CTSS, S100A12*
3	*GZMK, CD3D, IL7R, TRAC, CD8B, CD8A, CD3E, CCL5, NKG7, KLRD1*
4	*TPM2, ACTA2, MYL9, TAGLN, CCL2, SERPINE1, TNC, PRG4*
5	*GNLY, KLRD1, CCL5, NKG7, IL7R*
6	*CCL20, IL1B, CD163, CXCL8, AIF1, TYROBP, FCN1, CD68, CTSS, MS4A7*
7	*GYPA, KLF1, SLC4A1*
8	*CSF3R, S100A12, CXCL8, S100A8, S100A9*
9	*PAX5, MS4A1, BANK1, CD79A, CD79B*
10	*MMP3, TNFRSF11B, SERPINA3, FN1, CILP, FMOD, PRELP, COL6A3, LUM, COMP*
11	*FOLR2, LILRB5, C1QA, C1QC, C1QB, MRC1, MS4A7, CD68, AIF1, CD163*
12	*MFAP5, PRG4, APOD, SERPINA3, PDGFRA, COL6A3, SERPINE1, ADAMTS4, CCL2, COL6A2*
13	*RGS5, PLN, ACTA2, MUSTN1, CNN1, MYH11, NOTCH3, MYL9, TPM2, TAGLN*
14	*CD34, EMCN, PLVAP, KDR, RAMP2, VWF, PECAM1, ESAM, FLT1, ENG*
15	*PRTN3, CTSG, AZU1, ELANE, MPO, LYZ*
17	*KLRF1, GZMB, GNLY, PRF1, NKG7, FCGR3A, KLRD1, CCL5*
18	*IGHG3, IGLC1, IGHG4, IGHG1, MZB1, IGKC, JCHAIN, CD79A*
19	*WIF1, SPP1, PDGFRA, OMD, TNC, PDGFRB, CTSK, COL1A2, COL1A1*
20	*SLC4A1, GYPA, KLF1*

Table S4. Cluster-wise cell counts and proportions by specimen. Cluster-level cell counts and within-group proportions for LDH_NLF and LSS_HLF samples after quality filtering are summarized. Percentages indicate the proportion of cells assigned to each Seurat cluster within the corresponding group. These data support the cluster-composition analysis of the GSE294458 single-cell dataset.

Cluster	LDH_NLF, n (%)	LSS_HLF, n (%)
0	2072 (16.431)	47 (1.826)
1	47 (0.373)	1381 (53.652)
2	1219 (9.667)	4 (0.155)
3	1039 (8.239)	52 (2.020)
4	1066 (8.454)	4 (0.155)
5	934 (7.407)	10 (0.389)
6	773 (6.130)	22 (0.855)
7	686 (5.440)	62 (2.409)
8	714 (5.662)	2 (0.078)
9	645 (5.115)	20 (0.777)
10	579 (4.592)	85 (3.302)
11	447 (3.545)	158 (6.138)
12	579 (4.592)	2 (0.078)
13	387 (3.069)	74 (2.875)
14	33 (0.262)	399 (15.501)
15	421 (3.339)	2 (0.078)
16	365 (2.895)	9 (0.350)
17	300 (2.379)	8 (0.311)
18	273 (2.165)	26 (1.010)
19	30 (0.238)	133 (5.167)
20	1 (0.008)	74 (2.875)
Total	12610 (100.000)	2574 (100.000)

Table S5. Significant differentially expressed genes in GSE113212. Differential expression analysis was performed for the Old versus Young ligamentum flavum comparison in GSE113212. A total of 35,789 probes/transcripts were analyzed. Significant differentially expressed genes were defined as genes with Benjamini–Hochberg adjusted p value < 0.05 and absolute logFC > 1. Positive logFC indicates higher expression in Old ligamentum flavum samples compared with Young samples, whereas negative logFC indicates lower expression in Old ligamentum flavum samples. Overall, 9 significant differentially expressed genes were identified, including 7 upregulated and 2 downregulated genes in Old ligamentum flavum samples.

Gene symbol	Probe ID	logFC	AveExpr	t	p value	Adjusted p value	B statistic	Regulation
BMPR1B	A_24_P63380	2.1200	$-2.775558 \times 10^{-17}$	12.962878	1.28×10^{-6}	0.046114	5.172997	Upregulated
CHRM3	A_23_P401472	2.2500	$-2.775558 \times 10^{-17}$	11.502560	3.18×10^{-6}	0.049751	4.568103	Upregulated
TGFB1	A_24_P79054	1.6200	$-1.387779 \times 10^{-17}$	11.090838	4.19×10^{-6}	0.049751	4.374422	Upregulated
LOC283404	A_33_P3606692	−2.2175	1.250000×10^{-3}	−10.579824	5.96×10^{-6}	0.049751	4.117935	Downregulated
MYADML	A_33_P3422923	−4.0750	2.775558×10^{-17}	−10.109930	8.35×10^{-6}	0.049751	3.865088	Downregulated
COL13A1	A_23_P1331	1.5925	1.250000×10^{-3}	10.096471	8.43×10^{-6}	0.049751	3.857592	Upregulated
MDM2	A_23_P502750	1.2650	1.387779×10^{-17}	9.740256	1.10×10^{-5}	0.049751	3.653831	Upregulated
CHST10	A_33_P3277198	1.7250	4.163336×10^{-17}	9.721413	1.12×10^{-5}	0.049751	3.642758	Upregulated
ULBP1	A_33_P3422802	3.0750	1.387779×10^{-17}	9.571172	1.25×10^{-5}	0.049751	3.553376	Upregulated

Table S6. Upregulated DEGs in Old ligamentum flavum samples. This table lists the significantly upregulated differentially expressed genes in Old ligamentum flavum samples compared with Young samples in GSE113212. Upregulated genes were defined as genes with Benjamini–Hochberg adjusted p value < 0.05 and logFC > 1. Positive logFC indicates higher expression in Old ligamentum flavum samples.

Gene symbol	Probe ID	logFC	AveExpr	t	P value	Adjusted P value	B statistic	Regulation
BMPR1B	A_24_P63380	2.1200	$-2.775558 \times 10^{-17}$	12.962878	1.28×10^{-6}	0.046114	5.172997	Upregulated
CHRM3	A_23_P401472	2.2500	$-2.775558 \times 10^{-17}$	11.502560	3.18×10^{-6}	0.049751	4.568103	Upregulated
TGFB1	A_24_P79054	1.6200	$-1.387779 \times 10^{-17}$	11.090838	4.19×10^{-6}	0.049751	4.374422	Upregulated
COL13A1	A_23_P1331	1.5925	1.250000×10^{-3}	10.096471	8.43×10^{-6}	0.049751	3.857592	Upregulated
MDM2	A_23_P502750	1.2650	1.387779×10^{-17}	9.740256	1.10×10^{-5}	0.049751	3.653831	Upregulated
CHST10	A_33_P3277198	1.7250	4.163336×10^{-17}	9.721413	1.12×10^{-5}	0.049751	3.642758	Upregulated
ULBP1	A_33_P3422802	3.0750	1.387779×10^{-17}	9.571172	1.25×10^{-5}	0.049751	3.553376	Upregulated

Table S7. Downregulated DEGs in Old ligamentum flavum samples. This table lists the significantly downregulated differentially expressed genes in Old ligamentum flavum samples compared with Young samples in GSE113212. Downregulated genes were defined as genes with Benjamini–Hochberg adjusted *p* value < 0.05 and logFC < −1. Negative logFC indicates lower expression in Old ligamentum flavum samples compared with Young samples.

Gene symbol	Probe ID	logFC	AveExpr	t	P value	Adjusted P value	B statistic	Regulation
LOC283404	A_33_P3606692	-2.2175	1.250000×10^{-3}	-10.579824	5.96×10^{-6}	0.049751	4.117935	Downregulated
MYADML	A_33_P3422923	-4.0750	2.775558×10^{-17}	-10.109930	8.35×10^{-6}	0.049751	3.865088	Downregulated

Table S8. Exploratory cell-level differential-expression summary for the LSS_HLF and LDH_NLF specimens in GSE294458. Significant differentially expressed genes identified in the exploratory all-cell comparison between LSS_HLF and LDH_NLF cells are summarized. Significance was defined as adjusted *p* value < 0.05. Positive average log2 fold change indicates higher expression in LSS_HLF cells, whereas negative average log2 fold change indicates higher expression in LDH_NLF cells. Representative top upregulated and downregulated genes are shown. Adjusted *p* values are shown using powers of ten; values below the numerical display limit are reported as $<1 \times 10^{-300}$. Because GSE294458 contains one specimen per condition, these cell-level differential-expression results represent sample-associated observations and do not support donor-level inference.

A. Summary of significant differentially expressed genes

Comparison	Significant DEGs	Upregulated in LSS_HLF	Downregulated in LSS_HLF
LSS_HLF vs LDH_NLF	5781	2753	3028

B. Representative top upregulated genes in LSS_HLF

Gene	avg_log2FC	pct.1 LSS_HLF	pct.2 LDH_NLF	Adjusted *p* value
XIST	15.141	0.675	0.000	$<1 \times 10^{-300}$
AC092958.1	6.611	0.113	0.001	2.15×10^{-297}
SOX15	6.013	0.102	0.001	1.14×10^{-257}
ASPN	5.919	0.555	0.038	$<1 \times 10^{-300}$
LRRC15	5.809	0.222	0.004	$<1 \times 10^{-300}$
TSPAN11	5.808	0.131	0.002	$<1 \times 10^{-300}$
CYS1	5.752	0.197	0.002	$<1 \times 10^{-300}$
HOXC10	5.745	0.265	0.005	$<1 \times 10^{-300}$
PTPRD-AS1	5.742	0.137	0.002	$<1 \times 10^{-300}$
MXRA5	5.733	0.359	0.009	$<1 \times 10^{-300}$

GDF10	5.609	0.220	0.005	$<1 \times 10^{-300}$
PART1	5.600	0.187	0.003	$<1 \times 10^{-300}$
DLX6	5.563	0.102	0.002	1.08×10^{-254}
CCN5	5.542	0.274	0.011	$<1 \times 10^{-300}$
FNDC1	5.500	0.253	0.007	$<1 \times 10^{-300}$
AC092957.1	5.495	0.361	0.011	$<1 \times 10^{-300}$
LINC01139	5.460	0.158	0.002	$<1 \times 10^{-300}$
OGN	5.245	0.539	0.043	$<1 \times 10^{-300}$
NDNF	5.203	0.102	0.004	2.30×10^{-223}
FIBIN	5.202	0.447	0.012	$<1 \times 10^{-300}$

C. Representative top downregulated genes in LSS_HLF

Gene	avg_log2FC	pct.1 LSS_HLF	pct.2 LDH_NLF	Adjusted *p* value
RPS4Y1	-12.943	0.000	0.453	$<1 \times 10^{-300}$
UTY	-11.863	0.000	0.273	2.81×10^{-191}
DDX3Y	-11.428	0.000	0.241	1.01×10^{-163}
USP9Y	-11.099	0.000	0.183	1.76×10^{-117}
LINC00278	-11.049	0.000	0.128	1.36×10^{-77}
PRKY	-10.974	0.000	0.171	3.49×10^{-108}
EIF1AY	-10.628	0.000	0.162	5.64×10^{-102}
IL1R2	-7.194	0.011	0.172	2.69×10^{-95}
S100A12	-5.625	0.008	0.139	2.33×10^{-75}
AQP9	-5.296	0.009	0.104	4.71×10^{-51}
CSF3R	-5.028	0.026	0.124	4.82×10^{-46}
RIPOR2	-4.694	0.063	0.314	2.84×10^{-158}
S100A8	-4.546	0.027	0.310	2.16×10^{-190}
AOAH	-4.504	0.047	0.215	1.10×10^{-90}
ADAMTS4	-4.464	0.037	0.159	5.61×10^{-59}
ADAMTS9	-4.352	0.108	0.328	1.04×10^{-128}
S100A9	-4.271	0.037	0.353	1.28×10^{-221}
TXK	-4.236	0.023	0.114	3.69×10^{-42}
MNDA	-4.151	0.042	0.148	1.51×10^{-47}

MT1A	-4.145	0.182	0.256	1.24×10^{-26}

Table S9. Selected biologically relevant genes upregulated in LSS_HLF cells. Selected biologically relevant genes showing higher expression in LSS_HLF cells compared with LDH_NLF cells are listed. Genes were selected from the significant upregulated DEG output with emphasis on extracellular matrix organization, fibroblast/stromal activation, ligament matrix remodeling, and fibrosis-associated transcriptional features. Positive average log2 fold change indicates higher expression in LSS_HLF cells. Adjusted *p* values are shown using powers of ten; values below the numerical display limit are reported as $<1 \times 10^{-300}$. Because GSE294458 contains one specimen per condition, these results should be interpreted as exploratory, sample-associated observations rather than donor-level evidence.

Gene	avg_log2FC	pct.1 LSS_HLF	pct.2 LDH_NLF	Adjusted *p* value
ASPN	5.919	0.555	0.038	$<1 \times 10^{-300}$
LRRC15	5.809	0.222	0.004	$<1 \times 10^{-300}$
MXRA5	5.733	0.359	0.009	$<1 \times 10^{-300}$
GDF10	5.609	0.220	0.005	$<1 \times 10^{-300}$
CCN5	5.542	0.274	0.011	$<1 \times 10^{-300}$
FNDC1	5.500	0.253	0.007	$<1 \times 10^{-300}$
OGN	5.245	0.539	0.043	$<1 \times 10^{-300}$
NDNF	5.203	0.102	0.004	2.30×10^{-223}
FIBIN	5.202	0.447	0.012	$<1 \times 10^{-300}$
COL8A1	5.184	0.446	0.019	$<1 \times 10^{-300}$
COL1A2	5.153	0.662	0.058	$<1 \times 10^{-300}$
MXRA8	5.145	0.584	0.031	$<1 \times 10^{-300}$
COL1A1	5.103	0.530	0.031	$<1 \times 10^{-300}$
OMD	4.994	0.442	0.028	$<1 \times 10^{-300}$
ANGPTL2	4.948	0.484	0.031	$<1 \times 10^{-300}$
COL3A1	4.810	0.652	0.050	$<1 \times 10^{-300}$
CTSK	4.643	0.511	0.029	$<1 \times 10^{-300}$
C1QTNF3	4.601	0.313	0.016	$<1 \times 10^{-300}$
FGF10	4.250	0.148	0.006	$<1 \times 10^{-300}$
COMP	4.206	0.577	0.059	$<1 \times 10^{-300}$
LUM	4.127	0.736	0.128	$<1 \times 10^{-300}$
PRELP	3.849	0.635	0.078	$<1 \times 10^{-300}$

DCN	3.639	0.751	0.170	$<1 \times 10^{-300}$
CILP	3.410	0.434	0.049	$<1 \times 10^{-300}$
FMOD	3.237	0.551	0.064	$<1 \times 10^{-300}$
PDGFRA	2.687	0.387	0.033	$<1 \times 10^{-300}$
FN1	2.379	0.768	0.195	$<1 \times 10^{-300}$
COL6A2	2.349	0.683	0.165	$<1 \times 10^{-300}$
COL6A3	1.843	0.526	0.114	$<1 \times 10^{-300}$
HAPLN1	1.231	0.259	0.032	$<1 \times 10^{-300}$
TNFRSF11B	0.807	0.258	0.047	9.84×10^{-257}
TNC	0.509	0.430	0.134	6.60×10^{-233}

Table S10. Selected biologically relevant genes downregulated in LSS_HLF cells. Selected biologically relevant genes showing lower expression in LSS_HLF cells compared with LDH_NLF cells are listed. Negative average log2 fold change indicates relatively higher expression in LDH_NLF cells. Genes were selected from the significant downregulated DEG output with emphasis on immune, inflammatory, myeloid, lymphoid, matrix-remodeling, and cell-type-associated transcriptional features. Adjusted *p* values are shown using powers of ten. Because this dataset contains one sample per group, these results should be interpreted as exploratory.

Gene	avg_log2FC	pct.1 LSS_HLF	pct.2 LDH_NLF	Adjusted *p* value
IL1R2	-7.194	0.011	0.172	2.69×10^{-95}
S100A12	-5.625	0.008	0.139	2.33×10^{-75}
AQP9	-5.296	0.009	0.104	4.71×10^{-51}
CSF3R	-5.028	0.026	0.124	4.82×10^{-46}
RIPOR2	-4.694	0.063	0.314	2.84×10^{-158}
S100A8	-4.546	0.027	0.310	2.16×10^{-190}
AOAH	-4.504	0.047	0.215	1.10×10^{-90}
ADAMTS4	-4.464	0.037	0.159	5.61×10^{-59}
ADAMTS9	-4.352	0.108	0.328	1.04×10^{-128}
S100A9	-4.271	0.037	0.353	1.28×10^{-221}
TXK	-4.236	0.023	0.114	3.69×10^{-42}
MNDA	-4.151	0.042	0.148	1.51×10^{-47}
NKG7	-3.065	0.021	0.161	4.72×10^{-74}
KLRD1	-3.047	0.064	0.128	2.79×10^{-19}
SRGN	-2.885	0.348	0.634	2.22×10^{-260}

CD163	-2.770	0.072	0.203	5.54×10^{-57}
CCL5	-2.594	0.032	0.144	2.26×10^{-51}
LYZ	-2.515	0.080	0.258	1.37×10^{-87}
CTSS	-1.572	0.172	0.239	1.85×10^{-15}
TYROBP	-1.009	0.106	0.229	4.01×10^{-39}
AIF1	-0.980	0.086	0.154	5.69×10^{-15}
IGKC	-0.547	0.285	0.583	5.94×10^{-255}
PTPRC	-2.741	0.096	0.369	1.48×10^{-164}
ARHGAP15	-3.705	0.093	0.355	5.43×10^{-162}
PRKCB	-3.747	0.051	0.276	1.08×10^{-132}
CD53	-3.086	0.090	0.305	7.52×10^{-120}
ALOX5AP	-4.043	0.072	0.264	2.20×10^{-103}
IKZF1	-3.876	0.073	0.260	5.48×10^{-101}
SKAP1	-4.096	0.042	0.209	1.88×10^{-90}
IL7R	-3.572	0.021	0.177	1.98×10^{-86}
SLA	-3.761	0.047	0.211	2.25×10^{-86}
SAMSN1	-3.531	0.064	0.232	2.95×10^{-83}

Table S11. Quality-control summary of GSE267819 after filtering. Quality-control metrics for the GSE267819 ligamentum flavum single-cell RNA-seq dataset after filtering are summarized by sample. The table reports the number of retained cells, median and mean numbers of detected genes per cell (nFeature_RNA), median and mean UMI counts per cell (nCount_RNA), and median and mean mitochondrial transcript percentage (percent.mt). After quality filtering, 18,470 cells were retained in total, including 1,914 LFH1 cells, 13,094 LFH2 cells, and 3,462 LFH3 cells. Because GSE267819 contains LFH samples only and lacks a non-LFH control group, these quality-control metrics are provided to document the processed single-cell dataset and were not used for LFH-versus-control differential expression analysis.

Sample	Cells after filtering	Median nFeature RNA	Mean nFeature RNA	Median nCount RNA	Mean nCount RNA	Median percent. mt	Mean percent. mt
LFH1	1,914	2,599.5	2,558.153	10,585.0	11,577.758	1.140	1.815
LFH2	13,094	2,110.0	2,144.538	7,312.0	8,150.549	1.400	2.070
LFH3	3,462	3,430.0	3,244.683	14,891.5	15,984.241	1.097	1.703

Table S12. Sample counts before balancing in GSE267819. Cell counts for each GSE267819 sample after quality filtering and before sample balancing are shown. The filtered object contained 1,914 LFH1 cells, 13,094 LFH2 cells, and 3,462 LFH3 cells. LFH2 therefore contributed a substantially larger fraction of cells than LFH1 and LFH3. To reduce the influence of this sample-size imbalance on visualization and marker-level assessment, a balanced object was generated by downsampling each sample to the same cell number. No LFH-versus-control comparison was performed because the dataset contains LFH samples only.

Sample	Cells before balancing
LFH1	1,914
LFH2	13,094
LFH3	3,462

Table S13. Balanced sample counts in GSE267819. Cell counts for each sample in the balanced GSE267819 object are shown. Each sample was downsampled to 1,914 cells, yielding a balanced object of 5,742 cells in total. This balanced object was used for balanced RNA UMAP visualization, sample-distribution assessment, cluster-level summary tables, and canonical marker-level analysis. The balancing procedure was used only to support sample-balanced visualization and marker assessment, not to infer disease-versus-control differential expression.

Sample	Balanced cell count
LFH1	1,914
LFH2	1,914
LFH3	1,914

Table S14. Balanced Seurat cluster counts in GSE267819. Cell counts for each Seurat cluster in the balanced GSE267819 object are summarized. The balanced object contained 11 Seurat clusters, numbered 0–10, with 5,742 cells in total. Cluster-level counts are provided to document the cellular structure of the balanced LFH-only single-cell dataset and to support the UMAP and canonical marker visualizations shown in Supplementary Figures S6 and S7. Because no non-LFH control sample is present in GSE267819, these clusters were interpreted at the marker-support level rather than as a primary disease-discovery atlas.

Seurat cluster	Balanced cluster cell count
0	1,282
1	857
2	819
3	798
4	740
5	494
6	258

7	166
8	157
9	126
10	45

Table S15. Sample fractions within balanced Seurat clusters in GSE267819. For each Seurat cluster in the balanced GSE267819 object, the contribution of LFH1, LFH2, and LFH3 cells is summarized as cell counts, within-cluster fractions, and within-cluster percentages. Fractions were calculated within each cluster and sum to 1 for each cluster. Sample–cluster combinations with zero cells are not shown as separate rows. This table is provided to document residual sample-associated structure in the balanced single-cell object. Therefore, GSE267819 was used as a supplementary supportive marker-level dataset rather than as a primary discovery atlas or LFH-versus-control differential expression dataset.

Seurat cluster	Sample	Cell count	Cluster total cells	Sample fraction within cluster	Sample percent within cluster
0	LFH1	40	1,282	0.031	3.120
0	LFH3	1,242	1,282	0.969	96.880
1	LFH1	802	857	0.936	93.582
1	LFH2	13	857	0.015	1.517
1	LFH3	42	857	0.049	4.901
2	LFH1	298	819	0.364	36.386
2	LFH2	373	819	0.455	45.543
2	LFH3	148	819	0.181	18.071
3	LFH1	110	798	0.138	13.784
3	LFH2	539	798	0.675	67.544
3	LFH3	149	798	0.187	18.672
4	LFH1	289	740	0.391	39.054
4	LFH2	329	740	0.445	44.459
4	LFH3	122	740	0.165	16.486
5	LFH1	41	494	0.083	8.300
5	LFH2	421	494	0.852	85.223
5	LFH3	32	494	0.065	6.478
6	LFH1	81	258	0.314	31.395

6	LFH2	61	258	0.236	23.643
6	LFH3	116	258	0.450	44.961
7	LFH1	66	166	0.398	39.759
7	LFH2	49	166	0.295	29.518
7	LFH3	51	166	0.307	30.723
8	LFH1	99	157	0.631	63.057
8	LFH2	57	157	0.363	36.306
8	LFH3	1	157	0.006	0.637
9	LFH1	44	126	0.349	34.921
9	LFH2	71	126	0.563	56.349
9	LFH3	11	126	0.087	8.730
10	LFH1	44	45	0.978	97.778
10	LFH2	1	45	0.022	2.222

Table S16. Canonical marker gene presence in GSE267819. Presence of the canonical marker genes used for marker-level assessment of the balanced GSE267819 single-cell RNA-seq object is summarized. All 31 selected marker genes were detected in the object. The marker panel included fibroblast/extracellular matrix-associated genes (COL1A1, COL1A2, COL3A1, DCN, LUM, FBLN1), myofibroblast/contractile genes (ACTA2, TAGLN, MYL9), endothelial genes (PECAM1, VWF, KDR, CLDN5), pericyte/smooth-muscle-associated genes (RGS5, PDGFRB, MCAM), immune and myeloid genes (PTPRC, LYZ, CD68, C1QA, C1QB), lymphoid genes (CD3D, CD3E, MS4A1, CD79A), mast-cell-associated genes (TPSAB1, TPSB2, KIT), and Schwann/neural-associated genes (S100B, MPZ, PLP1). Gene presence indicates that the marker was detected in the expression matrix and does not imply cluster-specific enrichment by itself.

Marker gene	Present in object
COL1A1	TRUE
COL1A2	TRUE
COL3A1	TRUE
DCN	TRUE
LUM	TRUE
FBLN1	TRUE
ACTA2	TRUE
TAGLN	TRUE
MYL9	TRUE

PECAM1	TRUE
VWF	TRUE
KDR	TRUE
CLDN5	TRUE
RGS5	TRUE
PDGFRB	TRUE
MCAM	TRUE
PTPRC	TRUE
LYZ	TRUE
CD68	TRUE
C1QA	TRUE
C1QB	TRUE
CD3D	TRUE
CD3E	TRUE
MS4A1	TRUE
CD79A	TRUE
TPSAB1	TRUE
TPSB2	TRUE
KIT	TRUE
S100B	TRUE
MPZ	TRUE
PLP1	TRUE

Table S17. Average canonical marker expression by Seurat cluster in GSE267819. Average normalized expression values of the 31 canonical marker genes are summarized across Seurat clusters 0–10 in the balanced GSE267819 object. Values are shown for the sample-balanced object containing 5,742 cells, with 1,914 cells from each LFH sample. This table supports the canonical marker DotPlot and FeaturePlot visualizations shown in Supplementary Figure S7. Values were rounded to three decimal places for display. Because GSE267819 contains LFH samples only and lacks a non-LFH control group, these marker-level expression summaries were used only for supplementary cellular-structure assessment and were not used for LFH-versus-control differential expression analysis.

Marker gene	Cluster 0	Cluster 1	Cluster 2	Cluster 3	Cluster 4	Cluster 5	Cluster 6	Cluster 7	Cluster 8	Cluster 9	Cluster 10
COL1A1	0.216	0.129	0.067	0.960	0.883	0.203	0.212	0.062	0.054	0.266	0.011
COL1A2	0.727	0.712	0.059	1.282	1.089	0.631	0.341	0.074	0.051	0.356	0.000
COL3A1	0.732	0.643	0.057	1.168	0.977	0.551	0.459	0.083	0.112	0.339	0.010
DCN	5.048	5.079	0.761	3.797	4.063	5.104	2.724	0.963	0.648	0.743	0.223
LUM	4.772	5.061	0.675	3.559	3.169	4.144	2.248	0.806	0.639	0.585	0.297
FBLN1	0.617	0.717	0.040	1.004	1.091	0.540	0.277	0.067	0.016	0.033	0.000
ACTA2	0.015	0.023	0.017	0.042	0.054	0.012	0.031	0.010	0.094	1.718	0.000
TAGLN	0.049	0.055	0.124	0.521	0.701	0.149	0.171	0.037	0.055	2.746	0.000
MYL9	0.117	0.040	0.141	0.258	0.288	0.090	0.032	0.024	0.011	2.144	0.000
PECAM1	0.003	0.011	0.775	0.023	0.015	0.015	0.070	0.137	0.060	0.017	0.000
VWF	0.002	0.000	0.513	0.021	0.015	0.012	0.002	0.025	0.004	0.000	0.000
KDR	0.003	0.001	0.796	0.020	0.012	0.007	0.009	0.015	0.012	0.005	0.013
CLDN5	0.025	0.017	1.011	0.038	0.020	0.028	0.086	0.022	0.000	0.009	0.000
RGS5	0.007	0.003	0.000	0.003	0.001	0.002	0.033	0.000	0.000	0.110	0.000
PDGFRB	0.586	0.369	0.013	0.377	0.263	0.206	0.304	0.014	0.012	0.471	0.008
MCAM	0.006	0.005	0.285	0.010	0.006	0.004	0.006	0.005	0.021	1.477	0.000
PTPRC	0.000	0.003	0.002	0.010	0.007	0.015	0.247	0.602	2.853	0.002	0.013
LYZ	0.004	0.003	0.001	0.005	0.003	0.011	0.245	1.221	0.007	0.015	0.000
CD68	0.492	0.339	0.036	0.258	0.188	0.291	0.263	2.120	0.014	0.030	0.018
C1QA	0.002	0.000	0.006	0.006	0.004	0.000	0.193	1.141	0.000	0.006	0.000
C1QB	0.006	0.002	0.007	0.004	0.003	0.008	0.172	1.084	0.000	0.016	0.000
CD3D	0.000	0.002	0.002	0.005	0.001	0.007	0.000	0.000	1.731	0.012	0.000
CD3E	0.000	0.001	0.001	0.003	0.002	0.004	0.000	0.000	1.302	0.000	0.000
MS4A1	0.000	0.000	0.000	0.000	0.000	0.001	0.112	0.000	0.007	0.000	0.000

CD79A	0.001	0.004	0.001	0.001	0.003	0.008	0.223	0.005	0.000	0.005	0.000
TPSAB1	0.000	0.000	0.000	0.000	0.000	0.000	0.017	0.000	0.000	0.000	0.000
TPSB2	0.001	0.000	0.000	0.000	0.010	0.004	0.066	0.001	0.000	0.000	0.000
KIT	0.001	0.004	0.067	0.002	0.008	0.004	0.019	0.005	0.009	0.009	0.016
S100B	0.293	0.174	0.011	0.043	0.168	0.363	0.035	0.003	0.003	0.000	0.010
MPZ	0.008	0.006	0.003	0.001	0.000	0.003	0.000	0.009	0.020	0.000	0.000
PLP1	0.002	0.000	0.000	0.008	0.013	0.000	0.000	0.000	0.000	0.000	0.000

Table S18. Significant differentially expressed microRNAs in GSE106256. Significant DEMs identified in OLF tissues compared with normal ligamentum flavum tissues are listed. Differential expression was assessed using mature miRNA counts after expression filtering. Significance was defined as FDR < 0.05 and |log2FC| > 1.

miRNA	logFC	AveExpr	t	P.Value	adj.P.Val	B
hsa-miR-novel-chr12_6330	-3.7126	6.0696	-9.6079	1.90×10^{-6}	0.001149	5.5192
hsa-miR-181a-5p	2.6578	11.5115	7.7770	1.30×10^{-5}	0.003595	3.6659
hsa-miR-653-5p	3.2668	3.6973	7.5057	1.78×10^{-5}	0.003595	3.3693
hsa-miR-381-3p	-2.5643	11.0892	-7.2192	2.51×10^{-5}	0.003790	3.0130
hsa-miR-191-5p	1.7335	10.2449	5.4518	0.0002568	0.02127	0.6648
hsa-miR-181a-3p	3.0814	6.6315	5.4052	0.0002746	0.02127	0.6809
hsa-miR-642a-5p	4.0393	-0.4109	5.3956	0.0002785	0.02127	0.7119
hsa-miR-708-5p	2.3198	6.8610	5.3606	0.0002929	0.02127	0.6173
hsa-miR-4683	3.4600	1.3898	5.3077	0.0003164	0.02127	0.6346
hsa-miR-136-5p	-1.8310	5.5465	-5.2108	0.0003645	0.02188	0.4176
hsa-miR-379-5p	-2.0887	7.9982	-5.1162	0.0004192	0.02188	0.2315
hsa-miR-181b-5p	1.8994	8.5533	5.0928	0.0004340	0.02188	0.1784
hsa-miR-215-5p	-2.1061	5.0291	-4.8172	0.0006574	0.02635	-0.1603
hsa-miR-337-5p	-2.7491	1.1601	-4.7938	0.0006813	0.02635	-0.0703
hsa-miR-653-3p	3.8686	0.9051	4.7926	0.0006826	0.02635	-0.0741
hsa-miR-181b-3p	2.9402	1.3146	4.7787	0.0006973	0.02635	-0.0913
hsa-miR-532-3p	1.8544	2.8518	4.7374	0.0007429	0.02635	-0.2097
hsa-miR-127-5p	-1.6755	6.0931	-4.7024	0.0007840	0.02635	-0.3572
hsa-miR-455-3p	2.4393	6.6382	4.2843	0.001513	0.03874	-1.0285

hsa-miR-377-3p	-2.2906	1.4335	-4.2454	0.001610	0.03874	-0.8755
hsa-miR-330-3p	1.3702	3.7472	4.2076	0.001711	0.03874	-1.0898
hsa-miR-375	2.6147	2.5443	4.1701	0.001818	0.03874	-1.0515
hsa-miR-130b-5p	2.4991	1.2094	4.1629	0.001839	0.03874	-0.9951
hsa-miR-331-3p	1.5774	4.6625	4.1584	0.001853	0.03874	-1.1976
hsa-miR-489-3p	3.4617	0.2104	4.1513	0.001874	0.03874	-0.9970
hsa-miR-376a-2-5p	-3.0487	0.7140	-4.1409	0.001906	0.03874	-1.0160
hsa-miR-455-5p	1.5418	9.3503	4.1328	0.001931	0.03874	-1.3633
hsa-miR-novel-chrX_47539	-2.8767	6.6340	-4.1232	0.001961	0.03874	-1.2973
hsa-miR-412-5p	-3.4300	0.0661	-4.1195	0.001973	0.03874	-1.0446
hsa-miR-1185-1-3p	-4.0705	0.2456	-4.1185	0.001976	0.03874	-1.0491
hsa-miR-23b-3p	1.1078	11.8976	4.1159	0.001985	0.03874	-1.4647
hsa-miR-novel-chr17_13457	1.4675	3.4230	4.0550	0.002192	0.04143	-1.3155
hsa-miR-134-5p	-1.9824	6.8575	-4.0181	0.002328	0.04267	-1.4670

Table S19. Complete list of the 651 inverse-regulated core genes identified through integrated mRNA–miRNA analysis in ligamentum flavum hypertrophy (LFH). These genes were retained after miRNA–mRNA inverse-direction filtering and were used for downstream GO-BP, KEGG, Reactome, and STRING analyses. Provided in Supplementary Excel Sheet S19.

Table S20. Gene Ontology Biological Process (GO-BP) enrichment analysis of the 651 inverse-regulated core genes. The table reports enriched GO-BP terms with term identifiers, descriptions, enrichment statistics, adjusted significance values, contributing gene IDs, and gene counts. Provided in Supplementary Excel Sheet S20.

Table S21. KEGG pathway enrichment analysis of the 651 inverse-regulated core genes. The table reports enriched KEGG pathways with pathway category, subcategory, pathway identifier, description, enrichment statistics, adjusted significance values, contributing gene IDs, and gene counts. Provided in Supplementary Excel Sheet S21.

Table S22. Reactome pathway enrichment analysis of the 651 inverse-regulated core genes. Reactome enrichment was performed using STRING. The table includes pathway-level enrichment statistics and complete matching gene symbols for all retained significant Reactome pathways. Provided in Supplementary Excel Sheet S22.

Table S23. Core miRNA–hub gene regulatory network identified in LFH. Inverse miRNA–mRNA interactions were intersected with CytoHubba-derived hub genes from the STRING protein–protein interaction network. The table lists inverse miRNA–hub gene interactions together with miRNA expression statistics, hub-gene expression statistics, and regulatory-direction information. Provided in Supplementary Excel Sheet S23.

Table S24. Complete miRNA–hub gene regulatory-network edge list. The table lists each inverse miRNA–hub gene interaction, including target gene, interacting miRNA, miRNA logFC, miRNA adjusted p value, miRNA direction, hub-gene logFC, and hub-gene direction. Provided in Supplementary Excel Sheet S24.

Table S25. Discovery-cohort discrimination, internal stability, and separation-aware sensitivity analyses of the exploratory PABPC1–RPL4 candidate-feature score in GSE113212. Provided in Supplementary Excel Sheet S25.

Table S26. Cross-cohort comparison of LASSO-selected genes identified in GSE113212 and evaluated in GSE294458. Mean expression differences in GSE294458 were calculated as LSS_HLF minus LDH_NLF, and directional concordance was assessed relative to the GSE113212 bulk transcriptomic results. Provided in Supplementary Excel Sheet S26.

Table S27. Immune and fibrosis signature-score column mapping used for GSE294458 single-cell signature analysis. The table maps each predefined signature to the corresponding score column used in downstream analyses. Provided in Supplementary Excel Sheet S27.

Table S28. Descriptive summary of immune and fibrosis signature scores in the GSE294458 single-cell dataset. Cell-level mean and median signature scores are summarized separately for the LDH_NLF and LSS_HLF specimens. Provided in Supplementary Excel Sheet S28.

Table S29. Exploratory cell-level comparison of immune and fibrosis signature scores between the LDH_NLF and LSS_HLF specimens in GSE294458. The table reports specimen-specific median scores, nominal P values, and FDR-adjusted values calculated across cells. Because GSE294458 contains one specimen per condition, these statistics are descriptive of the two specimens and do not support donor-level inference. Provided in Supplementary Excel Sheet S29.

Table S30. Prioritization of candidate miRNA regulators based on hub-gene targeting capacity. Candidate miRNAs were ranked according to the number of regulated hub-gene targets within the integrated miRNA–hub gene regulatory network. Provided in Supplementary Excel Sheet S30.

Table S31. KEGG gene set enrichment analysis (GSEA) results from the ranked GSE113212 bulk transcriptomic dataset. The table reports KEGG gene sets with enrichment score, normalized enrichment score, significance values, leading-edge information, core-enrichment genes, and log2 error estimates. Provided in Supplementary Excel Sheet S31.

Table S32. Gene Ontology Biological Process (GO-BP) gene set enrichment analysis (GSEA) results from the ranked GSE113212 bulk transcriptomic dataset. The table reports GO-BP gene sets with enrichment score, normalized enrichment score, significance values, leading-edge information, core-enrichment genes, and log2 error estimates. Provided in Supplementary Excel Sheet S32.

Table S33A. Sample-level fibrosis, proteoglycan/glycosaminoglycan (GAG), immune-inflammatory, and antigen-presentation signature scores calculated from the GSE113212 bulk transcriptomic dataset and used for the analyses presented in Figure 16. Signature scores were calculated as the mean gene-wise normalized expression of all available genes within each predefined signature set. Positive values indicate relatively higher signature activity, whereas negative values indicate relatively lower signature activity within the normalized dataset. Abbreviations: ECM, extracellular matrix; GAG, glycosaminoglycan.

Sample	Group	ECM Fibrosis	Proteoglycan GAG	Immune Inflammatory	Antigen Presentation
GSM3100386	Young	−0.3954770	−0.4280420	−0.5274520	−1.2696500
GSM3100387	Young	−1.0702140	−0.5837080	0.0798560	−0.5334500
GSM3100388	Young	−0.8071870	−0.2377080	0.2056250	−0.0560500
GSM3100389	Young	−0.2267710	−0.3127080	0.4067790	−0.1254500
GSM3100390	Old	1.0459920	−0.0240420	−0.5689900	1.2501500
GSM3100391	Old	0.7890190	0.6249580	−0.2239900	0.6619500
GSM3100392	Old	0.1259480	0.9342920	0.9652400	0.4629500
GSM3100393	Old	0.5386900	0.0269580	−0.3370670	−0.3904500

Table S33B. Spearman correlation analysis between LASSO-selected genes and fibrosis–immune signature scores in the GSE113212 bulk transcriptomic dataset. Correlation coefficients and corresponding p values were calculated using sample-level signature scores derived from the predefined ECM-fibrosis, proteoglycan/GAG, immune-inflammatory, and antigen-presentation gene signatures. These analyses were used for the correlation heatmap presented in Figure 16C.

Gene	Signature	Spearman rho	P value
PABPC1	ECM Fibrosis	−0.785714	0.020815
RPL4	ECM Fibrosis	−0.642857	0.085559
PABPC1	Proteoglycan GAG	−0.785714	0.020815
RPL4	Proteoglycan GAG	−0.833333	0.010176

Gene	Signature	Spearman rho	P value
PABPC1	Immune Inflammatory	0.333333	0.419817
RPL4	Immune Inflammatory	0.047619	0.910831
PABPC1	Antigen Presentation	−0.523810	0.182697
RPL4	Antigen Presentation	−0.738095	0.036612

Table S33C. Complete gene membership of the four remodeling signatures used for the signature-score analyses presented in Figure 16 and Table S33A. Each signature was assembled from canonical, literature-supported marker genes representative of the corresponding biological program. Only genes present in the GSE113212 expression matrix were included in signature-score calculation. A leave-one-gene-out sensitivity analysis, in which each signature score was recomputed after removing one constituent gene at a time, is summarized below each signature to demonstrate that no single gene disproportionately determined the signature score. Abbreviations: ECM, extracellular matrix; GAG, glycosaminoglycan.

Signature	Genes (n)	Gene symbols
ECM fibrosis	38	*ADAM12, COL1A1, COL1A2, COL3A1, COL4A1, COL4A2, COL5A2, COL6A1, COL6A3, COL8A1, COL11A1, COL12A1, COL13A1, COL14A1, CTGF, CTHRC1, DCN, EMILIN2, FMOD, HSPG2, LAMB2, LOX, LOXL1, LOXL2, LUM, MMP2, MMP9, MMP14, PDGFRB, POSTN, SERPINE1, SPP1, TGFB1, THBS1, THBS2, TIMP2, TNC, VCAN*
Proteoglycan/GAG	5	*B3GAT1, B3GAT3, CHST10, CSGALNACT2, DCN*
Immune-inflammatory	13	*BIRC3, CXCL9, CXCL10, IFNG, IL1R1, IL1RN, IL6, IRF1, NLRP3, PTGS2, S100A8, TLR2, TNFSF13B*
Antigen presentation	5	*HLA-DMA, HLA-DMB, HLA-DPA1, HLA-DPB1, HLA-DRA*

Leave-one-gene-out sensitivity summary. For each signature, the table reports the range of Pearson correlations between the reduced-signature score and the full-signature score, and the range of maximum absolute deviations, across all single-gene removals.

Signature	Pearson correlation (min–max)	Maximum absolute deviation (min–max)
ECM fibrosis	0.987–0.995	0.096–0.172
Proteoglycan/GAG	0.800–0.974	0.190–0.673
Immune-inflammatory	0.958–0.990	0.132–0.242

Signature	Pearson correlation (min–max)	Maximum absolute deviation (min–max)
Antigen presentation	0.987–0.991	0.155–0.184

Table S34. Prioritization of candidate miRNA regulators linked to fibrotic–immune remodeling. Candidate regulatory miRNAs were prioritized by integrating the total number of targeted hub genes with their overlap with Figure 16-associated LASSO genes, including PABPC1 and RPL4. The table reports each miRNA, the total number of hub-gene targets, the complete target-gene list, Figure 16-associated target genes, the number of Figure 16-associated targets, miRNA logFC, adjusted *p* value, and miRNA dysregulation direction.

miRNA	Total hub-gene targets	Target genes	Figure 16-associated targets	Number of Figure 16-associated targets	miRNA logFC	Adjusted *p* value	miRNA direction in GSE106256 OLF
hsa-miR-708-5p	2	*PABPC1; RPL4*	PABPC1; RPL4	2	2.32	0.0213	Up_in_OLF
hsa-miR-23b-3p	8	*CD74; EEF1A1; PABPC1; RPL19; RPL3; RPL5; RPS27A; RPS6*	PABPC1	1	1.11	0.0387	Up_in_OLF
hsa-miR-191-5p	6	*CD74; PABPC1; RPL5; RPL9; RPS6; SMKR1*	PABPC1	1	1.73	0.0213	Up_in_OLF
hsa-miR-181b-5p	5	*PABPC1; RPL19; RPL5; RPS20; RPS27A*	PABPC1	1	1.90	0.0219	Up_in_OLF
hsa-miR-455-3p	5	*EEF1A1; PABPC1; RPL19; RPS27; RPS6*	PABPC1	1	2.44	0.0387	Up_in_OLF
hsa-miR-181a-5p	4	*PABPC1; RPL5;*	PABPC1	1	2.66	0.00360	Up_in_OLF

		RPS27; RPS27A					
hsa-miR-455-5p	4	*PABPC1; RPL19; RPL5; RPS6*	PABPC1	1	1.54	0.0387	Up_in_OLF
hsa-miR-130b-5p	3	*PABPC1; RPL19; RPS6*	PABPC1	1	2.50	0.0387	Up_in_OLF
hsa-miR-653-5p	2	*PABPC1; RPS27A*	PABPC1	1	3.27	0.00360	Up_in_OLF
hsa-miR-330-3p	1	*PABPC1*	PABPC1	1	1.37	0.0387	Up_in_OLF
hsa-miR-215-5p	6	*BUB1B; CEP55; DLGAP5; HMMR; KIF14; KIF15*		0	−2.11	0.0264	Down_in_OLF
hsa-miR-134-5p	1	*TOP2A*		0	−1.98	0.0427	Down_in_OLF
hsa-miR-181a-3p	1	*RPL3*		0	3.08	0.0213	Up_in_OLF
hsa-miR-181b-3p	1	*RPS6*		0	2.94	0.0264	Up_in_OLF
hsa-miR-331-3p	1	*RPS27*		0	1.58	0.0387	Up_in_OLF

Table S35. miRNA–Figure 16 gene–signature links. The table summarizes inverse miRNA–hub gene interactions involving Figure 16-associated LASSO genes, PABPC1 and RPL4, and links each targeted gene to fibrosis-, proteoglycan/GAG-, and antigen-presentation–related signature associations identified in Figure 16. Columns include miRNA, target gene, miRNA logFC, miRNA adjusted *p* value, miRNA direction, target-gene logFC, target-gene direction, and linked signature associations.

miRNA	Target gene	miRNA logFC	miRNA adjusted *p* value	miRNA direction in GSE106256 OLF	Target-gene logFC	Target-gene direction	Linked signature associations
hsa-miR-130b-5p	*PABPC1*	2.50	0.0387	Up_in_OLF	−1.11	Down_gene	ECM_Fibrosis (rho=−0.79, P=0.0208); Proteoglycan_GAG (rho=−0.79, P=0.0208); Antigen_Presentation (rho=−0.52, P=0.1827)
hsa-miR-181a-5p	*PABPC1*	2.66	0.00360	Up_in_OLF	−1.11	Down_gene	ECM_Fibrosis (rho=−0.79, P=0.0208);

miRNA	Target gene	miRNA logFC	miRNA adjusted *p* value	miRNA direction in GSE106256 OLF	Target-gene logFC	Target-gene direction	Linked signature associations
							Proteoglycan_GAG (rho=−0.79, P=0.0208); Antigen_Presentation (rho=−0.52, P=0.1827)
hsa-miR-181b-5p	*PABPC1*	1.90	0.0219	Up_in_OLF	−1.11	Down_gene	ECM_Fibrosis (rho=−0.79, P=0.0208); Proteoglycan_GAG (rho=−0.79, P=0.0208); Antigen_Presentation (rho=−0.52, P=0.1827)
hsa-miR-191-5p	*PABPC1*	1.73	0.0213	Up_in_OLF	−1.11	Down_gene	ECM_Fibrosis (rho=−0.79, P=0.0208); Proteoglycan_GAG (rho=−0.79, P=0.0208); Antigen_Presentation (rho=−0.52, P=0.1827)
hsa-miR-23b-3p	*PABPC1*	1.11	0.0387	Up_in_OLF	−1.11	Down_gene	ECM_Fibrosis (rho=−0.79, P=0.0208); Proteoglycan_GAG (rho=−0.79, P=0.0208); Antigen_Presentation (rho=−0.52, P=0.1827)
hsa-miR-330-3p	*PABPC1*	1.37	0.0387	Up_in_OLF	−1.11	Down_gene	ECM_Fibrosis (rho=−0.79, P=0.0208); Proteoglycan_GAG (rho=−0.79, P=0.0208); Antigen_Presentation (rho=−0.52, P=0.1827)
hsa-miR-455-3p	*PABPC1*	2.44	0.0387	Up_in_OLF	−1.11	Down_gene	ECM_Fibrosis (rho=−0.79, P=0.0208); Proteoglycan_GAG (rho=−0.79, P=0.0208); Antigen_Presentation (rho=−0.52, P=0.1827)
hsa-miR-455-5p	*PABPC1*	1.54	0.0387	Up_in_OLF	−1.11	Down_gene	ECM_Fibrosis (rho=−0.79, P=0.0208); Proteoglycan_GAG (rho=−0.79, P=0.0208); Antigen_Presentation (rho=−0.52, P=0.1827)
hsa-miR-653-5p	*PABPC1*	3.27	0.00360	Up_in_OLF	−1.11	Down_gene	ECM_Fibrosis (rho=−0.79, P=0.0208); Proteoglycan_GAG (rho=−0.79, P=0.0208);

miRNA	Target gene	miRNA logFC	miRNA adjusted *p* value	miRNA direction in GSE106256 OLF	Target-gene logFC	Target-gene direction	Linked signature associations
							Antigen_Presentation (rho=−0.52, P=0.1827)
hsa-miR-708-5p	*PABPC1*	2.32	0.0213	Up_in_OLF	−1.11	Down_gene	ECM_Fibrosis (rho=−0.79, P=0.0208); Proteoglycan_GAG (rho=−0.79, P=0.0208); Antigen_Presentation (rho=−0.52, P=0.1827)
hsa-miR-708-5p	*RPL4*	2.32	0.0213	Up_in_OLF	−1.11	Down_gene	ECM_Fibrosis (rho=−0.64, P=0.0856); Proteoglycan_GAG (rho=−0.83, P=0.0102); Antigen_Presentation (rho=−0.74, P=0.0366)

Table S36. Drug–gene interactions of LFH-associated hub genes retrieved from the Drug–Gene Interaction Database (DGIdb). Hub genes were prioritized by cytoHubba maximal clique centrality from the PPI network of 650 mapped inverse-regulated core genes. Interaction scores represent DGIdb-native composite evidence scores; higher scores indicate greater aggregated database evidence. Regulatory status was retained as reported in the DGIdb output and was not reclassified post hoc. The niflumic acid (BUB1B) entry retains its DGIdb-registered status of Not Approved. Ribosomal proteins (RPL3, RPL4, RPL5, RPL9, RPL19, RPS6, RPS20, and RPS27A) shared a five-compound interaction profile and are presented as a unified pharmacologic-annotation class; the RPS6-specific sonolisib interaction is listed separately. For TOP2A, the highest-scoring approved entries are shown individually, whereas the remaining approved entries are summarized in the final TOP2A row. Abbreviations: DGIdb, Drug–Gene Interaction Database; ECM, extracellular matrix; GAG, glycosaminoglycan; LFH, ligamentum flavum hypertrophy; MIF, macrophage migration inhibitory factor; NSAID, non-steroidal anti-inflammatory drug; PKC, protein kinase C; PPI, protein–protein interaction; TOP2A, topoisomerase II alpha. Text colors are used solely to distinguish DGIdb-reported regulatory-status categories and do not indicate biological significance, effect size, or interaction strength.

Hub Gene	Drug / Compound	Regulatory Status	Drug Class / Indication	Interaction Score	Pharmacological Notes
EEF1A1	Sparsomycin	Investigational	Translation inhibitor (peptidyl transferase / 60S)	52.20	Highest composite score in dataset. Blocks EEF1A1–ribosome pretranslocation state;

Hub Gene	Drug / Compound	Regulatory Status	Drug Class / Indication	Interaction Score	Pharmacological Notes
					mechanistically aligned with KEGG GSEA ribosomal-pathway suppression in LFH.
EEF1A1	Tetradecanoylphorbol acetate (TPA)	Research use	PKC activator / pro-inflammatory agent	1.13	PKC-mediated upregulation of EEF1A1 expression; links translational elongation to pro-fibrotic inflammatory signaling.
CD74	Milatuzumab	Investigational	Anti-CD74 monoclonal antibody (antineoplastic)	41.76	Direct anti-CD74 immunotherapy; mechanistically coherent with MHC-II antigen-presentation and MIF-receptor remodeling signatures in LFH.
CD74	VU0240551	Research use	KCC2 inhibitor / chloride transporter modulator	10.44	Ion transport modulator; indirect immune relevance. Limited direct applicability to LFH fibrotic-immune context.
CD74	DIOA	Research use	Cl^- cotransport inhibitor	2.61	Non-specific chloride channel blocker; pharmacological significance in

Hub Gene	Drug / Compound	Regulatory Status	Drug Class / Indication	Interaction Score	Pharmacological Notes
					LFH context is speculative.
CD74	Platinum	Research use	DNA cross-linking agent	1.04	CD74 mediates cisplatin-resistance mechanisms via MIF-receptor and NF-κB signaling; supports immune-modulatory role.
CD74	Repotrectinib	Approved (oncology)	ROS1/TRK/ALK tyrosine kinase inhibitor	0.75	CD74–ROS1 oncogenic fusion is the approved repotrectinib target; independently validates CD74 as a pharmacologically relevant fusion partner.
HMMR	Hyaluronic acid	Approved (multiple indications)	Glycosaminoglycan / viscoelastic agent	4.35	HMMR (RHAMM) is the primary hyaluronan receptor. Most biologically direct pharmacological link to LFH GAG-remodeling axis identified in GO-BP and KEGG enrichment.
HMMR	Epirubicin	Approved (oncology)	Anthracycline antineoplastic	0.73	HMMR overexpression modulates

Hub Gene	Drug / Compound	Regulatory Status	Drug Class / Indication	Interaction Score	Pharmacological Notes
					anthracycline sensitivity in fibroproliferative conditions via ECM-associated proliferative programs.
HMMR	Cyclophosphamide anhydrous	Approved (oncology / immunology)	Alkylating antineoplastic / immunosuppressive	0.32	HMMR expression correlates with cyclophosphamide response; supports immunosuppressive targeting of fibroproliferative HMMR-expressing populations.
HMMR	Fluorouracil	Approved (oncology)	Antimetabolite antineoplastic	0.23	HMMR-associated proliferative programs modulate 5-FU sensitivity; mechanistic link to ECM-remodeling compartment.
PABPC1	CHEMBL585964	Investigational	Sigma receptor ligand (undisclosed structure)	6.53	Highest PABPC1 interaction score in dataset. Sigma receptor engagement may modulate stress-granule assembly and PABPC1-dependent mRNA stability programs.

Hub Gene	Drug / Compound	Regulatory Status	Drug Class / Indication	Interaction Score	Pharmacological Notes
PABPC1	CHEMBL592588 / 1,3-Ditolylguanidine	Research use	Sigma-1/sigma-2 receptor agonist	4.35	Sigma receptor agonist; modulates integrated-stress-response and translational reprogramming pathways involving poly(A)-binding proteins.
PABPC1	CHEMBL589711	Research use	Undisclosed small molecule	2.61	Pharmacological characterization incomplete; interaction is exploratory. No approved drug documented for PABPC1 in DGIdb.
RPS27	Empesertib	Investigational	WEE1 kinase inhibitor (antineoplastic)	2.49	WEE1 inhibition engages p53–RPS27 ribosomal-stress axis; cell-cycle checkpoint regulation intersects with ribosomal protein surveillance.
RPS27	BOS172722	Investigational	MPS1/TTK kinase inhibitor (antineoplastic)	1.24	Spindle-assembly checkpoint kinase inhibitor; converges on RPS27-associated ribosomal-stress and mitotic-checkpoint programs.

Hub Gene	Drug / Compound	Regulatory Status	Drug Class / Indication	Interaction Score	Pharmacological Notes
RPS27	Ataluren	Approved (rare disease)	PTC readthrough / translational modulator	0.09	Shared class-level ribosomal interaction (see ribosomal hub-gene cluster below).
BUB1B	3-Phenyl-CPP	Research use	CFTR/Cl⁻ channel modulator	8.70	Highest BUB1B interaction score. Ion channel modulation converges on mitotic-checkpoint signaling; mechanistic basis in LFH context remains speculative.
BUB1B	DIDS	Research use	Broad-spectrum anion transport inhibitor	3.48	Non-specific Cl⁻ channel blocker; indirect intersection with BUB1B-associated spindle-checkpoint signaling.
BUB1B	Niflumic acid	Not approved (research use)	COX inhibitor / Cl⁻ channel blocker (NSAID class)	1.34	NSAID-class compound in DGIdb with Not Approved status. BUB1B–COX pathway intersection is plausible but requires experimental validation in LFH tissue. Note: regulatory

Hub Gene	Drug / Compound	Regulatory Status	Drug Class / Indication	Interaction Score	Pharmacological Notes
					approval not confirmed in DGIdb dataset.
TOP2A	Pixantrone	Approved (oncology)	Anthracycline analogue / TOP2A poison	0.89	Direct catalytic TOP2A targeting; clinically validated anthracycline-class inhibitor.
TOP2A	Valrubicin	Approved (oncology)	Anthracycline / TOP2A poison	0.89	Direct TOP2A targeting; supports transcriptional-stress role of TOP2A in proliferating LFH fibroblast-like populations.
TOP2A	Etoposide	Approved (oncology)	Topoisomerase II inhibitor (epipodophyllotoxin)	0.41	Prototypical TOP2A poison; confirms TOP2A druggability in cell-cycle–active remodeling compartments.
TOP2A	Amsacrine	Approved (oncology)	Aminoacridine / DNA-intercalating TOP2A poison	0.39	Direct DNA-intercalating TOP2A inhibitor; well-characterised mechanism of action.
TOP2A	Dexrazoxane	Approved (cardioprotection)	Catalytic TOP2A inhibitor / cardioprotective agent	0.36	Only approved TOP2A catalytic (non-intercalating) inhibitor; non-cytotoxic

Hub Gene	Drug / Compound	Regulatory Status	Drug Class / Indication	Interaction Score	Pharmacological Notes
					mechanism potentially relevant to non-neoplastic fibroproliferative contexts.
TOP2A	Teniposide	Approved (oncology)	Epipodophyllotoxin / TOP2A inhibitor	0.30	Semi-synthetic TOP2A inhibitor; supports cell-cycle regulatory role of TOP2A in activated fibroblast-like LFH populations.
TOP2A	Etoposide phosphate	Approved (oncology)	Prodrug of etoposide / TOP2A inhibitor	0.45	Water-soluble etoposide prodrug; same mechanistic class as etoposide, included as distinct approved entity (DGIdb score: 0.446).
TOP2A	Daunorubicin citrate	Approved (oncology)	Anthracycline / TOP2A-intercalating agent	0.45	Approved anthracycline-class TOP2A-interacting agent; score 0.446 (TSV-verified).
TOP2A	Doxorubicin hydrochloride	Approved (oncology)	Anthracycline antineoplastic / TOP2A poison	0.03	Canonical anthracycline TOP2A poison; interaction score low in DGIdb composite weighting despite strong mechanistic evidence in

Hub Gene	Drug / Compound	Regulatory Status	Drug Class / Indication	Interaction Score	Pharmacological Notes
					literature.
TOP2A (30 approved total in DGIdb)	Additional approved agents (Cinoxacin, Mitoxantrone, Epirubicin, Idarubicin, Vincristine, Paclitaxel, Dactinomycin, Nalidixic acid, and others)	Approved (various indications)	Multiple drug classes (fluoroquinolones, anthracyclines, taxanes, vinca alkaloids)	0.004–0.45	21 additional approved drugs interact with TOP2A in DGIdb (total approved n = 30 out of 117 interactions). Score range reflects DGIdb composite evidence weighting across interaction types. Full list available in raw TSV data.
RPL3, RPL4, RPL5, RPL9, RPL19, RPS6, RPS20, RPS27A (ribosomal cluster, n = 8 genes)	Ataluren (PTC124)	Approved (rare disease)	Nonsense suppression / ribosomal readthrough agent	0.106–0.127 (8/8 genes)	Sole approved drug across all 8 ribosomal hub genes. Targets premature stop-codon readthrough via ribosomal P-site modulation. Class-level interaction uniformly documented; approved for Duchenne muscular dystrophy associated with nonsense mutations.
RPL3, RPL4, RPL5, RPL9,	Cycloheximide	Research use	60S ribosomal translocation inhibitor (eEF2 blocker)	0.092–0.110 (8/8 genes)	Gold-standard translational inhibitor; confirms

Hub Gene	Drug / Compound	Regulatory Status	Drug Class / Indication	Interaction Score	Pharmacological Notes
RPL19, RPS6, RPS20, RPS27A					pharmacological targetability of ribosomal hub-gene cluster as a translational regulatory module.
RPL3, RPL4, RPL5, RPL9, RPL19, RPS6, RPS20, RPS27A	MT-3724	Investigational	Ribosome-inactivating immunotoxin	0.107–0.129 (8/8 genes)	Engineered anti-CD20-saporin immunotoxin that inactivates ribosomes; mechanistically validates ribosomal protein complex as pharmacological target class.
RPL3, RPL4, RPL5, RPL9, RPL19, RPS6, RPS20, RPS27A	Exaluren	Investigational	PTC readthrough agent (ataluren analogue)	0.109–0.131 (8/8 genes)	Investigational ataluren-class translational modulator; identical class-level ribosomal profile to ataluren across all 8 hub genes.
RPL3, RPL4, RPL5, RPL9, RPL19, RPS6, RPS20, RPS27A	Dorlimomab aritox	Investigational	Anti-CD5 immunotoxin	0.109–0.131 (8/8 genes)	Ribosome-inactivating immunotoxin (anti-CD5–ricin A chain conjugate); class-level ribosomal interaction across all 8 hub genes consistent with ricin-A chain mechanism.

Hub Gene	Drug / Compound	Regulatory Status	Drug Class / Indication	Interaction Score	Pharmacological Notes
RPS6 (only)	Sonolisib	Investigational	PI3K inhibitor (antineoplastic)	0.48 (1/8 genes)	RPS6-specific interaction; mTORC1-mediated S6 kinase phosphorylates RPS6, linking PI3K–mTOR–S6 signaling axis to RPS6 pharmacological targeting.

Table S37. Database-supported annotation of LFH-associated miRNA–hub gene interactions. miRNA–target pairs identified within the integrated LFH regulatory framework were evaluated using the multiMiR integrated database (database version 2.4.0; updated 28 August 2024). The queried interaction set was derived from the miRNA–hub gene network and downstream analysis tables (Tables S23, S24, S34, and S35). For each miRNA–target pair, records were retrieved separately from curated miRNA–target interaction databases and computational prediction databases available through multiMiR. Database names, evidence descriptors, support categories, and related annotations were retained as reported by multiMiR. For presentation, entries were grouped according to source type as curated-database records, prediction-database records only, or no database support identified. Inclusion in a curated database was interpreted solely as database-level annotation and did not constitute LFH-specific experimental validation or functional confirmation in the present study. Abbreviations: LFH, ligamentum flavum hypertrophy; miRNA, microRNA. Provided in Supplementary Excel Sheet S37.

Table S38. ChEA 2022 transcription-factor enrichment analysis of CytoHubba MCC-prioritized hub genes associated with ligamentum flavum hypertrophy.

Transcription Factor / Dataset	Overlap	P-value	Adjusted P-value	Odds Ratio	Combined Score	Overlapping Genes
MYC 19030024 ChIP-ChIP MESCs Mouse	15/2842	1.44e-09	7.43e-07	18.2	370.64	*RPL4;TOP2A;RPL5;RPL3;RPS6;BUB1B;HMMR;RPL9;KIF15;EEF1A1;RPS27;RPS20;PABPC1;RPS27A;RPL19*
TTF2 22483619 ChIP-Seq HELA Human	11/1272	6.50e-09	1.37e-06	18.14	342.02	*RPL4;EEF1A1;RPL5;RPL3;RPS27;RPS6;RPS20;PABPC1;RPL9;CEP55;RPL19*
XRN2 22483619 ChIP-Seq HELA Human	11/1296	7.91e-09	1.37e-06	17.78	331.72	*RPL4;EEF1A1;RPL5;RPL3;RPS27;RPS6;RPS20;HMMR;PABPC1;RPL9;RPL19*
MYC 19079543 ChIP-ChIP MESCs Mouse	10/1013	1.23e-08	1.60e-06	18.92	344.54	*RPL4;EEF1A1;RPL5;RPL3;RPS27;RPS6;HMMR;RPS27A;RPL9;RPL19*

MYC 18358816 ChIP-ChIP MESCs Mouse	13/2369	3.03e-08	3.14e-06	13.89	240.52	*RPL4;TOP2A;RPL5;RPL3;RPS6;BUB1B;HMMR;RPL9;KIF15;RPS20;PABPC1;RPS27A;RPL19*
MYC 18555785 ChIP-Seq MESCs Mouse	9/852	4.87e-08	4.21e-06	18.57	312.73	*RPL4;TOP2A;EEF1A1;RPL5;RPL3;RPS27;RPS6;RPS20;RPS27A*
NELFA 20434984 ChIP-Seq ESCs Mouse	10/1454	3.76e-07	2.61e-05	12.84	189.9	*TOP2A;EEF1A1;RPL3;RPS27;RPS6;RPS20;PABPC1;RPS27A;CEP55;RPL19*
FOXM1 25889361 ChIP-Seq OE33 AND U2OS Human	8/784	4.46e-07	2.61e-05	16.5	241.27	*TOP2A;KIF14;BUB1B;HMMR;RPL9;CEP55;DLGAP5;KIF15*
AF4 26711339 ChIP-Seq SEM Human Blood Leukemia	13/2966	4.54e-07	2.61e-05	10.71	156.4	*RPL4;TOP2A;RPL5;RPS6;KIF14;BUB1B;RPL9;KIF15;EEF1A1;RPS27;RPS20;RPS27A;CEP55*
FOXM1 23109430 ChIP-Seq U2OS Human	5/226	2.38e-06	1.23e-04	29.8	385.9	*TOP2A;KIF14;HMMR;CEP55;DLGAP5*
FOXM1 26456572 ChIP-Seq MCF-7 Human BreastCancer	6/478	5.27e-06	2.48e-04	17.71	215.29	*TOP2A;RPL5;KIF14;RPS20;HMMR;CEP55*
ETS1 20019798 ChIP-Seq JURKAT Human	8/1332	2.32e-05	1.00e-03	9.39	100.26	*RPL5;RPL3;RPS27;RPS6;BUB1B;RPS20;RPL9;KIF15*
MYC 22102868 ChIP-Seq CA46 Human Blood BurkittsLymphoma	10/2314	2.59e-05	1.03e-03	7.67	81.01	*RPL4;EEF1A1;RPL5;RPL3;RPS27;RPS6;PABPC1;RPS27A;RPL9;RPL19*
ZFP42 18358816 ChIP-ChIP MESCs Mouse	7/1013	3.63e-05	1.34e-03	10.16	103.83	*RPL4;TOP2A;EEF1A1;RPL5;RPL3;HMMR;RPS27A*
E2F1 18555785 ChIP-Seq MESCs Mouse	11/3015	4.01e-05	1.35e-03	6.91	69.93	*RPL4;EEF1A1;RPL3;RPS27;RPS6;BUB1B;RPS20;RPS27A;CEP55;KIF15;RPL19*
MYC 28411283 ChIP-Seq MDA231-LM2-4175 Human BreastCancer	10/2441	4.16e-05	1.35e-03	7.22	72.83	*RPL4;EEF1A1;RPL5;RPL3;RPS27;RPS6;PABPC1;RPS27A;KIF15;RPL19*
MYCN 28898695 ChIP-Seq NB1643 Human Nerve Neuroblastoma	9/2184	1.18e-04	3.61e-03	6.7	60.56	*RPL4;EEF1A1;RPL5;RPL3;RPS20;RPS27A;RPL9;KIF15;RPL19*
BRD4 25478319 ChIP-Seq HGPS Human	8/1770	1.77e-04	5.08e-03	6.89	59.57	*RPL4;TOP2A;EEF1A1;RPS6;BUB1B;PABPC1;RPS27A;RPL19*
VDR 23849224 ChIP-Seq CD4+ Human	8/1811	2.07e-04	5.65e-03	6.72	57.0	*RPL5;RPL3;RPS27;RPS6;RPS20;PABPC1;RPS27A;RPL19*
EST1 17652178 ChIP-ChIP JURKAT Human	5/597	2.49e-04	6.45e-03	10.92	90.59	*RPS27;BUB1B;RPS27A;RPL9;KIF15*
E2F4 17652178 ChIP-ChIP JURKAT Human	5/608	2.71e-04	6.68e-03	10.71	87.98	*TOP2A;BUB1B;HMMR;RPS27A;KIF15*
CREB1 20920259 ChIP-Seq GC1-SPG Mouse	9/2451	2.90e-04	6.82e-03	5.88	47.87	*EEF1A1;RPL3;RPS27;BUB1B;PABPC1;RPL9;CEP55;KIF15;RPL19*
CHD1 19587682	5/626	3.10e	6.98e-03	10.39	83.95	*RPL4;RPL3;PABPC1;RPS27A;RPL19*

ChIP-ChIP MESCs Mouse		-04				
KDM6A 18722178 ChIP-ChIP U937 AND SAOS2 Human	4/384	5.08e -04	1.10e-02	12.89	97.81	*KIF14;BUB1B;RPS27A;KIF15*
CIITA 25753668 ChIP-Seq RAJI Human	4/392	5.49e -04	1.14e-02	12.62	94.77	*EEF1A1;CD74;RPS27;KIF15*
TAL1 20887958 ChIP-Seq HPC-7 Mouse	7/1584	5.95e -04	1.19e-02	6.28	46.67	*RPL4;EEF1A1;RPL3;RPS27;RPS6;RPS20;RPL19*
MYCN 18555785 ChIP-Seq MESCs Mouse	7/1668	8.12e -04	1.56e-02	5.94	42.26	*TOP2A;RPL3;RPS20;PABPC1;RPL9;KIF15;RPL19*
JARID1A 20064375 ChIP-Seq MESCs Mouse	7/1695	8.95e -04	1.66e-02	5.84	40.96	*RPL4;RPL3;RPS6;HMMR;RPL9;KIF15;RPL19*
YY1 33199912 ChIP-Seq 293T Human KidneyEmbryo	10/3612	1.15e -03	2.05e-02	4.55	30.79	*RPL4;EEF1A1;RPL5;RPL3;RPS27;RPS6;KIF14;BUB1B; PABPC1;KIF15*
KDM5B 21448134 ChIP-Seq MESCs Mouse	9/3030	1.42e -03	2.45e-02	4.59	30.12	*TOP2A;RPL5;KIF14;BUB1B;HMMR;PABPC1; RPS27A;DLGAP5;KIF15*
HNF1A 27111144 ChIP-Seq CD8+TCells Mouse Blood Lymphoma	9/3109	1.71e -03	2.86e-02	4.46	28.38	*TOP2A;EEF1A1;RPL3;RPS27;RPS6;RPS20;RPS27A; CEP55;KIF15*
MYBL2 22936984 ChIP-ChIP MESCs Mouse	6/1419	2.06e -03	3.33e-02	5.63	34.84	*TOP2A;EEF1A1;RPL3;BUB1B;HMMR;KIF15*
CCND1 20090754 ChIP-ChIP RETINA Mouse	6/1472	2.48e -03	3.84e-02	5.41	32.47	*RPL4;TOP2A;RPL3;HMMR;RPS27A;RPL19*
WDR5 24793694 ChIP-Seq LNCAP Human	4/592	2.52e -03	3.84e-02	8.24	49.32	*EEF1A1;CD74;RPS6;PABPC1*
EKLF 21900194 ChIP-Seq ERYTHROCYTE Mouse	5/1055	3.23e -03	4.78e-02	6.01	34.47	*RPL4;EEF1A1;RPL3;RPS27A;RPL19*

Adjusted P-values were computed using the Benjamini–Hochberg false-discovery-rate correction. The combined score was calculated by Enrichr as the product of the natural logarithm of the P-value and the z-score. Overlap is expressed as the number of input genes overlapping the transcription-factor target set divided by the total number of genes in that target set.

Table S39. ENCODE-ChEA Consensus transcription-factor enrichment analysis of CytoHubba MCC-prioritized hub genes associated with ligamentum flavum hypertrophy.

Transcription Factor / Dataset	Overlap	P-value	Adjusted P-value	Odds Ratio	Combined Score	Overlapping Genes
MYC CHEA	8/573	4.02e-08	1.27e-06	22.91	390.12	*RPL4;RPL5;RPL3;RPS27;RPS6;PABPC1;RPS27A;RPL19*
MYC ENCODE	11/1515	4.03e-08	1.27e-06	15.01	255.66	*RPL4;EEF1A1;RPL5;RPL3;RPS6;BUB1B;HMMR;PABPC1; RPS27A;RPL9;RPL19*
NELFE ENCODE	6/234	8.13e-08	1.49e-06	37.13	606.12	*EEF1A1;RPL3;RPS27;RPS6;RPS20;PABPC1*

Transcription Factor / Dataset	Overlap	P-value	Adjusted P-value	Odds Ratio	Combined Score	Overlapping Genes
TAF7 ENCODE	8/640	9.43e-08	1.49e-06	20.41	330.15	*RPL4;EEF1A1;RPL5;RPL3;RPS27;RPS6;RPS20;PABPC1*
KAT2A ENCODE	4/90	1.76e-06	2.21e-05	57.83	766.35	*RPL4;RPL5;RPS6;RPL9*
ATF2 ENCODE	12/2852	2.85e-06	2.99e-05	9.05	115.6	*RPL4;EEF1A1;RPL5;RPL3;RPS27;RPS6;RPS20;HMMR; RPS27A;RPL9;KIF15;RPL19*
E2F4 ENCODE	7/710	3.57e-06	3.21e-05	14.77	185.2	*TOP2A;KIF14;BUB1B;RPS20;CEP55;DLGAP5;KIF15*
SIN3A ENCODE	8/1131	6.97e-06	5.49e-05	11.19	132.91	*RPL4;EEF1A1;BUB1B;HMMR;PABPC1;CEP55;DLGAP5;KIF15*
MAX ENCODE	10/2073	9.72e-06	6.80e-05	8.68	100.24	*RPL4;RPL5;RPS6;BUB1B;RPS20;PABPC1;RPS27A; CEP55;KIF15;RPL19*
RELA ENCODE	5/484	9.33e-05	5.88e-04	13.57	125.93	*EEF1A1;RPL5;CD74;RPS6;RPS27A*
TAF1 ENCODE	11/3346	1.08e-04	6.17e-04	6.1	55.73	*RPL4;EEF1A1;RPL5;RPL3;RPS27;RPS6;RPS20; PABPC1;RPS27A;RPL9;RPL19*
CREB1 ENCODE	9/2238	1.43e-04	7.52e-04	6.52	57.67	*EEF1A1;RPL5;CD74;RPL3;RPS27;KIF14;PABPC1; RPL9;KIF15*
ELF1 ENCODE	9/2483	3.20e-04	1.55e-03	5.79	46.59	*RPL4;RPL3;RPS27;RPS6;KIF14;PABPC1;RPS27A; CEP55;RPL19*
CEBPD ENCODE	5/734	6.43e-04	2.89e-03	8.8	64.69	*EEF1A1;RPS27;RPS6;RPS20;RPL9*
FOXM1 ENCODE	2/95	4.01e-03	1.65e-02	23.76	131.12	*KIF14;CEP55*
YY1 CHEA	3/332	4.19e-03	1.65e-02	10.54	57.71	*RPL4;EEF1A1;RPS27*
TCF3 ENCODE	4/840	8.74e-03	3.24e-02	5.72	27.13	*EEF1A1;RPL3;RPS6;RPS20*
E2F1 CHEA	4/859	9.44e-03	3.31e-02	5.59	26.07	*RPL3;BUB1B;RPS20;DLGAP5*
CREB1 CHEA	5/1444	1.21e-02	4.01e-02	4.29	18.96	*EEF1A1;RPS27;PABPC1;CEP55;KIF15*
YY1 ENCODE	7/2753	1.40e-02	4.41e-02	3.38	14.43	*RPL4;EEF1A1;RPL5;RPL3;RPS27;BUB1B;KIF15*

Adjusted P-values were computed using the Benjamini–Hochberg false-discovery-rate correction. The combined score was calculated by Enrichr as the product of the natural logarithm of the P-value and the z-score. “CHEA” and “ENCODE” denote the supporting ChIP evidence source for each transcription factor.
